# Degeneracy in hippocampal physiology and plasticity

**DOI:** 10.1002/hipo.23139

**Published:** 2019-07-13

**Authors:** Rahul K. Rathour, Rishikesh Narayanan

**Affiliations:** ^1^ Cellular Neurophysiology Laboratory Molecular Biophysics Unit, Indian Institute of Science Bangalore India

**Keywords:** Degeneracy, learning, memory, encoding, homeostasis, causality, reductionism, holism, structure‐function relationships, variability, compensation

## Abstract

Degeneracy, defined as the ability of structurally disparate elements to perform analogous function, has largely been assessed from the perspective of maintaining robustness of physiology or plasticity. How does the framework of degeneracy assimilate into an encoding system where the ability to change is an essential ingredient for storing new incoming information? Could degeneracy maintain the balance between the apparently contradictory goals of the need to change for encoding and the need to resist change towards maintaining homeostasis? In this review, we explore these fundamental questions with the mammalian hippocampus as an example encoding system. We systematically catalog lines of evidence, spanning multiple scales of analysis that point to the expression of degeneracy in hippocampal physiology and plasticity. We assess the potential of degeneracy as a framework to achieve the conjoint goals of encoding and homeostasis without cross‐interferences. We postulate that biological complexity, involving interactions among the numerous parameters spanning different scales of analysis, could establish disparate routes towards accomplishing these conjoint goals. These disparate routes then provide several degrees of freedom to the encoding‐homeostasis system in accomplishing its tasks in an input‐ and state‐dependent manner. Finally, the expression of degeneracy spanning multiple scales offers an ideal reconciliation to several outstanding controversies, through the recognition that the seemingly contradictory disparate observations are merely alternate routes that the system might recruit towards accomplishment of its goals.

## 1 INTRODUCTION

The pervasive question on the relationship between structure and function spans every aspect of life, science and philosophy: from building architectures to the mind‐body problem, from connectomics to genomics to proteomics, from subatomic structures to cosmic bodies and from biomechanics to climate science. Even within a limited perspective spanning only neuroscience, the question has been posed at every scale of brain organization spanning the genetic to behavioral ends of the spectrum. Efforts to address this question have resulted in extensive studies that have yielded insights about the critical roles of protein structure and localization, synaptic ultrastructure, dendritic morphology, microcircuit organization and large‐scale synaptic connectivity in several neural and behavioral functions.

The question on the relationship between structure and function has spawned wide‐ranging debates, with disparate approaches towards potential answers. At one extreme is the suggestion that structure defines function (Buzsaki, 2006):

“The safest way to start speculating about the functions of a structure is to inspect its anatomical organization carefully. The dictum “structure defines function” never fails, although the architecture in itself is hardly ever sufficient to provide all the necessary clues.”

Within this framework, the following is considered as a route for understanding neural systems and behavior (Buzsaki, 2006):

“First, we need to know the basic “design” of its circuitry at both microscopic and macroscopic levels. Second, we must decipher the rules governing interactions among neurons and neuronal systems that give rise to overt and covert behaviors.”

The other extreme is the assertion that “form follows function”, elucidated by Bert Sakmann (Sakmann, 2017), quoting Louis Sullivan:

“Whether it be the sweeping eagle in his flight, or the open apple‐blossom, the toiling work‐horse, the blithe swan, the branching oak, the winding stream at its base, the drifting clouds, over all the coursing sun, form ever follows function, and this is the law. Where function does not change, form does not change”.

Within this framework, the approach to understanding neural structure function relations was elucidated as (Sakmann, 2017):

“The approach we took, in order to discover structure‐function relations that help to unravel simple design principles of cortical networks was, to first determine functions and then reconstruct the underlying morphology assuming that “form follows function”, a dictum of Louis Sullivan and also a Bauhaus design principle.”

A third approach embarks on addressing the structure‐function question by recognizing the existence of ubiquitous variability and combinatorial complexity in biological systems. This was elucidated in a landmark review by Edelman and Gally, who presented an approach to structure‐function relationship by defining degeneracy (Edelman & Gally, 2001):

“Degeneracy is the ability of elements that are structurally different to perform the same function or yield the same output. Unlike redundancy, which occurs when the same function is performed by identical elements, degeneracy, which involves structurally different elements, may yield the same or different functions depending on the context in which it is expressed. It is a prominent property of gene networks, neural networks, and evolution itself. Indeed, there is mounting evidence that degeneracy is a ubiquitous property of biological systems at all levels of organization.”

They approach degeneracy and the structure‐function question from an evolutionary perspective, noting (Edelman & Gally, 2001):

"Here, we point out that degeneracy is a ubiquitous biological property and argue that it is a feature of complexity at genetic, cellular, system, and population levels. Furthermore, it is both necessary for, and an inevitable outcome of, natural selection.”

From this perspective, the supposition that a one‐to‐one relationship between structure and function exists is eliminated, thereby yielding more structural routes to achieving the same function. This perspective posits that biological complexity should be viewed from the evolutionarily advantageous perspective of providing functional robustness through degeneracy. Further, the degeneracy framework provides the system with higher degrees of freedom to recruit a state‐dependent solution from a large repertoire of routes that are available to achieve the same function.

The advantages of biological variability (Foster, Ungar, & Schwaber, 1993; Gjorgjieva, Drion, & Marder, 2016; Goldman, Golowasch, Marder, & Abbott, 2001; Katz, 2016; Marder, 2011; Marder & Goaillard, 2006; Marder, Goeritz, & Otopalik, 2015; Marder & Taylor, 2011; O'Leary & Marder, 2014; Prinz, Bucher, & Marder, 2004; ), degeneracy (Drion, O'Leary, & Marder, 2015; Edelman & Gally, 2001; Leonardo, 2005; O'Leary, Williams, Caplan, & Marder, 2013; Whitacre & Bender, 2010; Whitacre, 2010) and complexity (Carlson & Doyle, 2002; Edelman & Gally, 2001; Stelling, Sauer, Szallasi, Doyle 3rd, & Doyle, 2004; Tononi, Sporns, & Edelman, 1996; Tononi, Sporns, & Edelman, 1999; Weng, Bhalla, & Iyengar, 1999; Whitacre, 2010), especially in terms of their roles in achieving robust function, have been widely studied and recognized in several biological process, including those in simple nervous systems. However, this recognition has been very limited in the mammalian neuroscience literature, a literature where the focus is predominantly on explicitly assigning (or implicitly assuming) unique causal mechanistic relationships between constituent components and emergent functions. Here, we focus on the mammalian hippocampus, a brain region that has been implicated in spatial cognition, learning and memory, and review several lines of evidence that point to the existence of degeneracy in hippocampal physiology and plasticity. We argue that the elucidation of degeneracy spanning multiple scales could result in resolution of several existing controversies in the field, and provide an ideal setup to design experiments to understand neuronal systems, their adaptability and their responses to pathological insults.

The rest of the review is organized into four sections. In the first of these sections, we explore the foundations of degeneracy, especially from a perspective of an encoding system such as the hippocampus, and outline distinctions between different forms of homeostasis and their interactions with encoding‐induced adaptations. In the second section, we build an argument that theoretical and experimental literature, spanning multiple scales of analysis, presents abundant support for the prevalence of degeneracy in almost all aspects of hippocampal physiology and plasticity. The third section explores the important question on the feasibility of establishing one‐to‐one structure‐function relationships in systems that exhibit degeneracy through complexity. The final section concludes the review by briefly summarizing the arguments and postulates presented here on degeneracy in encoding within the degeneracy framework.

## 2 DEGENERACY: FOUNDATIONS FROM THE PERSPECTIVE OF AN ENCODING SYSTEM

**Figure 1:**
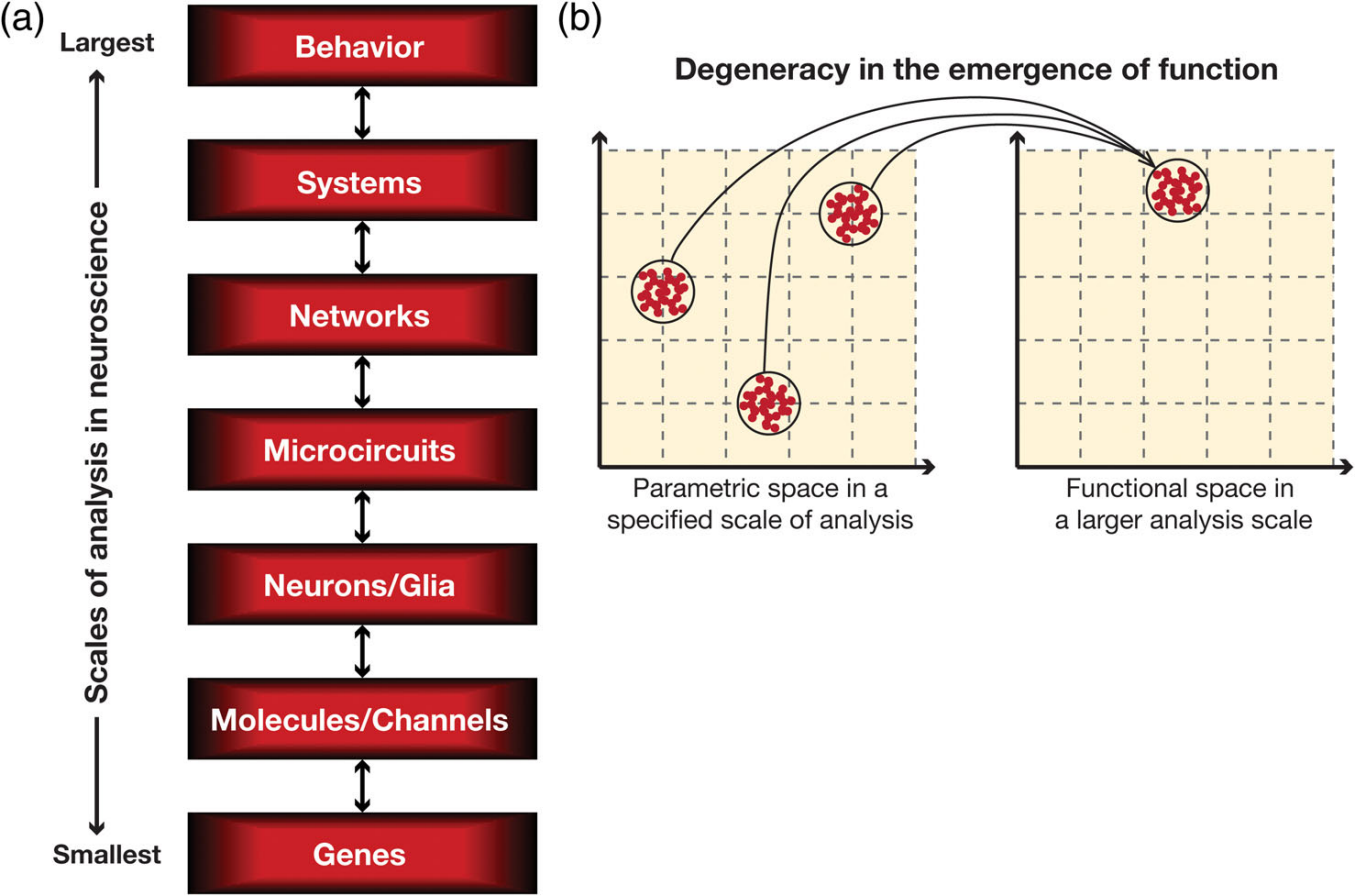
Degeneracy in the emergence of a function and its robustness to external perturbation across multiple scales of analysis. (a) Representation of multiple scales of analysis in neuroscience. The size (large and small) of the scale of analysis is representative of size of the constitutive components in that scale (Churchland & Sejnowski, 1992; Churchland & Sejnowski, 1988). (b) Disparate combinations of parameters in a specified scale of analysis could result in similar function in a larger scale of analysis. Each red circle in the smaller scale of analysis represents a combination of parameters that results in a specified function in large analysis scale, also represented by red circles there. The enclosing black circle in the larger scale represents experimentally observed variability in the function that is being assessed. On the other hand, the black circle in the smaller scale illustrates that robust functionality in the larger scale could be achieved even with small local perturbations in the parametric space. Larger perturbations beyond the black circle, however, would not yield robust functionality. The presence of multiple clusters of red circles in the smaller scale represents degeneracy, where similar functionality is achieved if parameters are within any of those multiple clusters [Color figure can be viewed at wileyonlinelibrary.com]

Akin to the much broader span of physics from the subatomic to the cosmic scales, and very similar to studies on other biological systems, neural systems are studied at multiple scales of analysis (Figure 1a). Although understanding neural systems *within* each of these scales of analysis is critical and has its own right for existence, a predominant proportion of neuroscientific research is expended on *cross‐scale emergence* of function through *interactions* among constituent components. One set of studies focus on the emergence of functions in a specified scale of analysis as a consequence of interactions among components in the immediately lower scale of analysis. An elegant example to such analysis is on the emergence of neuronal action potentials (a cellular scale function) as a consequence of interactions (Hodgkin & Huxley, 1952) between sodium and delayed rectifier potassium channels (molecular scale components). Another set of studies focus on the relationships between function at a specified scale of analysis and components that are integral to a scale that is several levels apart. With specific reference to the hippocampus, assessing the molecular‐ or cellular‐scale components (e.g., receptors, synapses) that are *causally* responsible for learning and memory (a behavioral scale function that is several scales apart from the molecular/cellular scales) forms an ideal example for studies that belong in this category (Bliss & Collingridge, 1993; Kandel, Dudai, & Mayford, 2014; Martin, Grimwood, & Morris, 2000; Mayford, Siegelbaum, & Kandel, 2012; Neves, Cooke, & Bliss, 2008).

Healthy and invigorating debates related to the philosophical and the scientific basis of such analyses, with themes ranging from broad discussions on reductionism versus holism (Bennett & Hacker, 2003; Bickle, 2015; Jazayeri & Afraz, 2017; Krakauer, Ghazanfar, Gomez‐Marin, MacIver, & Poeppel, 2017; Panzeri, Harvey, Piasini, Latham, & Fellin, 2017) to more focused debates on the specific cellular components that are involved in specific aspects of coding and behavior (Bliss & Collingridge, 1993; Gallistel, 2017; Kandel et al., 2014; Kim & Linden, 2007; Martin et al., 2000; Mayford et al., 2012; Mozzachiodi & Byrne, 2010; Neves, Cooke, & Bliss, 2008; Otchy et al., 2015; Titley, Brunel, & Hansel, 2017; Zhang & Linden, 2003), have contributed to our emerging understanding of neural systems and their links to behavior. Several studies have covered the breadth and depth of these debates (Bargmann & Marder, 2013; Bennett & Hacker, 2003; Bickle, 2015; Jazayeri & Afraz, 2017; Jonas & Kording, 2017; Kandel et al., 2014; Katz, 2016; Kim & Linden, 2007; Krakauer et al., 2017; Lazebnik, 2002; Marder, 1998; Marder, 2011; Marder, 2012; Marder, O'Leary, & Shruti, 2014; Marder & Thirumalai, 2002; Mayford et al., 2012; Panzeri et al., 2017; Tytell, Holmes, & Cohen, 2011), and will not be the focus of this review.

Within the purview of degeneracy, the emergence of specific combinations of higher‐scale functions (within the limits of biological variability) could be achieved (Figure 1b) through interactions among disparate parametric combinations in a lower scale (Basak & Narayanan, 2018b; Edelman & Gally, 2001; Foster et al., 1993; Gjorgjieva et al., 2016; Goldman et al., 2001; Marder, 2011; Marder & Goaillard, 2006; Marder et al., 2015; Marder & Taylor, 2011; Mishra & Narayanan, 2019; Mittal & Narayanan, 2018; O'Leary & Marder, 2014; Prinz et al., 2004; Rathour & Narayanan, 2012a; Rathour & Narayanan, 2014; Srikanth & Narayanan, 2015; Stelling et al., 2004; Taylor et al., 2009). A straightforward corollary to this is that robust homeostasis in the maintenance of specific combinations of higher‐scale functions in the face of perturbations there would be achieved through very different routes involving disparate parametric combinations in a lower scale. For instance, a change in neuronal firing rate at the cellular scale owing to external perturbations involving pathological insults or behavioral experience could be compensated for by different sets of changes to synaptic or intrinsic parameters (at the molecular scale) to achieve activity homeostasis (Gjorgjieva et al., 2016; Hengen, Torrado Pacheco, McGregor, Van Hooser, & Turrigiano, 2016; Nelson & Turrigiano, 2008; Turrigiano, 2011; Turrigiano, 1999; Turrigiano, 2008; Turrigiano & Nelson, 2004). Thus, under the degeneracy framework, different uncorrelated clusters in the lower‐scale parametric space could result in similar, if not identical, functional outcomes in the higher‐scale measurement space, thereby suggesting a many‐to‐one relationship between the lower‐scale parameters and higher‐scale measurements (Edelman & Gally, 2001; Jazayeri & Afraz, 2017; Krakauer et al., 2017). Prominent lines of experimental evidence in support of degeneracy in neural systems have come from demonstrations of remarkable animal‐to‐animal variability in constituent components in providing analogous functional outcomes, and/or from results on many‐to‐many mappings between neural activity and behavior (Marder, 2011; Marder & Goaillard, 2006; Marder & Taylor, 2011; O'Leary & Marder, 2014; Schulz, Goaillard, & Marder, 2006; Schulz, Goaillard, & Marder, 2007; Vogelstein et al., 2014).

### 2.1 Degeneracy versus compensation

A common misconception relating to degeneracy is that systems exhibiting degeneracy should compensate for the removal of a specific lower‐scale component by recruiting other structural components there to yield the same higher‐scale function. A corollary to this misconception is that an inability to compensate for the removal of a component is interpreted as evidence for the absence of degeneracy. For instance, consider an experiment where the “usefulness” of a specific gene is being tested by assessing deficits in a specific behavior after knockout of the gene under consideration. If the knockout resulted in the behavioral deficit, degeneracy is determined to be absent and the gene considered essential. On the other hand, for the case where there was no behavioral deficit, the gene is either considered nonessential or the result is interpreted as the expression of degeneracy where other components have compensated for the knockout.

There have been several warnings against such oversimplified interpretations, especially considering that biological systems are dynamic adaptive systems and not static (Edelman & Gally, 2001; Grashow, Brookings, & Marder, 2010; Marder, 2011; Marder & Goaillard, 2006; Marder & Taylor, 2011; O'Leary, Williams, Franci, & Marder, 2014; Taylor et al., 2009; Wagner, 2005). Specifically, although the biological system adapts to the “unplanned” absence of the single gene (Edelman & Gally, 2001), it is not always essential that the adaptations result in compensation of one specific behavioral readout [of the several possible readouts (Mayford et al., 2012; Jazayeri & Afraz, 2017; Krakauer et al., 2017)]. Any compensation has been argued as a statistical result of the tradeoffs that are inherent to this complex, adaptive and nonlinear system that manifests degeneracy that is *emergent* across multiple scales of organization (Edelman & Gally, 2001; O'Leary et al., 2014). It has also been postulated that the compensatory process, and not the deletion, could have resulted in a specific deficit (O'Leary et al., 2014); especially because of the remarkable dissociation between different forms of homeostasis (see Section 2.2).

Further, especially given the ubiquitous variability across animals in terms of constituent components that elicit analogous behavior, it is clear that the impact of deletion of one specific component would be differential. This implies that the simplistic generalizability on the presence or absence of degeneracy based on a single parameter and a single measurement is untenable in complex adaptive systems. Additionally, with reference to the specific example of gene deletion, it is also important to distinguish between the acute impact of a lack of a protein that is tied to the gene and the developmental knockout (and associated compensatory mechanisms) of the specified gene (Edelman & Gally, 2001; Grashow et al., 2010; Marder, 2011; Marder & Goaillard, 2006; Marder & Taylor, 2011; O'Leary et al., 2014; Taylor et al., 2009).

**Figure 2:**
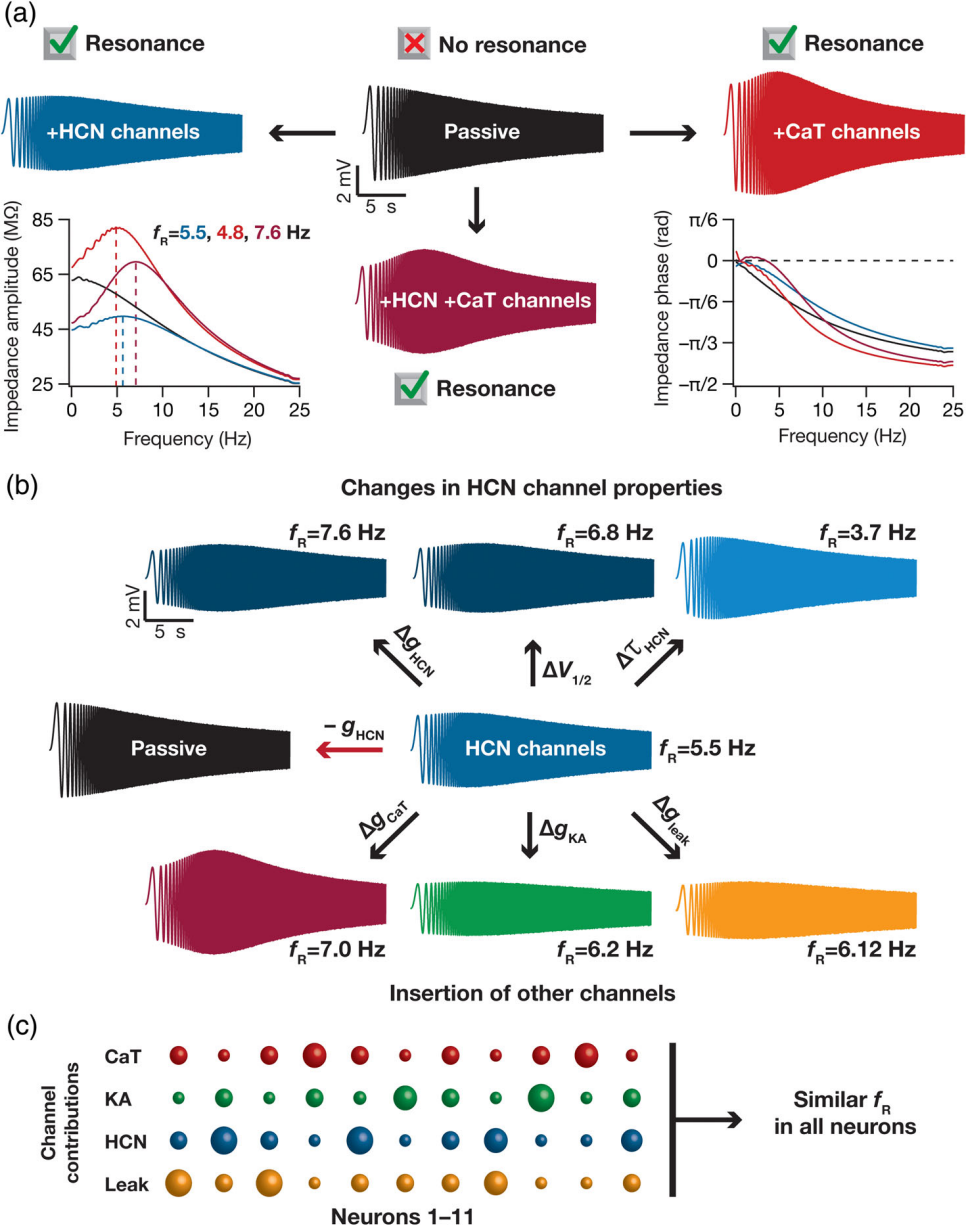
Qualitative versus quantitative degeneracy. (a) Qualitative degeneracy, where the functional goal on which degeneracy is assessed is the expression of resonance, which could be achieved by the presence of one or more resonating conductances. Depicted are voltage traces obtained in response to a chirp current injection into neurons containing none, one or two resonating conductances. The hyperpolarization‐activated cyclic‐nucleotide gated (HCN) and *T*‐type calcium (CaT) are employed as the two example resonating conductances. In a neuron, that expresses two or more resonating conductances (at sufficient densities), resonance ceases to express only when both resonating conductances are eliminated. The impedance amplitude (left bottom) and phase profiles (right bottom) are also shown for each color‐matched chirp response. It may be noted that resonance in the amplitude profile and lead in the phase profile are observed when resonating conductances are expressed individually or together, and synergistically interact when they are expressed together. (b) Quantitative degeneracy, where the functional goal on which degeneracy is assessed is the ability to specify a target value of resonance frequency in the neuron, when a resonating conductance is expressed. Shown are some examples of the disparate possible routes to achieve quantitative changes to resonance frequency. One set of possibilities involves altering the properties of the channel mediating resonance (taken to be HCN in this example) such as its density (Δ*g*_HCN_), its gating properties (e.g., half‐maximal activation voltage, Δ*V*_1/2_) or its kinetics (e.g., activation time constant, Δ*τ*_HCN_). The other set involves introducing (e.g., *T*‐type calcium channels, Δ*g*_CaT_ or *A*‐type potassium channels, Δ*g*_KA_) or altering (e.g., change in leak channels Δ*g*_leak_) other channels that modulate the resonance mediated by the resonating conductance (whose removal would abolish resonance, –*g*_HCN_, unless compensated by the expression of another resonating conductance). (c) In different neurons, the contribution of different channels to any measurement (shown here is resonance frequency, *f*_R_) could be variable. The size of each sphere scales with the quantum of contribution of a given channel (one among HCN, CaT, KA, and leak) to *f*_R_ in a given neuron (11 neurons are depicted). Traces presented here and associated conclusions are drawn from previous studies (Hutcheon & Yarom, 2000; Narayanan & Johnston, 2007; Narayanan & Johnston, 2008; Rathour, Malik, & Narayanan, 2016; Rathour & Narayanan, 2012a) [Color figure can be viewed at wileyonlinelibrary.com]

In addition to these strong arguments against a one‐to‐one link between compensation and degeneracy, it is also important to consider the specifics of the expectations on the specific function that degeneracy is defined for and what functional deficit is to be compensated. Let us consider the example of the emergence of membrane potential resonance in neurons as an example to illustrate this argument (Figure 2). The emergence of resonance requires the expression of a resonating conductance, and the biophysical constraints on what makes a resonating conductance are well established (Cole, 1968; Das, Rathour, & Narayanan, 2017; Hodgkin & Huxley, 1952; Hutcheon & Yarom, 2000; Mauro, 1961; Mauro, Conti, Dodge, & Schor, 1970; Narayanan & Johnston, 2008). Hippocampal pyramidal neurons express several resonating conductances: the hyperpolarization‐activated cyclic nucleotide‐gated (HCN) nonspecific cation channels, the *M*‐type potassium (KM) channels and the *T*‐type calcium (CaT) channels, of which HCN and CaT channels exhibit overlapping voltage dependencies (Das et al., 2017; Hu, Vervaeke, Graham, & Storm, 2009; Hu, Vervaeke, & Storm, 2002; Narayanan & Johnston, 2007; Narayanan & Johnston, 2008; Pike et al., 2000; Rathour & Narayanan, 2012a).

Let us first consider an example where the function on which degeneracy is assessed is qualitatively defined as the *expression* of membrane potential resonance (Figure 2a). Whereas a passive neuron does not express resonance, the presence of the HCN and/or the CaT channels would result in the expression of resonance. This implies degeneracy in the function, where similar functionality (in this case, the expression of resonance) is achieved through disparate components (channel combinations). In this scenario, depending on the variable expression profiles of HCN, CaT and other modulating channels, removal of only one of them could still result in the expression of resonance in specific neurons (Das et al., 2017; Rathour et al., 2016; Rathour & Narayanan, 2012a; Rathour & Narayanan, 2014). However, removal of both HCN and CaT channels would result in a deficit in the assessed function, where resonance ceases to express. Here, the requirement or usefulness of HCN or CaT channels to the expression of resonance is easily identified by acute blockade experiments, although it would be difficult to predict (a) synergy between different channels that are expressed towards the emergence of resonance with such one‐channel‐at‐a‐time pharmacological blockade experiments; and (b) possible compensatory mechanisms involving changes in kinetics or voltage‐dependence properties of other channels, say KM channels, in a double knockout scenario (Marder, 2011; Marder & Goaillard, 2006; O'Leary et al., 2014; Rathour & Narayanan, 2012a; Rathour & Narayanan, 2014; Taylor et al., 2009).

In most encoding or homeostatic scenarios involving changes in constituent components, however, the functional outcome that is expected is a more quantitative readout of, say, firing rate or calcium concentration altered or returned to *specific values*. Therefore, a widely employed alternate interpretation (Basak & Narayanan, 2018b; Foster et al., 1993; Goldman et al., 2001; Marder, 2011; Marder & Goaillard, 2006; Marder et al., 2015; Marder & Taylor, 2011; Migliore et al., 2018; Mishra & Narayanan, 2019; Mittal & Narayanan, 2018; Prinz et al., 2004; Rathour & Narayanan, 2012a; Rathour & Narayanan, 2014; Srikanth & Narayanan, 2015; Taylor et al., 2009) is where degeneracy is assessed as the ability of different structural components to elicit *quantitatively* similar functional measurements. With reference to our chosen example, this would translate to assessing degeneracy as the ability to achieve a *specific range of values* of resonance frequency with disparate combinations of parameters (Figure 2b). If achieving a specific range of resonance frequency was the functional goal, and not the qualitative expression of resonance, then the possibilities are numerous. A resonating conductance is indeed required for the expression of resonance (Figure 2b), but the goal is not to understand the expression of resonance, but to maintain resonance frequency at a specific value. In the presence of a resonating conductance, this goal could be achieved through very different structural routes either by altering other channel conductances or by altering properties of the resonating conductance itself (Das et al., 2017; Hu et al., 2002; Narayanan & Johnston, 2007; Narayanan & Johnston, 2008; Rathour et al., 2016; Rathour & Narayanan, 2012a; Rathour & Narayanan, 2014; Zemankovics, Kali, Paulsen, Freund, & Hajos, 2010). This implies the expression of degeneracy, where disparate parametric combinations could yield *quantitatively* similar resonance frequencies (Rathour & Narayanan, 2012a; Rathour & Narayanan, 2014) across different models (Figure 2c). Importantly, the order of degeneracy is rather large with the several active and passive properties, with the conductances, the voltage‐dependence and kinetic properties of each of the several channels included. This also provides several routes to the emergence of compensation, where different channels and different parameters could differentially contribute to the emergence of similar functional measurements (Figure 2c). We argue that this *quantitative* scenario with a large order of degeneracy is closer to the requirements of a system (at any given scale of organization) from the perspective of equilibrium and sustenance. The relevance of the qualitative scenario is rather limited to experiments that merely probe the expression of a specific phenomenon in a binary fashion (expressing or not) (Edelman & Gally, 2001).

Together, the question on the link between degeneracy and compensation should not be treated with simplistic ideas of linear interactions across components in a nonadapting system. The analyses should account for the specific definition of the function under consideration and the question on how degeneracy is defined. In addition, the nonlinear and synergistic interactions between different components that result in the specific function and animal‐to‐animal variability in expression profiles of constituent components should be assessed as part of such analyses. Finally, the possibility that “stochastic” compensatory process could be homeostatic or pathological and the relevance of the challenge that is being posed to the system to evolutionary convergence should also be considered (Edelman & Gally, 2001; Grashow et al., 2010; Marder, 2011; Marder & Taylor, 2011; O'Leary et al., 2014; Taylor et al., 2009).

### 2.2 Dissociation between different forms of homeostasis

It is clear from the examples presented above that the specific functional readout for which robustness or homeostasis ought to be maintained is a very critical question within the framework of degeneracy. Although degeneracy can be defined or observed with reference to any function at any scale of organization, the answer to the question on what specific functional homeostasis is absolutely essential from an evolutionary/neuroethological perspective is not clear. Even with reference to individual neurons, the literature has defined several forms of homeostasis (Gjorgjieva et al., 2016; Nelson & Turrigiano, 2008; Turrigiano, 2011; Turrigiano, 2008; Turrigiano & Nelson, 2004), with popular measures involving neuronal firing rate (Hengen et al., 2016), cytosolic calcium (Honnuraiah & Narayanan, 2013; O'Leary et al., 2014; Siegel, Marder, & Abbott, 1994; Srikanth & Narayanan, 2015) or excitation‐inhibition balance (Yizhar et al., 2011). In addition, despite perpetual changes in afferent activity under in vivo conditions (Buzsaki, 2002; Buzsaki, 2006; Buzsaki, 2015; Srikanth & Narayanan, 2015; Tononi & Cirelli, 2006), specific neuronal subtypes maintain distinct functional signatures, say in terms of their excitability or oscillatory or frequency selectivity measurements, that are different from other neuronal subtypes even in the same brain region (Hoffman, Magee, Colbert, & Johnston, 1997; Migliore & Shepherd, 2002; Migliore & Shepherd, 2005; Narayanan & Johnston, 2007; Narayanan & Johnston, 2008; Pike et al., 2000; Spruston, 2008; Zemankovics et al., 2010). Further, synaptic properties such as strength and release probabilities are also very discernable across different synaptic subtypes (say excitatory *vs*. inhibitory) even on the same postsynaptic neuron (Andrasfalvy & Magee, 2001; Andrasfalvy & Mody, 2006; Dittman, Kreitzer, & Regehr, 2000; Koester & Johnston, 2005; Magee & Cook, 2000; Smith, Ellis‐Davies, & Magee, 2003). This suggests the existence of some form of homeostasis that maintains these intrinsic and synaptic measurements, including or apart from firing rate or calcium homeostasis or excitatory‐inhibitory balance, despite behaviorally driven encoding changes or perpetual activity switches that are common in the hippocampus and other regions of the brain. Does maintenance of one of them translate to maintenance of all of them? If not, which of these different forms of homeostasis are absolutely essential for the animal from the evolutionary/neuroethological perspective?

**Figure 3:**
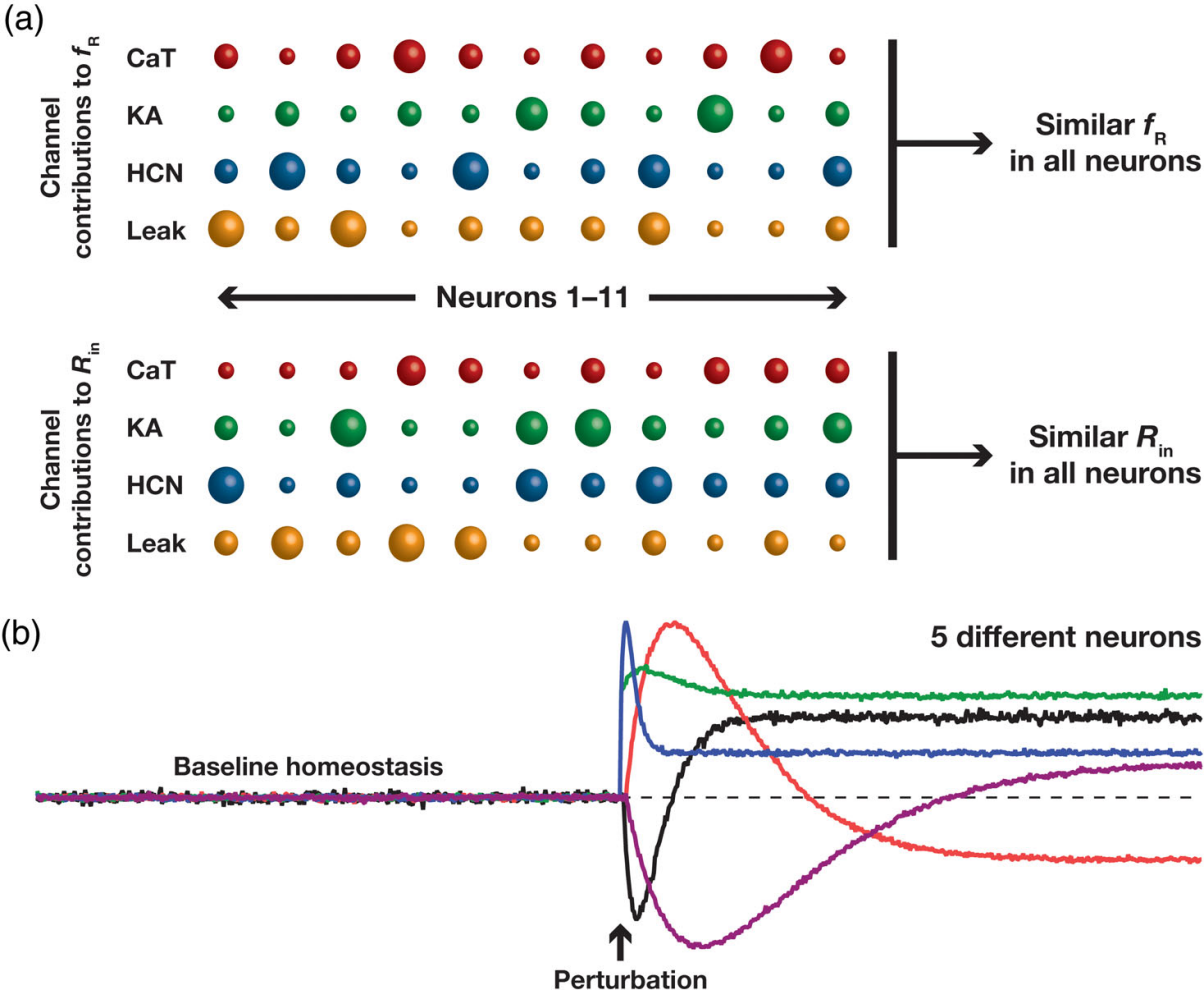
Dissociation between different forms of homeostasis. (a) In different neurons, the contribution of different channels to different measurements (shown here are resonance frequency, *f*_R_, and input resistance, *R*_in_) is differential and variable. The size of each sphere scales with the quantum of contribution of a given channel (one among HCN, CaT, KA, and leak) to *f*_R_ in a given neuron (11 neurons are depicted). It may be noted that in any given neuron, it is not necessary that the contributions of any given channel to *f*_R_ and *R*_in_ need not be equal, even when both *f*_R_ and *R*_in_ are similar across all neurons. Cartoon illustrations are derived from data presented in previous studies (Rathour et al., 2016; Rathour & Narayanan, 2012a; Rathour & Narayanan, 2014; Srikanth & Narayanan, 2015). (b) Although baseline homeostasis is efficaciously maintained in five different neurons, their responses to an identical perturbation need not necessarily be identical or even similar. The perturbation could be a plasticity‐inducing stimulus driven by behavioral experience or by pathological conditions. Cartoon illustration was derived from analyses presented in previous studies (Anirudhan & Narayanan, 2015; O'Leary et al., 2014; Srikanth & Narayanan, 2015). HCN, hyperpolarization‐activated cyclic‐nucleotide gated [Color figure can be viewed at wileyonlinelibrary.com]

There are several lines of clear evidence that there are remarkable dissociations between different forms of homeostasis (Srikanth & Narayanan, 2015). First, cellular‐ or network‐scale functions could robustly emerge with disparate combinations of molecular‐ or cellular‐scale parameters (Foster et al., 1993; Marder, 2011; Marder & Goaillard, 2006; Prinz et al., 2004; Rathour & Narayanan, 2014; Taylor et al., 2009). These observations suggest that precise homeostatic balance at a lower scale (e.g., ion channels expressed to exact conductance values) is not essential for maintaining functional homeostasis at a higher scale. Second, even in the same set of neurons/networks/animals, different measurements have different dependencies on underlying parameters, and these dependencies could be variable. For instance, in the same neuron, resonance frequency could have a larger dependence on one channel subtype with input resistance being critically regulated by another channel, with the specifics of these dependencies variable across different neurons of the same subtype (Figure 3a). Studies have shown that different channels could have differential and variable impact on disparate measurements from the same neuron, even in a location dependent manner (Grashow et al., 2010; O'Leary et al., 2014; Rathour & Narayanan, 2014; Taylor et al., 2009). Additionally, acute blockade of one specific channel results in weakly correlated changes in different measurements in the same neuron (Rathour et al., 2016). This implies that changing individual constitutive components to maintain robust homeostasis in one of the measurements does not necessarily translate to robust homeostasis in all the other measurements.

Third, for maintenance of calcium homeostasis across neurons in a network or in neurons that are subjected to perpetual switches in afferent activity, it is not essential that functional homeostasis across different intrinsic or synaptic measurements is maintained. Specifically, owing to inherent variability in different constitutive components, the channel conductance values, neuronal intrinsic properties, or synaptic strengths could be very different across different neurons despite maintenance of precise calcium homeostasis in neurons or their network (Gjorgjieva et al., 2016; O'Leary et al., 2014; Srikanth & Narayanan, 2015). Finally, calcium and firing rate homeostasis have been shown to be dissociated whereby tremendous variability in channel conductance values, firing rate and pattern of firing have been observed despite efficacious maintenance of calcium homeostasis (O'Leary et al., 2013; O'Leary et al., 2014; Srikanth & Narayanan, 2015). Together, these studies establish that none of the individual forms of homeostasis (in calcium concentration or in channel densities channel or in intrinsic functional characteristics including neuronal firing‐rate) necessarily translate to or follow from any other among them (O'Leary et al., 2013; O'Leary et al., 2014; Rathour & Narayanan, 2012a; Rathour & Narayanan, 2014; Srikanth & Narayanan, 2015), implying clear dissociations between different forms of homeostasis.

### 2.3 Baseline versus plasticity profile homeostasis

An important and necessary cynosure in the physiology of encoding systems is their ability to change in a manner that promotes adaptability to the environment. In other words, the ability to undergo plasticity is an important requirement for it to encode or learn newly available information from the environment. Such plasticity has been shown to be ubiquitous, spanning cellular and network structures across almost all regions, and could be triggered by development (Desai, Cudmore, Nelson, & Turrigiano, 2002; Desai, Rutherford, & Turrigiano, 1999; Luo & Flanagan, 2007; Schreiner & Winer, 2007; Turrigiano & Nelson, 2004; White & Fitzpatrick, 2007), by learning processes (Kandel, 2001; Kandel et al., 2014; Kim & Linden, 2007; Lamprecht & LeDoux, 2004; Narayanan & Johnston, 2012; Titley et al., 2017; Zhang & Linden, 2003) or by pathological insults (Beck & Yaari, 2008; Bernard, Shah, & Johnston, 2007; Brager & Johnston, 2014; Grant, 2012; Johnston, Frick, & Poolos, 2016; Kullmann, 2002; Lee & Jan, 2012; Lehmann‐Horn & Jurkat‐Rott, 1999; Lerche et al., 2013; Poolos & Johnston, 2012). A traditional method to study such plasticity mechanisms is to subject neuronal or synaptic structures to specific activity patterns towards understanding the rules for plasticity in specific components. Assessed through such protocols, distinct synapses show signature profiles of plasticity in terms of the strength and direction of synaptic plasticity elicited by specific activity patterns. Additionally, there are also specific sets of nonsynaptic forms of plasticity (in channel densities and properties, for instance) that are concomitant to the synaptic plasticity induced by different activity patterns (Abbott & Nelson, 2000; Abbott & Regehr, 2004; Bi & Poo, 1998; Bliss & Collingridge, 1993; Bliss & Lomo, 1973; Chung et al., 2009; Chung, Qian, Ehlers, Jan, & Jan, 2009; Cooper & Bear, 2012; Dittman et al., 2000; Dudek & Bear, 1992; Fortune & Rose, 2001; Frick, Magee, & Johnston, 2004; Jorntell & Hansel, 2006; Lin, Lujan, Watanabe, Adelman, & Maylie, 2008; Losonczy, Makara, & Magee, 2008; Lujan, Maylie, & Adelman, 2009; Magee & Johnston, 1997; Markram, Lubke, Frotscher, & Sakmann, 1997; Narayanan & Johnston, 2007; Narayanan & Johnston, 2008; Shah, Hammond, & Hoffman, 2010; Sjostrom, Rancz, Roth, & Hausser, 2008). This implies *plasticity profile homeostasis* (Anirudhan & Narayanan, 2015; Mukunda & Narayanan, 2017), where synapses of the same subtype respond similarly to analogous afferent activity, thereby resulting in a subtype‐dependent rule for synaptic plasticity (Larsen & Sjostrom, 2015). In terms of nonsynaptic plasticity, such plasticity profile homeostasis could be generalized to subtypes of cells manifesting specific forms of neuronal plasticity (in intrinsic properties, for instance).

Juxtaposed against the considerable variability in different constitutive components across neurons of the same subtype, and given the critical dissociations between different forms of homeostasis (Section 2.2), it is easy to deduce that the maintenance of baseline homeostasis of a given measurement (say activity or calcium) does not necessarily imply that the system will respond in a similar manner to identical perturbations (Figure 3b). As the direction and strength of change in activity or calcium is a critical determinant of the plasticity profile (Lisman, 1989; Lisman, Schulman, & Cline, 2002; Lisman, Yasuda, & Raghavachari, 2012; Lisman, 2001; Nevian & Sakmann, 2006; Regehr, 2012; Shouval, Bear, & Cooper, 2002; Sjostrom & Nelson, 2002; Sjostrom et al., 2008; Zucker, 1999; Zucker & Regehr, 2002), variable responses to incoming perturbations (physiological or pathophysiological) would translate to very distinct plasticity profiles even in synapses of the same subtype (Anirudhan & Narayanan, 2015; Mukunda & Narayanan, 2017; O'Leary et al., 2013; Srikanth & Narayanan, 2015). Therefore, from the perspective of homeostasis in encoding systems such as the hippocampus, it is not just sufficient to ask if baseline homeostasis of a given measurement is maintained. It is also important to ask if the response of the system to identical perturbations is similar to enable plasticity profile homeostasis. The absence of such plasticity profile homeostasis would result in very different adaptations to identical perturbations even under baseline conditions, resulting in the absence of signature plasticity profiles being associated with specific neurons and synapses. Although there is dissociation between the maintenance of baseline versus plasticity profile homeostasis, studies have demonstrated degeneracy in the maintenance of short‐ and long‐term plasticity profiles. Specifically, these studies have shown that disparate combinations of ion channel conductances and calcium‐handling mechanisms could yield analogous short‐ or long‐term plasticity profiles (Anirudhan & Narayanan, 2015; Mukunda & Narayanan, 2017). Although we dealt with plasticity profile homeostasis and its dissociation from baseline homeostasis, a related phenomenon that involves plasticity of *plasticity profiles* has been defined as metaplasticity (Abraham, 2008; Abraham & Bear, 1996; Abraham & Tate, 1997; Cooper & Bear, 2012; Hulme, Jones, & Abraham, 2013; Sehgal, Song, Ehlers, & Moyer Jr., 2013). Lines of evidence supporting degeneracy in hippocampal metaplasticity and its roles in stable learning will be explored in Section 3.3.

### 2.4 Encoding and homeostasis within the degeneracy framework

The function of learning systems extends beyond simple maintenance of physiological or plasticity homeostasis. The functional goal in these systems is rather contrary to *maintenance* of homeostasis, because encoding or learning of new information demands *alteration* in physiology/behavior through continual *adaptation* in an experience‐/activity‐dependent manner. This presents a paradoxical requirement where components ought to *change* to encode new information, *without* perturbing the overall homeostatic balance of the system. Thus, encoding of a new experience entails a tricky balance between change and homeostasis (James, 1890):

“Plasticity, then, in the wide sense of the word, means the possession of a structure weak enough to yield to an influence, but strong enough not to yield all at once. Each relatively stable phase of equilibrium in such a structure is marked by what we may call a new set of habits.”

From the degeneracy and physiology perspectives, this balance poses several tricky questions that the literature does not present definitive answers to. For instance, could learning systems accomplish this balance between encoding of new information *and* maintenance of homeostasis *within* the framework of degeneracy? In other words, could the plasticity mechanisms that define encoding *and* the homeostatic mechanisms that negate the impact of perturbation *together* be realized through disparate combinations of constitutive components (Narayanan & Johnston, 2012; Nelson & Turrigiano, 2008; Turrigiano, 2007; Turrigiano, 2011; Turrigiano, Abbott, & Marder, 1994; Turrigiano, 1999; Turrigiano & Nelson, 2000)? Would the availability of more routes to achieve encoding or homeostasis be detrimental or be advantageous towards accomplishing these goals together? Would the dissociations between different forms of homeostasis (Section 2.2) and between baseline versus plasticity profile homeostasis (Section 2.3) translate to severe constraints on accomplishing this balance *within* the framework of degeneracy?

Together, there are lines of evidence supporting the formulation that plasticity and homeostasis individually could be achieved through several nonunique routes through disparate combinations of constituent components (Anirudhan & Narayanan, 2015; Mukunda & Narayanan, 2017; Narayanan & Johnston, 2012; Nelson & Turrigiano, 2008; O'Leary et al., 2013; Srikanth & Narayanan, 2015; Turrigiano, 2007; Turrigiano, 2011; Turrigiano et al., 1994; Turrigiano, 1999; Turrigiano & Nelson, 2000). However, the focus on achieving the *conjoined* goals of effectuating changes in response to new information *and* maintaining robust homeostasis in the face of such changes *within* the framework of degeneracy have been conspicuously lacking. Such focus is especially important because of the seemingly contradictory requirements of the two processes, where one necessitates change and the other works to negate any change, resulting in the possibility where there could be detrimental cross‐interference working towards negating each other. Therefore, for the framework of degeneracy to be relevant in learning systems, it is important that future studies assess the twin goals of encoding and homeostasis to be synergistically conjoined rather than treat them as isolated processes that independently achieve their respective goals (Basak & Narayanan, 2018b; Mishra & Narayanan, 2019). Without the recognition of such synergy between encoding and homeostatic systems, assessing the ability of these two processes to avoid cross‐interference becomes intractable.

### 2.5 Curse‐of‐dimensionality or evolutionary robustness

Curse of dimensionality, coined by Bellman (Bellman, 1957), refers to the extreme difficulties encountered with the comprehension or solution to a problem that involves exorbitantly large numbers of input variables, their attributes and possible solutions. In biology in general, and in neuroscience in particular, the dimensions of the parametric space is typically large, making dimensions of the interactional space (the space that covers all forms of interactions spanning all these parameters) even larger. The variability of parametric values even in systems exhibiting similar functions and the perpetual adaptation of these parameters in response to external perturbations (or even baseline turnover towards maintaining homeostasis) make it impossible to localize any biological function to a small subspace of this large interactional space. This, as a consequence of the curse of dimensionality, translates to mathematical and computational intractability of biological systems because of insufficiency of collected data towards providing an accurate answer to questions related to comprehending or assessing the system.

The framework of degeneracy on the other hand suggests that biological systems thrive on this parametric and interactional complexity because it provides the ideal substrate for arriving at disparate structural routes to robust functional similarity. Several strong qualitative and quantitative arguments, based on several lines of evidence spanning different scales of analysis across different biological systems, have been placed in favor of synergistic links between degeneracy, complexity, robustness, evolvability, and adaptation. Therefore, the dimensionality of the parametric and interactional space of biological systems should not be treated as a curse in terms of our inability to analytically track or comprehend the system, but as a fundamental and necessary feature towards achieving the contradictory yet conjoint goals (Section 2.4) of functional robustness (Edelman & Gally, 2001; Kitano, 2007; Marder, 2011; Marder & Goaillard, 2006; Rathour et al., 2016; Rathour & Narayanan, 2012a; Rathour & Narayanan, 2014; Sporns, Tononi, & Edelman, 2000; Stelling et al., 2004; Tononi & Cirelli, 2006; Tononi & Edelman, 1998; Tononi, Edelman, & Sporns, 1998; Wagner, 2005; Wagner, 2008), evolvability (Edelman & Gally, 2001; Wagner, 2008; Whitacre & Bender, 2010; Whitacre, 2010) and adaptation (Albantakis, Hintze, Koch, Adami, & Tononi, 2014; Anirudhan & Narayanan, 2015; Joshi, Tononi, & Koch, 2013; Mukunda & Narayanan, 2017).

Importantly, the recognition of the critical links between complexity, degeneracy and adaptability allows for better design of experimental and analysis techniques for assessing biological systems and their function. Not only do these techniques alleviate the pains of hand tuning in computational models (Prinz, Billimoria, & Marder, 2003), but also recognize the implications for parametric variability to robust functions and the fallacies associated with misinterpretation of results from knockout animals in the face of perpetual biological compensation (Edelman & Gally, 2001; Grashow et al., 2010; Marder, 2011; Marder & Goaillard, 2006; Marder & Taylor, 2011; O'Leary et al., 2014; Taylor et al., 2009; Wagner, 2005). Some classes of techniques developed with the recognition of the strong links between variability, complexity, adaptability, degeneracy and robustness are: (a) the global sensitivity analysis technique (Section 3.2) that employs a stochastic search algorithm spanning a large parametric space and optimizes for *multiple* physiological objectives (Foster et al., 1993; Goldman et al., 2001; Marder, 2011; Marder & Goaillard, 2006; Marder & Taylor, 2011; Prinz et al., 2004; Rathour & Narayanan, 2014); (b) the theoretical and experimental assessment of the links between quantitative complexity measures and robustness with reference to several physiological and pathophysiological attributes (Albantakis et al., 2014; Edelman & Gally, 2001; Joshi et al., 2013; Kitano, 2007; Sarasso et al., 2015; Sporns et al., 2000; Stelling et al., 2004; Tononi & Edelman, 1998; Tononi et al., 1998; Tononi et al., 1996; Tononi et al., 1999; Wagner, 2005; Wagner, 2008; Whitacre & Bender, 2010; Whitacre, 2010); and (c) plasticity models that have accounted for concomitant changes in multiple components (3.6, 3.7) rather than focusing on a one‐to‐one relationship between functional plasticity and one specific component that undergoes changes (Abbott & LeMasson, 1993; Anirudhan & Narayanan, 2015; LeMasson, Marder, & Abbott, 1993; Mukunda & Narayanan, 2017; O'Leary et al., 2013; O'Leary et al., 2014; Siegel et al., 1994; Srikanth & Narayanan, 2015). These analyses have made it abundantly clear that the complexities inherent to biological systems should be considered as substrates for functional robustness through degeneracy (Edelman & Gally, 2001), rather than be viewed from the curse‐of‐dimensionality perspective.

### 2.6 Error correction mechanisms

A critical requirement in a system that is endowed with degeneracy is an error‐correcting feedback mechanism that regulates constituent components in an effort to achieve a specific function. For instance, consider the example where the goal is to achieve calcium homeostasis in a neuron. In this scenario, as the specific regulatory mechanism that is to be triggered is dependent on the current state of the neuron, or more precisely the current levels of calcium, it is important that the regulatory mechanism is geared towards *correcting* the *error* between the target function and the current state (Abbott & LeMasson, 1993; LeMasson et al., 1993; O'Leary et al., 2013; O'Leary et al., 2014; Siegel et al., 1994; Srikanth & Narayanan, 2015). This requires a closed circuit feedback loop that initiates a compensatory mechanism that is driven by the quantitative distance between the target function and the current state. This state‐dependent perpetual error correction becomes especially important in a scenario where distinct regulatory mechanisms govern the different constitute components. With the specific example at hand, let us say the error correcting feedback mechanism regulates ion channel conductances by altering their protein expression through several transcription factors (Srikanth & Narayanan, 2015). In such a scenario, calcium homeostasis could be achieved by recruiting several nonunique sets of these transcription factors. As each of these transcription factors could be coupled to the regulation of distinct combinations of ion channels, calcium homeostasis could be achieved through several nonunique combinations of ion channels.

Within the degeneracy framework, although distinct solutions are possible with weak pairwise correlations between constitutive components, there is a strong synergistic *collective* dependence of these components to achieve a function (Rathour & Narayanan, 2014). Specifically, let us consider two neurons (neurons 1 and 2) with distinct sets of nonunique parametric combinations that yielded very similar function. However, given the nonlinearities of neural systems, it would be infeasible to expect similar function from a third neuron built with one‐half of the parameters taken from neuron 1 and the other half taken from neuron 2. This collective cross‐dependence is an essential component of systems manifesting degeneracy and should be respected by mechanisms that regulate the constitutive components. Returning to specific example under consideration, the specific *ensemble* of the targeted transcription factors and channel conductances are important in terms of which solution is *chosen* within the degeneracy framework. This places strong requirements on the distinct regulatory mechanisms, transcription factors in this case, that they strongly interact with each other rather than acting independent of each other (Srikanth & Narayanan, 2015) in a manner that is *driven* by the error that is being fed back in a state‐dependent temporally precise manner.

These requirements become especially important in an encoding system such as the hippocampus, whose afferent activity is perpetually variable in a behavioral state‐dependent manner, requiring temporally proximal feedback for the continuous maintenance of robust function. A simple solution to account for cross‐interacting regulatory mechanisms is to assume the existence of only one regulatory mechanism that governs all constitutive components (e.g., one transcription factor controls all channels and receptors on a neuron (O'Leary et al., 2014)). However, this might not always be valid or possible or feasible (Srikanth & Narayanan, 2015), especially if the complexity of system is enormous (e.g., coexistence of multiple transcription factors in the hippocampus (Alberini, 2009; Bading, Ginty, & Greenberg, 1993; Dolmetsch, 2003; Lein et al., 2007). In these scenarios, it is important that the error‐sensing and regulatory mechanisms also exhibit degeneracy and are strongly inter‐coupled to each other through cross‐regulatory mechanisms at that scale as well (e.g., multiple calcium sensors accompanied by a network of transcription factors coupled through feedback loops that regulate each other (Cheong, Rhee, Wang, Nemenman, & Levchenko, 2011; Kotaleski & Blackwell, 2010; Losick & Desplan, 2008; Thattai & van Oudenaarden, 2001; Yu et al., 2008)). In summary, the ability to achieve functional robustness through degeneracy in any scale of analysis requires continuous correction of functional deficits, without which it is impossible to adjudge the efficacious accomplishment of a desired goal through a chosen route (which is one among the many possible routes). In a system with enormous complexity, this is typically achieved through an error‐correcting feedback pathway that recruits multiple cross‐interacting regulatory mechanisms towards maintaining collective cross‐dependence of constituent mechanisms (Rathour & Narayanan, 2014; Srikanth & Narayanan, 2015).

## 3 DEGENERACY AT MULTIPLE SCALES IN THE HIPPOCAMPUS

The hippocampus is a brain region that has been shown to be critically involved in spatial representation of the external environment and in several forms of learning and memory (Anderson, Morris, Amaral, Bliss, & O'Keefe, 2007; Eichenbaum, 2012; Hartley, Lever, Burgess, & O'Keefe, 2014; Moser, Kropff, & Moser, 2008; Neves, Cooke, & Bliss, 2008; Scoville & Milner, 1957). As a region that is involved in encoding of new information and one that is part of the medial temporal lobe that is critically sensitive to excitotoxic insults (Bernard et al., 2007; Dam, 1980; de Lanerolle, Kim, Robbins, & Spencer, 1989; Johnston et al., 2016; Sloviter, 1991), it is important that the hippocampal cells maintain some form of activity homeostasis to avoid runaway excitation.

The hippocampus consists of several subtypes of neurons and glia receiving afferent information from tens of thousands of synapses and expressing distinct sets of a wide variety of ligand‐gated receptors and voltage‐gated ion channels, each built through complex structural interactions between a number of main and auxiliary subunits (Lai & Jan, 2006; Migliore & Shepherd, 2002; Nusser, 2009; Nusser, 2012; Vacher, Mohapatra, & Trimmer, 2008; Verkhratsky & Steinhauser, 2000). The regulatory role of glial cells and their constitutive components in synaptic information processing is well established (Allen & Barres, 2005; Allen & Barres, 2009; Araque, 2008; Araque et al., 2014; Araque, Parpura, Sanzgiri, & Haydon, 1999; Ashhad & Narayanan, 2019; Bazargani & Attwell, 2016; Deitmer, McCarthy, Scemes, & Giaume, 2006; Fields & Stevens‐Graham, 2002; Halassa, Fellin, & Haydon, 2007; Halassa & Haydon, 2010; Haydon & Carmignoto, 2006; Pannasch & Rouach, 2013; Pascual et al., 2005; Perea & Araque, 2005; Perea, Navarrete, & Araque, 2009), providing additional structural substrates that could participate in the encoding and homeostasis processes. The basic properties and regulation of these and other membrane and cytoplasmic protein structures, in conjunction with intracellular (including the ER and the trafficking apparatus) and intercellular interaction dynamics (including neuronal synaptic connectivity and the glial syncytium) and morphological characteristics, regulates the intricate balance between encoding and homeostasis within the hippocampal structure. In addition to these, hippocampal structure and function are critically reliant on the afferent and efferent connectivity patterns, the metabolic pathways that drive and interact with the local cellular structures and the several forms of state‐dependent modifications to each of these components. Together, the combinatorial complexity of the constitutive components that define hippocampal function is staggeringly astronomical.

A fundamental question that is of considerable interest to the research community is on how the hippocampus achieves robust function, especially in accomplishing the apparently contradictory goals of adaptive change and homeostasis (Section 2), in the face of such combinatorial complexity that drives its physiology and plasticity. Within the framework of degeneracy, it could be argued that the complexity is an enabler, and not an impediment, towards achieving functional robustness.

Does hippocampal physiology manifest degeneracy at multiple scales, whereby similar hippocampal function could be achieved through disparate structural combinations? In this section, we view hippocampal research spanning the past several decades through the lens of degeneracy and present qualitative and quantitative lines of evidence arguing for the ubiquitous presence of degeneracy spanning multiple scales of hippocampal function. We review lines of evidence showing multiple routes to achieving several critical hippocampal functions, which in some cases have been considered to be lines of evidence that are in apparent contradiction to each other, triggering expansive debates and arguments within the field. We note that while these lines of evidence are broadly consistent with the expression of degeneracy in the hippocampus, they are not outright confirmation that degeneracy is indeed employed by the hippocampus in achieving these functions.

In a manner similar to (Edelman & Gally, 2001), we systematically explore the expression of degeneracy at distinct scales (starting at the molecular scale and moving incrementally to the systems/behavioral scale) of hippocampal function (Figure 1a), with function(s) or physiological measurements assessed within the specified scale of analysis. We postulate that future experiments designed to explore the expression and implications for the prevalence of degeneracy would provide an evolutionarily routed framework to unify the several apparently contradictory routes to achieving the same function as necessity, rather than luxury, towards achieving physiological robustness.

### 3.1 Degeneracy in the properties of channels and receptors

Hippocampal neurons are endowed with myriad voltage and ligand dependent ion channels, with well‐defined gradients in their expression profiles and their properties (Barnard et al., 1998; Dingledine, Borges, Bowie, & Traynelis, 1999; Johnston & Narayanan, 2008; Magee & Cook, 2000; Migliore & Shepherd, 2002; Narayanan & Johnston, 2012; Paoletti, Bellone, & Zhou, 2013; Sieghart & Sperk, 2002). The presence of these channels, with their signature characteristics and expression profiles, has been shown to play critical roles in the physiology (Das et al., 2017; Johnston, Magee, Colbert, & Cristie, 1996; Johnston & Narayanan, 2008; Magee, 2000; Narayanan & Johnston, 2012), plasticity (Frick & Johnston, 2005; Johnston et al., 2003; Remy, Beck, & Yaari, 2010; Shah et al., 2010; Sjostrom et al., 2008) and pathophysiology (Bernard et al., 2007; Brager & Johnston, 2014; Johnston et al., 2016; Kullmann, 2002; Lee & Jan, 2012; Lerche et al., 2013) of hippocampal neurons and their networks. Therefore, it is essential that the biophysical properties and expression profiles of these channels be tightly regulated to ensure functional robustness.

The regulation of targeting, localization and properties of these channels at specific levels, however, is a problem that involves several degrees of combinatorial freedom. The reasons behind this complexity are manifold. First, most of these channels are not protein molecules derived from single genes, but are assembled from several possible pore‐forming and auxiliary subunits, expressed in different stoichiometry (Catterall, 1993; Catterall, 1995; Gurnett & Campbell, 1996; Hille, 2001; Isom, De Jongh, & Catterall, 1994). The presence or absence of a specific pore‐forming or auxiliary subunit and the specific ratios of their expression are important for trafficking, localization and properties of these channels. For instance, *A*‐type K^+^ channels in the hippocampus could be assembled by the main subunits from the Kv1 or Kv4 families and auxiliary subunits from the KChIP and DPP families (Amarillo et al., 2008; Birnbaum et al., 2004; Jerng, Pfaffinger, & Covarrubias, 2004; Kim, Jung, Clemens, Petralia, & Hoffman, 2007; Kim, Wei, & Hoffman, 2005; Sun et al., 2011; Vacher & Trimmer, 2011), whereas auxiliary subunits MiRP1, KCR1 and TRIP8b have been implicated in regulating trafficking and properties of *h* channels assembled with main subunits from the HCN family of proteins. Additionally, the properties of *h* channels, in terms of their voltage‐dependence, their kinetics and modulation by cyclic nucleotides, are critically regulated by the specific isoforms that are expressed in conjunction with the specific stoichiometry of such expression (Biel, Wahl‐Schott, Michalakis, & Zong, 2009; He, Chen, Li, & Hu, 2014; Lewis et al., 2011; Much et al., 2003; Robinson & Siegelbaum, 2003; Santoro et al., 2000; Santoro et al., 2009; Santoro, Wainger, & Siegelbaum, 2004; Ulens & Siegelbaum, 2003; Ulens & Tytgat, 2001; Zolles et al., 2009).

Second, targeting and functional properties of these assembled channels (Trimmer & Rhodes, 2004; Vacher et al., 2008) could be critically modulated by different forms of post‐translational modification (Derkach, Barria, & Soderling, 1999; Derkach, Oh, Guire, & Soderling, 2007; Levitan, 1994; Misonou et al., 2004; Much et al., 2003; Shah et al., 2010; Sjostrom et al., 2008), by local pH (Holzer, 2009), by interaction with intracellular messengers (Armstrong & Bezanilla, 1974) and by lipid composition of the plasma membrane (Levitan & Barrantes, 2012). For instance, trafficking of *A*‐type K^+^ channels is phospho‐regulated in a manner that is dependent on their main and auxiliary subunits (Birnbaum et al., 2004; Hammond, Lin, Sidorov, Wikenheiser, & Hoffman, 2008; Lin, Sun, Kung, Dell'Acqua, & Hoffman, 2011; Lin, Sun, Wikenheiser, Kung, & Hoffman, 2010; Vacher & Trimmer, 2011), and differences between proximal and distal dendritic sodium channels are partly mediated by phosphorylation states of these channels (Gasparini & Magee, 2002).

Third, distinct channels have been demonstrated to have structural interactions with each other, thereby cross regulating the functional properties of each other. For instance, structural interactions between Cav3 and Kv4 channel families are known to regulate neuronal activity through efficient transfer of calcium influx from Cav3 channels to bind onto KChIPs that modulate Kv4 channel function (Anderson et al., 2010). Finally, these channels can undergo activity‐dependent plasticity and neuromodulation (Biel et al., 2009; Cantrell & Catterall, 2001; He et al., 2014; Hoffman & Johnston, 1999; Lee & Dan, 2012; Marder, 2012; Marder et al., 2014; Marder & Thirumalai, 2002; Robinson & Siegelbaum, 2003), which also could result in important changes to their trafficking and functional properties (Section 3.6).

How do these channels maintain specific location‐dependent levels of expression with specific properties despite this staggering complexity that results in their assemblage and specific function? From the description above, it is clear that channels achieve specific properties and localization through multiple structural routes involving several subunits, enzymes associated with post‐translational modification, neuromodulators and their receptors and several signaling cascades (see 3.4, 3.6). This follows the observation that each functional property of the channel, including its localization and targeting, is regulated by multiple mechanisms, each endowed with the ability to bidirectionally modulate the functional property. Therefore, the combinatorial complexity of regulation and the involvement of different structural routes to achieve similar function together provide ample lines of evidence for the expression of degeneracy in achieving specific function for channels and receptors expressed in the hippocampus. In answering the question on how robustness might be achieved, the argument within the framework of degeneracy would be that functional robustness in the assemblage, targeting and function of ion channels is achieved as a *consequence* of the underlying regulatory and interactional complexity.

### 3.2 Degeneracy in neuronal physiological properties

The presence of various ligand and voltage dependent ion channels confers signature neurophysiological properties, such as input resistance, firing rate, frequency selectivity and integration, and propagation of potentials across axonal and dendritic processes, upon different hippocampal neurons (Hutcheon & Yarom, 2000; Johnston et al., 1996; Llinas, 1988). Although there is remarkable variability in these measurements even within a single neuronal subtype (Dougherty, Islam, & Johnston, 2012; Dougherty et al., 2013; Malik, Dougherty, Parikh, Byrne, & Johnston, 2016), different neuronal subtypes within the same subregion have signature electrophysiological characteristics (Anderson P, Anderson et al., 2007; Freund & Buzsaki, 1996; Klausberger & Somogyi, 2008; Spruston, 2008) that are maintained despite the combinatorial complexity of ion channels expressed in these neurons. Additionally, prominent relationships between intrinsic neurophysiological properties and various pathological conditions, including epilepsy and Fragile X mental disorder, have been reported across several neurological disorders (Beck & Yaari, 2008; Bernard et al., 2007; Brager & Johnston, 2014; Johnston et al., 2016; Kullmann, 2002; Lee & Jan, 2012; Lehmann‐Horn & Jurkat‐Rott, 1999; Lerche et al., 2013; Poolos & Johnston, 2012). Thus, from the perspective of maintaining robust physiology and of avoiding pathological excitability conditions, it is essential that neurons maintain their signature electrophysiological characteristics.

It is now recognized across systems that there is no one‐to‐one relationship between neurophysiological properties and the channels that regulate them (2.1, 2.3, Figures 2 and 3). It is established that several channels contribute to the emergence and regulation of a specific physiological property, and the same channel could regulate several physiological properties, resulting in a many‐to‐many mapping between channels and physiological properties. In addition to the example assessing degeneracy in resonance properties (2.1, 2.2, Figures 2 and 3), we could also consider the example of maintaining neuronal firing rates at specific levels. Whereas fast Na^+^ and delayed rectifier K^+^ channels mediate action potential firing in hippocampal neurons, their firing rate profiles are regulated by an array of ion channels including the *A*‐type K^*+*^, HCN, GIRK, *M*‐type K^+^ and SK channels (Adelman, Maylie, & Sah, 2012; Gasparini & DiFrancesco, 1997; Gu, Vervaeke, Hu, & Storm, 2005; Hu, Vervaeke, & Storm, 2007; Kim & Johnston, 2015; Kim et al., 2005; Malik & Johnston, 2017; Migliore et al., 2018; Narayanan & Johnston, 2007; Rathour et al., 2016).

These observations provide specific insights about the relationship between channels and physiological properties (2.1, 2.3; Figure 2 and 3). First, there is degeneracy in the emergence of neurophysiological properties, where disparate combinations of channels could come together to elicit similar functional properties (Basak & Narayanan, 2018b; Das et al., 2017; Drion et al., 2015; Foster et al., 1993; Goldman et al., 2001; Marder, 2011; Marder & Goaillard, 2006; Mishra & Narayanan, 2019; Mittal & Narayanan, 2018; Rathour et al., 2016; Rathour & Narayanan, 2012a; Rathour & Narayanan, 2014; Taylor et al., 2009).

Second, the dependence of different physiological properties on distinct channels is variable even within the same neuronal subtype, and is a function of the variable expression profiles of these channels (Drion et al., 2015; O'Leary et al., 2014; Rathour & Narayanan, 2014; Taylor et al., 2009). For instance, whereas *A*‐type K^+^ channels might contribute maximally to maintaining firing rates at a specific level in one neuron, in another neuron of the same subtype it could be SK channels.

Third, the dependence of different physiological properties in the *same* neuron on distinct channels is differential and variable, where pharmacological blockade of one channel may have a stronger effect on a specific physiological property compared to another (Rathour et al., 2016). As a consequence of these observations, there is a dissociation between robust maintenance of one physiological property and that of another (Srikanth & Narayanan, 2015). Maintenance of only a few physiological properties would not necessarily translate to maintenance of all physiologically relevant properties. All relevant physiological properties ought to be explicitly maintained for overall robustness.

Fourth, hippocampal neurons are endowed with complex dendritic arborization with several well‐defined functional maps expressing along their somatodendritic arbor, making proteostasis, or protein homeostasis (Balch, Morimoto, Dillin, & Kelly, 2008), in these neurons a complex problem (Hanus & Schuman, 2013; Narayanan & Johnston, 2012). Despite the strong structural constraint of maintaining robustness of several tightly coupled location‐dependent functional measurements, it has been demonstrated that it is not essential to maintain individual channels at specific densities or with specific properties for achieving robust functional homeostasis. Instead, several disparate combinations of channel parameters, spanning properties and densities of several channels, could robustly maintain *concomitant* homeostasis of multiple functions across the dendritic arbor (Basak & Narayanan, 2018b; Rathour & Narayanan, 2014). It is however essential to note that dendritic morphology plays a crucial role in regulating intrinsic properties and their location‐dependent characteristics, especially in electrotonically *non*compact hippocampal pyramidal neurons (Dhupia, Rathour, & Narayanan, 2015; Golding, Mickus, Katz, Kath, & Spruston, 2005; Krichmar, Nasuto, Scorcioni, Washington, & Ascoli, 2002; Mainen & Sejnowski, 1996; Narayanan & Chattarji, 2010; Spruston, Jaffe, & Johnston, 1994; Spruston, Jaffe, Williams, & Johnston, 1993), and could contribute to degeneracy in the emergence of single‐neuron physiology.

Finally, depending on the localization profiles and voltage‐dependent properties of different channels they may or may not spatiotemporally interact (Migliore & Migliore, 2012; Mishra & Narayanan, 2015; Rathour & Narayanan, 2012b). For instance, owing to mostly nonoverlapping voltage‐dependence and localization profiles, *M*‐type K^+^ and HCN channels mediate complementary somatodendritic theta filtering in hippocampal neurons (Hu et al., 2009; Narayanan & Johnston, 2007; Narayanan & Johnston, 2008). In contrast, *A*‐type K^+^ and HCN channels strongly overlap both in their voltage‐dependence and localization, resulting in their ability to co‐regulate the same form of resonance in hippocampal pyramidal neurons (Rathour et al., 2016; Rathour & Narayanan, 2012a; Rathour & Narayanan, 2014)

These insights are driven by experimental observations coupled with physiologically relevant computational models that allowed greater flexibility in terms of understanding mechanistic basis, importance of ion channel interactions and the degree of contribution of each channel type in regulating neuronal properties. Multi parametric multi objective stochastic search algorithms are a class of algorithms that has been employed as an extremely effective method to explore cellular‐level degeneracy in a systematic and rigorous manner through global sensitivity analysis (Anirudhan & Narayanan, 2015; Basak & Narayanan, 2018b; Drion et al., 2015; Foster et al., 1993; Goldman et al., 2001; Mishra & Narayanan, 2019; Mittal & Narayanan, 2018; Mukunda & Narayanan, 2017; Rathour & Narayanan, 2012a; Rathour & Narayanan, 2014; Taylor et al., 2009). These algorithms provide a quantitative route to understanding the structure of the global parametric space in any given model, without making explicit assumptions about co‐variation of different parameters test the robustness of the system to parametric variability. In this technique, model neurons generated by uniform random sampling of the global parametric space are tested against experimental statistics of several measurements. Model neurons that satisfy *several* experimental constraints are declared as “valid models". The use of multiple measurements to establish the validity of models is essential because of afore‐mentioned (2.1, 2.3) dissociation between different forms of homeostasis and the differential dependence of different measurements on distinct constitutive components (Figures 2 and 3). It is well recognized in the design principle of these techniques that establishing physiological equivalence of only a partial set of measurements *does not* necessarily ensure that the other measurements which have not been constrained by the validation process are within the physiological ranges (Achard & De Schutter, 2006; Beining, Mongiat, Schwarzacher, Cuntz, & Jedlicka, 2017; Foster et al., 1993; Goldman et al., 2001; Hobbs & Hooper, 2008; Marder, 2011; Marder & Goaillard, 2006; Marder & Taylor, 2011; Prinz et al., 2003; Prinz et al., 2004; Rathour & Narayanan, 2012a; Rathour & Narayanan, 2014; Srikanth & Narayanan, 2015; Taylor et al., 2009; Tobin, Van Hooser, & Calabrese, 2006; Weaver & Wearne, 2008). If such a stochastic search algorithm fails to yield any valid model that satisfies all the physiological objectives, the interpretation should not be that the specified model configuration is incapable of achieving all objectives. This is because the stochastic search does not *entirely* span the global parametric space, thereby allowing for the possibility that valid solutions could exist within the unexamined regions of this parametric space.

Once the validity of a (typically small) subset of models through multiple physiological constraints is established, the approach has been employed to explore degeneracy by assessing pairwise and cross‐dependencies across different parameters. Pairwise correlations across valid model parametric values are typically employed to explore such dependencies, where a strong correlation between any two parameters is interpreted as a pointer to potential co‐regulation of biological mechanisms defining these parameters (Anirudhan & Narayanan, 2015; Foster et al., 1993; Goldman et al., 2001; Mukunda & Narayanan, 2017; Rathour & Narayanan, 2012a; Rathour & Narayanan, 2014; Taylor et al., 2009). These analyses also provide insights about how critically specific parameters should be regulated to achieve the *multiple* objectives imposed by the validation criteria. Importantly, these algorithms provide a quantitative route to finding the relative sensitivities of different measurements to each channel that contributed to the emergence of robust functionality spanning multiple measurements. It is recognized that the dependence of measurements on individual channels would be variable given that different model neurons are endowed with considerable variability in each channel conductance. However, it is known that the average dependence of a given measurement (say resonance frequency) is higher for one specific channel (say HCN channels), *relative* to the other channels expressed in the system. Different methodologies have been proposed to assess these relative contributions and have been effectively employed to understand the differential and variable dependencies of different measurements on each underlying channel (Basak & Narayanan, 2018b; Mittal & Narayanan, 2018; O'Leary et al., 2014; Rathour & Narayanan, 2014; Taylor et al., 2009).

Together, through a confluence of electrophysiological and computational techniques that assessed variability and homeostasis in neuronal and channel properties, the expression of degeneracy in the emergence of single neuron physiology is well established across several systems, including the mammalian hippocampus. It is clear that disparate combination of morphological and channel parameters could robustly yield analogous single neuron physiology, despite being constrained by *multiple* measurements that span the entire somatodendritic arbor of the *same* neuron.

### 3.3 Degeneracy in calcium regulation and in the induction of synaptic plasticity

Whereas the ability to maintain baseline physiological measurements at specific levels is important from the homeostasis perspective, the ability to alter responses (through changes in parameters) towards achieving a specific target is important from the perspective of learning or encoding. This ability to undergo long‐term plasticity is absolutely critical in an encoding system. One of the most well studied forms of long‐term plasticity in hippocampal neurons is plasticity in synaptic structures. There are several lines of evidence for degeneracy in the induction, expression, and maintenance of long‐term synaptic plasticity and the mechanisms that are associated with each of these distinct phases of synaptic plasticity. As long‐term synaptic plasticity is relatively well studied, we will first outline these lines of evidence from the synaptic plasticity perspective and then switch to the implications for *concomitant* nonsynaptic plasticity that typically accompanies synaptic plasticity.

**Figure 4:**
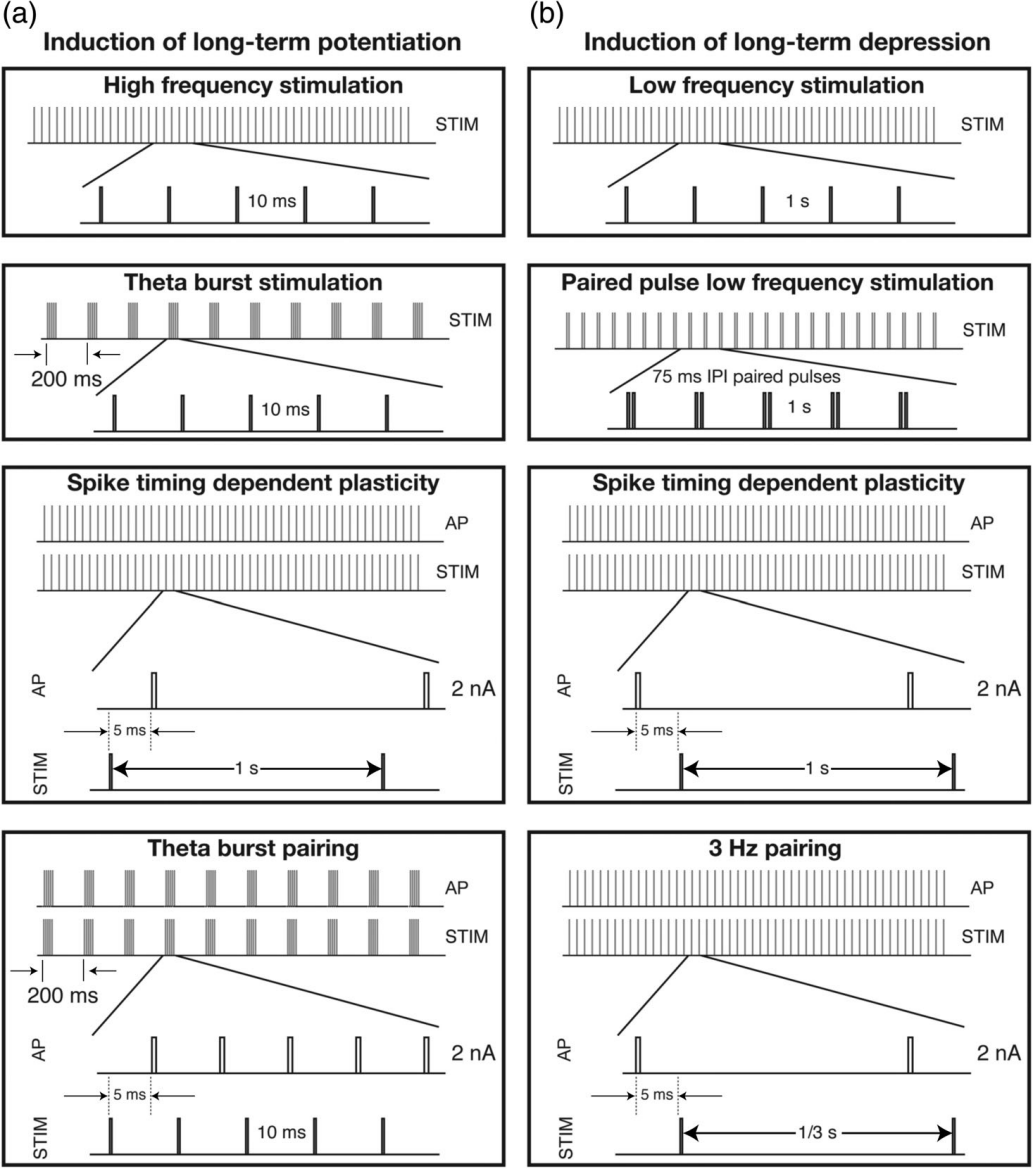
Disparate activity‐dependent protocols have been employed for the induction of long‐term potentiation or depression in hippocampal synapses. (a, b) Disparate activity‐dependent induction protocols yield long‐term potentiation (a) or depression (b) in Schaffer collateral synapses connecting CA3 pyramidal neurons to CA1 pyramidal neurons. Individual panels depict cartoon illustrations of induction protocols employed in previous studies (Bi & Poo, 1998; Christie, Magee, & Johnston, 1996; Dudek & Bear, 1992; Huber, Kayser, & Bear, 2000; Larson, Wong, & Lynch, 1986; Magee & Johnston, 1997). A subset of similar or additional protocols that have been employed in the induction of potentiation or depression in hippocampal synapses may be found here: (Basu et al., 2016; Bittner et al., 2015; Bittner, Milstein, Grienberger, Romani, & Magee, 2017; Bliss & Collingridge, 1993; Bliss & Gardner‐Medwin, 1973; Bliss & Lomo, 1973; Chevaleyre, Takahashi, & Castillo, 2006; Christie, Kerr, & Abraham, 1994; Dan & Poo, 2006; Dudek & Bear, 1992; Dudek & Bear, 1993; Dudman, Tsay, & Siegelbaum, 2007; Larkman & Jack, 1995; Lynch, Larson, Kelso, Barrionuevo, & Schottler, 1983; Lynch, Dunwiddie, & Gribkoff, 1977; Malenka, Lancaster, & Zucker, 1992; Mulkey & Malenka, 1992; Raymond, 2007; Regehr, Carey, & Best, 2009; Staubli & Lynch, 1990; Takahashi & Magee, 2009). AP, action potential; IPI, inter pulse interval; STIM, stimulation leading to postsynaptic potentials

A popular methodology to study long‐term synaptic plasticity in neurons within the hippocampus and other brain structures is the use of specific induction protocols that result in synaptic plasticity. These induction protocols are activity‐dependent, and are typically induced by combinations of presynaptic stimulation and/or postsynaptic current injection. There are also several chemical protocols for inducing synaptic plasticity, say through depolarization induced through elevated levels of extracellular potassium or potassium channel blockers (Hanse & Gustafsson, 1994; Huang & Malenka, 1993; Huber, Mauk, & Kelly, 1995; Lin et al., 2008; Otmakhov et al., 2004; Roth‐Alpermann, Morris, Korte, & Bonhoeffer, 2006). These protocols are critically tied to the specific synaptic structures that are studied and show signature profiles across synaptic structures of similar subtypes (Abbott & Nelson, 2000). The protocols required for induction of synaptic plasticity are not unique. Several disparate protocols with very distinct combinations of presynaptic stimulation and/or postsynaptic current injection (Figure 4) have been shown to elicit long‐term potentiation (LTP) or long‐term depression (LTD). The cellular mechanisms required for inducing LTP are also very different across these protocols, with differences sometimes manifesting even within a single protocol for synapses at two different locations on the same neuron. For instance, with the theta burst protocol for inducing LTP (Figure 4a), proximal synaptic LTP requires pairing with backpropagating action potentials, but distal synapses recruit dendritic spikes and do not require backpropagating action potentials (Golding, Staff, & Spruston, 2002; Kim, Hsu, Cembrowski, Mensh, & Spruston, 2015; Magee & Johnston, 1997).

The ability of multiple activity protocols (Figure 4) to elicit similar levels of synaptic plasticity might be an example of multiple realizability, but it could be argued that this does not constitute an instance of degeneracy, which requires that disparate *structural* components elicit similar function. To address this argument, we refer to established answers for one of the fundamental questions on synaptic plasticity: What is the mechanistic basis for these induction protocols to elicit synaptic plasticity? The influx of calcium into the cytosol is considered as the first step that results in the induction of LTP or LTD (Lynch et al., 1983; Malenka et al., 1992; Mulkey & Malenka, 1992). Quantitatively, there have been suggestions for the amplitude, spread and kinetics of cytosolic calcium elevation to be specific attributes that translate to the strength and direction of plasticity (Larkman & Jack, 1995; Lisman, 1989; Lisman, 2001; Shouval et al., 2002). From this perspective, it may be argued that disparate protocols for inducing LTP (or LTD) result in similar amplitude, spread and kinetics of calcium elevation, thereby resulting in similar strength of LTP (or LTD). With calcium elevation established as a mechanistic basis for the induction of synaptic plasticity, the question of degeneracy here should now focus on the structural basis for eliciting similar elevation in cytosolic calcium.

**Figure 5:**
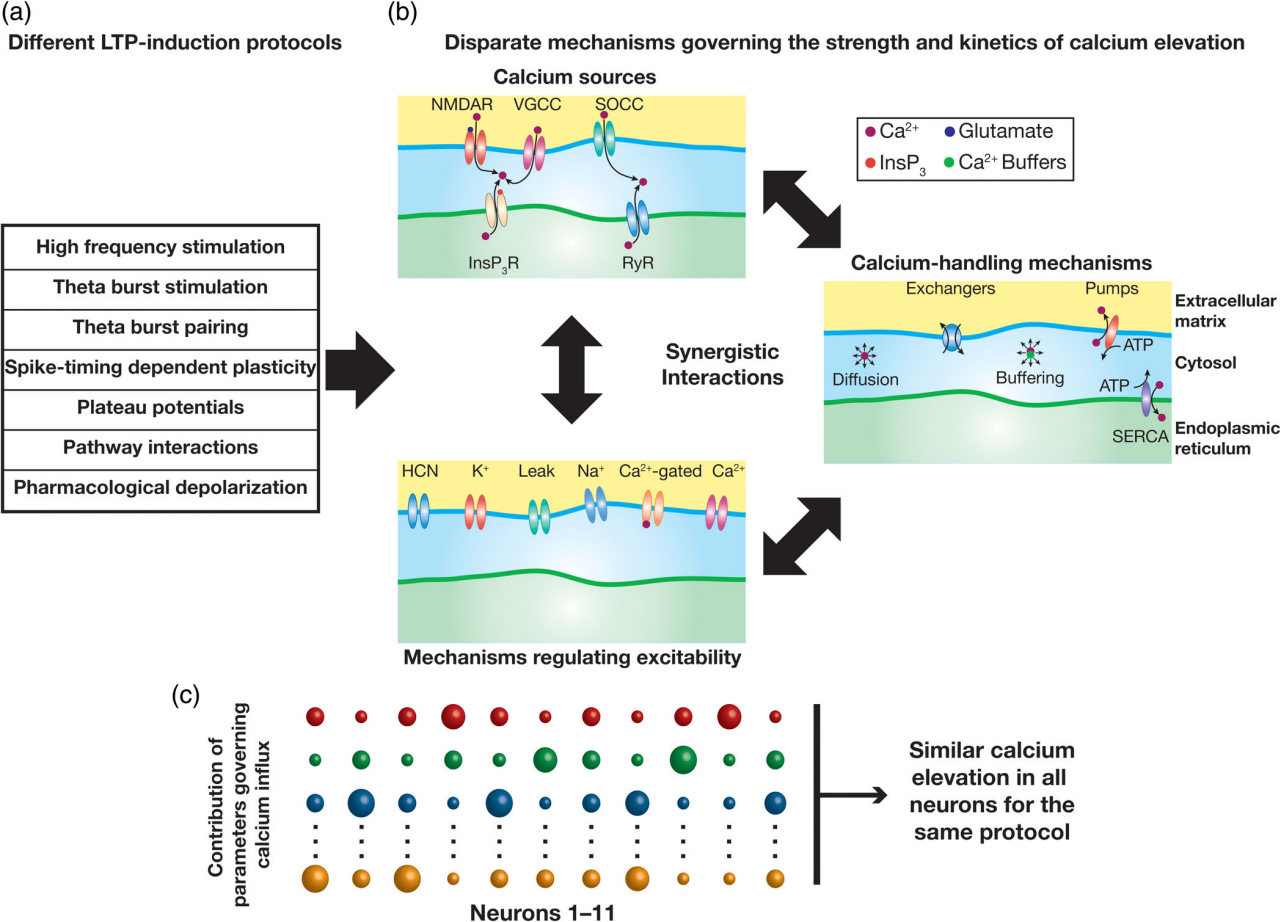
Disparate cellular and molecular mechanisms govern the strength and kinetics of cytosolic calcium influx. (a) Different protocols have been employed for the induction of LTP in hippocampal synapses. Whereas references for the first four of these protocols are provided in Figure 4, the last three are derived from protocols in these references (Basu et al., 2016; Bittner et al., 2015; Bittner et al., 2017; Dudman et al., 2007; Hanse & Gustafsson, 1994; Huang & Malenka, 1993; Huber et al., 1995; Lin et al., 2008; Otmakhov et al., 2004; Roth‐Alpermann et al., 2006; Takahashi & Magee, 2009). (b) Protocols shown in (a) typically elicit postsynaptic calcium influx through synergistic interactions between disparate constitutive components. Although only postsynaptic components are depicted here, it should be noted that presynaptic components, including excitability‐, calcium‐, and release‐regulating mechanisms, also would control the postsynaptic calcium influx through regulation of release dynamics and short‐term plasticity. Additionally induction could also be presynaptic. (c) In different neurons, the contribution of different components to achieve similar strength and kinetics of cytosolic calcium influx could be variable. The size of each sphere scales with the quantum of contribution of a given component to cytosolic calcium influx in a given neuron (11 neurons are depicted). Cartoon representations depicted here are drawn from conclusions arrived in previous studies (Anirudhan & Narayanan, 2015; Mukunda & Narayanan, 2017) [Color figure can be viewed at wileyonlinelibrary.com]

The mechanisms that govern the strength, spread and kinetics of neuronal calcium are well studied (Augustine, Santamaria, & Tanaka, 2003; Berridge, 1998; Berridge, 2002; Berridge, 2006; Berridge, Lipp, & Bootman, 2000; Frick, Magee, Koester, Migliore, & Johnston, 2003; Higley & Sabatini, 2012; Jaffe et al., 1992; Miyakawa et al., 1992; Rizzuto & Pozzan, 2006; Ross, 2012; Sabatini, Oertner, & Svoboda, 2002; Yasuda et al., 2004). Briefly, synergistic interactions between three prominent sets of mechanisms (Figure 5) regulate cytosolic calcium levels, especially from the perspective of induction of synaptic plasticity. First, the disparate structural components through which calcium ions flow into the cytosol either from the extracellular matrix or from the endoplasmic reticulum (ER). These are typically receptors or channels expressed on the plasma membrane or the ER membrane. The second set is built of disparate mechanisms that alter postsynaptic excitability, which mediates the conversion from synaptic current to synaptic voltage responses. Changes in excitability modulate voltage‐levels, which in turn alter calcium influx through voltage‐sensitive synaptic receptors or voltage‐gated calcium channels. Finally, the expression of voltage‐independent calcium‐handling mechanisms such as pumps, exchangers and buffers limit the spatiotemporal spread of calcium thereby maintaining specificity of signaling, apart from regulating the strength and kinetics of calcium influx. Thus, there are disparate mechanisms that regulate calcium influx, and nonunique combinations of these mechanisms could yield similar strength and kinetics of calcium influx in response to different induction protocols.

Importantly, electrophysiological recordings coupled with pharmacological treatments provide strong lines of evidence that induction of synaptic plasticity could indeed be mediated and regulated by these distinct components. Specifically, there are strong lines of evidence that the induction of bidirectional synaptic plasticity in the hippocampus is mediated by different calcium sources, with certain protocols requiring synergistic activation of multiple calcium sources (Brager & Johnston, 2007; Christie et al., 1996; Golding et al., 2002; Huber et al., 1995; Nishiyama, Hong, Mikoshiba, Poo, & Kato, 2000; Raymond, 2007). These studies show that plasticity induction is dependent on influx of calcium through NMDA receptors (Christie et al., 1996; Collingridge & Bliss, 1987; Collingridge, Kehl, & McLennan, 1983; Morris, Anderson, Lynch, & Baudry, 1986; Mulkey & Malenka, 1992; Nishiyama et al., 2000; Tsien, Huerta, & Tonegawa, 1996; Wang, Xu, Wu, Duan, & Poo, 2003), voltage‐gated calcium channels (Brager & Johnston, 2007; Christie et al., 1996; Christie, Schexnayder, & Johnston, 1997; Johnston, Williams, Jaffe, & Gray, 1992; Moosmang et al., 2005; Nicholson & Kullmann, 2017; Wang et al., 2003), store‐operated calcium channels (Baba et al., 2003; Garcia‐Alvarez et al., 2015; Majewski & Kuznicki, 2015; Majewski et al., 2017; Prakriya & Lewis, 2015) and receptors on the ER activated by metabotropic receptors on the plasma membrane (Huber et al., 2000; Jedlicka & Deller, 2017; Nishiyama et al., 2000; Padamsey, Foster, & Emptage, 2019; Verkhratsky, 2002). Additionally, voltage‐gated channels and their auxiliary subunits (Anirudhan & Narayanan, 2015; Brager, Lewis, Chetkovich, & Johnston, 2013; Chen et al., 2006; Chung, Ge, et al., 2009; Chung, Qian, et al., 2009; Johnston et al., 2003; Jung, Kim, & Hoffman, 2008; Kim et al., 2007; Lin et al., 2008; Lujan et al., 2009; Malik & Johnston, 2017; Nolan et al., 2004; Sehgal et al., 2013; Shah et al., 2010; Watanabe, Hoffman, Migliore, & Johnston, 2002) have also been shown to critically regulate the strength and direction of synaptic plasticity. It is important to note that the contribution of *a specific* calcium source to plasticity induction depends on several factors, including the specific protocol employed for inducing plasticity, the somatodendritic location of the synapse and the heterogeneities (of calcium sources, calcium regulators, ion channels, etc.) that are known to express across individual neurons of the same subtype (Figure 5b).

In addition, these distinct structural components that act as calcium sources that contribute to plasticity induction are intricately coupled both structurally as well as functionally, resulting in synergistic interactions between these components. There are established structural interactions between these disparate structural components, with significant cross dependencies. Established interactions include those between metabotropic glutamate receptors, NMDA receptors and inositol trisphosphate receptors (Naisbitt et al., 1999; Tu et al., 1999; Tu et al., 1998; Xiao, Tu, & Worley, 2000), and between store‐operated and voltage‐gated calcium channels (Dittmer, Wild, Dell'Acqua, & Sather, 2017; Heine, Heck, Ciuraszkiewicz, & Bikbaev, 2019; Park, Shcheglovitov, & Dolmetsch, 2010; Wang et al., 2010). Examples for functional coupling include cross‐regulation through calcium, in case of the regulation of inositol trisphosphate receptors (Bezprozvanny, Watras, & Ehrlich, 1991; Choe & Ehrlich, 2006; Finch, Turner, & Goldin, 1991) and calcium‐dependent inactivation of voltage‐gated calcium channels (Budde, Meuth, & Pape, 2002). Together, several structural components that mediate or modulate calcium influx into the cytosol have been demonstrated as critical regulators of the induction of synaptic plasticity, both from the qualitative perspective of expression of plasticity and the quantitative perspective of the specific levels of plasticity attained with an induction protocol.

Finally, deriving from distinctions that we made in Section 2.2 about qualitative and quantitative degeneracy, and consistent with the resonance frequency example that we had presented in Figure 2, it is useful to ask if distinct structural components could combine together to elicit *quantitatively* similar plasticity profiles. In this context, computational modeling has demonstrated that similar synaptic plasticity profiles could be achieved through disparate combinations of channels and receptors (Anirudhan & Narayanan, 2015; Ashhad & Narayanan, 2013; Narayanan & Johnston, 2010; Shouval et al., 2002) and is critically dependent on the state of the synapse (Migliore, De Simone, & Migliore, 2015). In conjunction with the experimental studies reviewed above, these provide very strong lines of evidence for degeneracy in the induction of synaptic plasticity, where similar levels of calcium influx and analogous synaptic plasticity could be achieved through disparate combinations of parameters that synergistically regulate calcium influx (Figure 4b). Qualitatively however, again consistent with the resonance frequency example that we had presented in Figure 2, it is clear that there are components that play an essential and mediating role, and components that play a modulatory role in plasticity induction (Sanes & Lichtman, 1999). For instance, the distinct calcium sources could be considered as essential and mediating roles whereas components that regulate neuronal excitability or excitation‐inhibition balance might be assigned modulatory roles in plasticity induction. The specific definitions of mediatory and modulatory roles, however, would rely on several factors including the specific protocol employed for (or behavioral activity pattern observed in vivo that resulted in) plasticity induction, the synaptic location along the somatodendritic axis, the state of the neuron and the impact of biological heterogeneities.

### 3.4 Degeneracy in signaling cascades that regulate synaptic plasticity

What follows calcium elevation in the process of inducing synaptic plasticity? Once specific strengths and kinetics of calcium influx are achieved as a consequence of induction protocols activating the several disparate mechanisms, is the route to the expression of synaptic plasticity unique? Could multiple mechanisms be activated in response to similar elevations of cytosolic calcium towards achieving specific levels of synaptic plasticity? In other words, is there degeneracy in terms of distinct pathways involving different constitutive components that could link the induction of synaptic plasticity to its expression?

**Figure 6:**
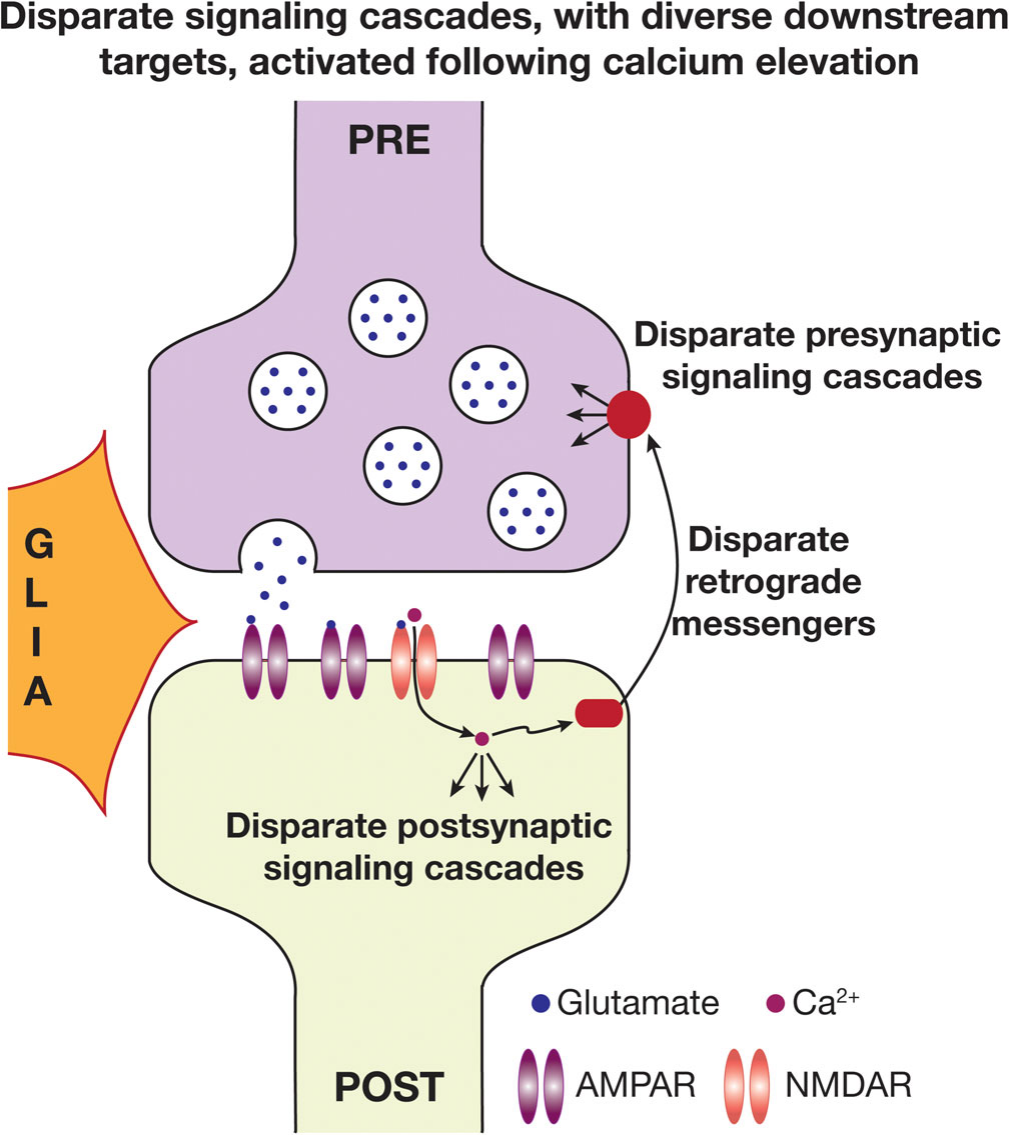
Disparate signaling cascades with diverse downstream targets are activated following postsynaptic calcium elevation. Depicted is a tripartite synapse that includes a presynaptic terminal, a postsynaptic structure and a glial cell. Following the influx of calcium through disparate sources (see Figure 5; shown here is only N‐Methyl‐D‐Aspartate Receptor (NMDAR) for simplicity), several pre‐ and post‐synaptic signaling cascades could be activated with very different downstream targets. Retrograde messengers are responsible for transmitting information about postsynaptic calcium elevation to the presynaptic terminal. Illustration incorporates conclusions from previous studies (Bhalla, 2014; Bhalla & Iyengar, 1999; Kotaleski & Blackwell, 2010; Manninen, Hituri, Kotaleski, Blackwell, & Linne, 2010; Regehr, 2012; Regehr et al., 2009) [Color figure can be viewed at wileyonlinelibrary.com]

The large body of literature on the signaling cascades involved in synaptic plasticity has presented several lines of evidence that there are several signaling routes, contributing synergistically or differentially, to achieving the translation from the induction of synaptic plasticity to its expression (Figure 6). Specifically, there is evidence that there are several biochemical species that control synaptic efficacy through a complex network of spatiotemporally interacting signaling cascades (Bhalla, 2014; Bhalla & Iyengar, 1999; Derkach et al., 2007; Kennedy, 2000; Kennedy, Beale, Carlisle, & Washburn, 2005; Kholodenko, 2006; Kotaleski & Blackwell, 2010; Larkman & Jack, 1995; Ma'ayan et al., 2005; Manninen et al., 2010; Neves & Iyengar, 2009; Neves et al., 2008; Regehr et al., 2009; Weng et al., 1999). It is also clear that the dominance of any specific cascade that determines the strength and direction of plasticity is dependent on synaptic state (Migliore et al., 2015), the protocol employed (Kandel et al., 2014; Mayford et al., 2012) and on the spatiotemporal dynamics of changes in the postsynaptic calcium concentration (Basak & Narayanan, 2018a; Berridge, 1998; Korte & Schmitz, 2016; Lisman, 1989; Lisman, 2001; Parekh, 2008; Rizzuto & Pozzan, 2006).

The biochemical signaling diversity involved in synaptic plasticity spans both the pre‐ and post‐synaptic sides. The signaling cascades involved in the translation of induction to expression include several enzymes that mediate posttranslational modification of disparate protein substrates, protein synthesis regulators, retrograde messengers, protein trafficking regulators and mechanisms mediating structural plasticity. As a specific example, with reference to the diversity of enzymes that are involved in post‐translational modifications resulting in the expression of synaptic plasticity, it has been shown that different protocols for inducing LTP in the Schaffer collateral synapses projecting to CA1 are differentially dependent on different kinases (Kandel, 2001; Kandel et al., 2014; Manninen et al., 2010; Mayford et al., 2012; Raymond, 2007; Soderling & Derkach, 2000). Example kinases are the calcium‐calmodulin kinase II, CaMKII (Lisman et al., 2002; Lisman et al., 2012; Malinow, Schulman, & Tsien, 1989; Ouyang, Kantor, Harris, Schuman, & Kennedy, 1997; Ouyang, Rosenstein, Kreiman, Schuman, & Kennedy, 1999), protein kinase A, PKA (Frey, Huang, & Kandel, 1993; Lin et al., 2008; Otmakhova, Otmakhov, Mortenson, & Lisman, 2000; Rosenkranz, Frick, & Johnston, 2009; Woo, Duffy, Abel, & Nguyen, 2003) and mitogen associated protein kinase, MAPK (English & Sweatt, 1997; Rosenkranz et al., 2009), which could be activated with the same or different LTP protocols. For instance, the theta‐burst pairing protocol activates all of CaMKII, MAPK and PKA (Fan et al., 2005; Lin et al., 2008; Rosenkranz et al., 2009), with very different target substrates involving different channels and receptors (see Section 3.6). Additionally the expression of synaptic plasticity, or the substrate for altered synaptic efficacy, could be dependent on several factors (Section 3.5), each of which could undergo distinct plasticity with reference to the same activity protocols (Section 3.6). Together, the possible combinations of mechanisms that could mediate the translation of plasticity induction protocol to plasticity expression, even for a single synaptic subtype, are numerous. There are also lines of evidence that similar strength and direction of synaptic plasticity could be achieved through the activation of disparate combinations of these mechanisms, providing evidence for the manifestation of degeneracy in the signaling cascades that mediate the transition from plasticity induction to expression.

### 3.5 Degeneracy in the expression of synaptic plasticity

The above analyses present lines of evidence that hippocampal neurons exhibit degeneracy with reference to the induction of synaptic plasticity and in terms of the mechanisms that mediate the transition from induction to expression. Do these mechanisms act in concert to alter a single target to effectuate the expression of synaptic plasticity? Or are there multiple targets that could be altered to achieve similar strength and direction of synaptic plasticity in a specific synapse?

From the very first study that demonstrated LTP, it has been clear that the protocols employed for inducing synaptic plasticity can recruit different structural components (Bliss & Lomo, 1973):

“The results suggest that two independent mechanisms are responsible for long‐lasting potentiation: (a) an increase in the efficiency of synaptic transmission at the perforant path synapses; (b) an increase in the excitability of the granule cell population.”

**Figure 7:**
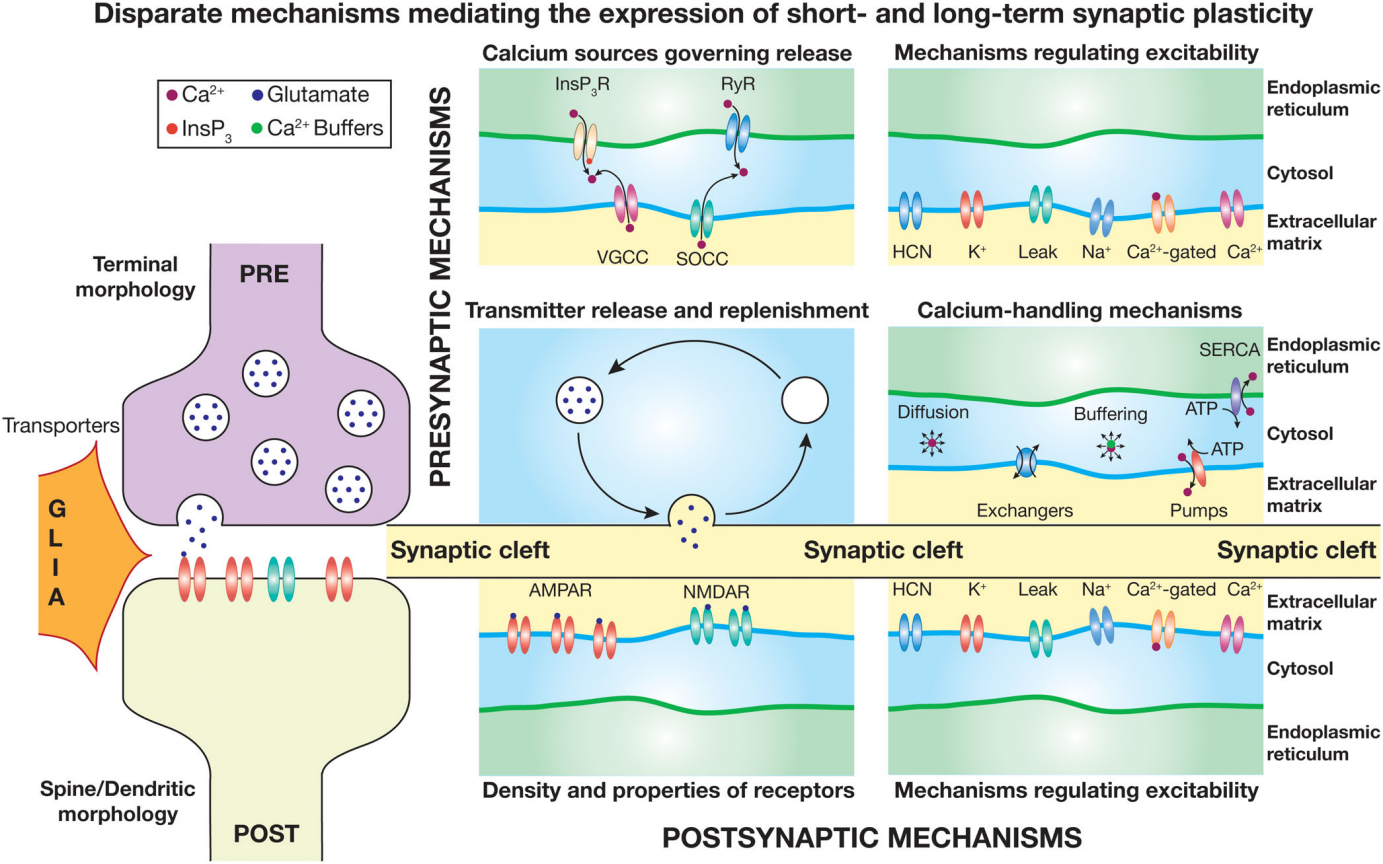
Disparate mechanisms mediate the expression of short‐ and long‐term synaptic plasticity. (Left) Depicted is a tripartite synapse that includes a presynaptic terminal, a postsynaptic structure and a glial cell. (Right) Several pre‐ and post‐synaptic mechanisms regulate synaptic strength, and independent or concomitant long‐term changes in any of these components would result in the expression synaptic plasticity. Plasticity is known to potentially span all these components and more (Kim & Linden, 2007) [Color figure can be viewed at wileyonlinelibrary.com]

Several studies that followed up on this landmark study have now clearly shown that there are disparate routes to achieving synaptic plasticity, even with very similar strength and the same direction of plasticity (Figure 7). It is now well established that the expression of synaptic plasticity could recruit mechanisms spanning pre‐ and post‐synaptic components, including channels/receptors, morphological features and cytoplasmic constituents on either side (Figure 7). In other words, different combinations of changes in presynaptic channels/receptors, release mechanisms and postsynaptic channels/receptors could mediate the expression of synaptic plasticity.

The framework of degeneracy provides an ideal way to reconcile the thorny debates regarding pre‐ and post‐synaptic mechanisms that could mediate synaptic plasticity. Specifically, within this framework, pre‐ and post‐synaptic components would be considered simply as *a subset* (see Section 3.6) of the broad repertoire of mechanisms that are available to the neural system to alter towards achieving a specific level of synaptic plasticity or accomplishing an encoding task. Disparate combinations of these components could synergistically contribute to the expression of specific levels of plasticity, at times even with temporal differences in the expression of plasticity in different components. The specific combination of changes that are recruited to mediate plasticity for a chosen protocol or for a given behavioral task would then be state‐dependent, critically reliant on the specific calcium sources (Section 3.3) and signaling cascades (Section 3.4) that were recruited in response to the induction protocol or a behavioral task. In addition to these neuronal components, glial cells, through several mechanisms including gliotransmission or transmitter reuptake and recycling mechanisms, have also been shown to play a critical role in synaptic plasticity (Araque et al., 2014; Ashhad & Narayanan, 2016; Ashhad & Narayanan, 2019; Halassa et al., 2007; Haydon & Carmignoto, 2006; Henneberger, Papouin, Oliet, & Rusakov, 2010; Pannasch & Rouach, 2013; Perea & Araque, 2007; Perea et al., 2016; Zorec et al., 2012), thereby adding another layer of parameters and another set of interactional complexity to the mechanistic basis for synaptic plasticity.

This combinatorial complexity of parameters and associated interactions provide a strong foundation for degeneracy in the emergence of not just the induction and expression of long‐term plasticity, but also in the emergence of short‐term synaptic plasticity. Specifically, several of the components involved in the induction and expression of long‐term plasticity have also been shown to play critical roles in short‐term forms of plasticity such as paired pulse facilitation, and on the synaptic filters that they mediate (Atwood, Lovinger, & Mathur, 2014; Bouchard, Pattarini, & Geiger, 2003; De Pitta, Volman, Berry, & Ben‐Jacob, 2011; Dittman et al., 2000; Emptage, Reid, & Fine, 2001; Fioravante & Regehr, 2011; Fortune & Rose, 2001; Regehr, 2012; Siegelbaum, 2000; Zucker, 1989; Zucker, 1999; Zucker & Regehr, 2002). These observations, in conjunction with quantitative computational models have led to the suggestion for the manifestation of degeneracy in the emergence of short‐term plasticity profiles and associated synaptic filters (Mukunda & Narayanan, 2017). Specifically, it has been demonstrated that analogous synaptic filters emerge from disparate combinations of presynaptic parameters (Mukunda & Narayanan, 2017). Together, these observations provide clear lines of evidence for the manifestation of degeneracy in short‐ and long‐term forms of synaptic plasticity in the hippocampus.

### 3.6 Degeneracy in the induction and expression of nonsynaptic plasticity

**Figure 8:**
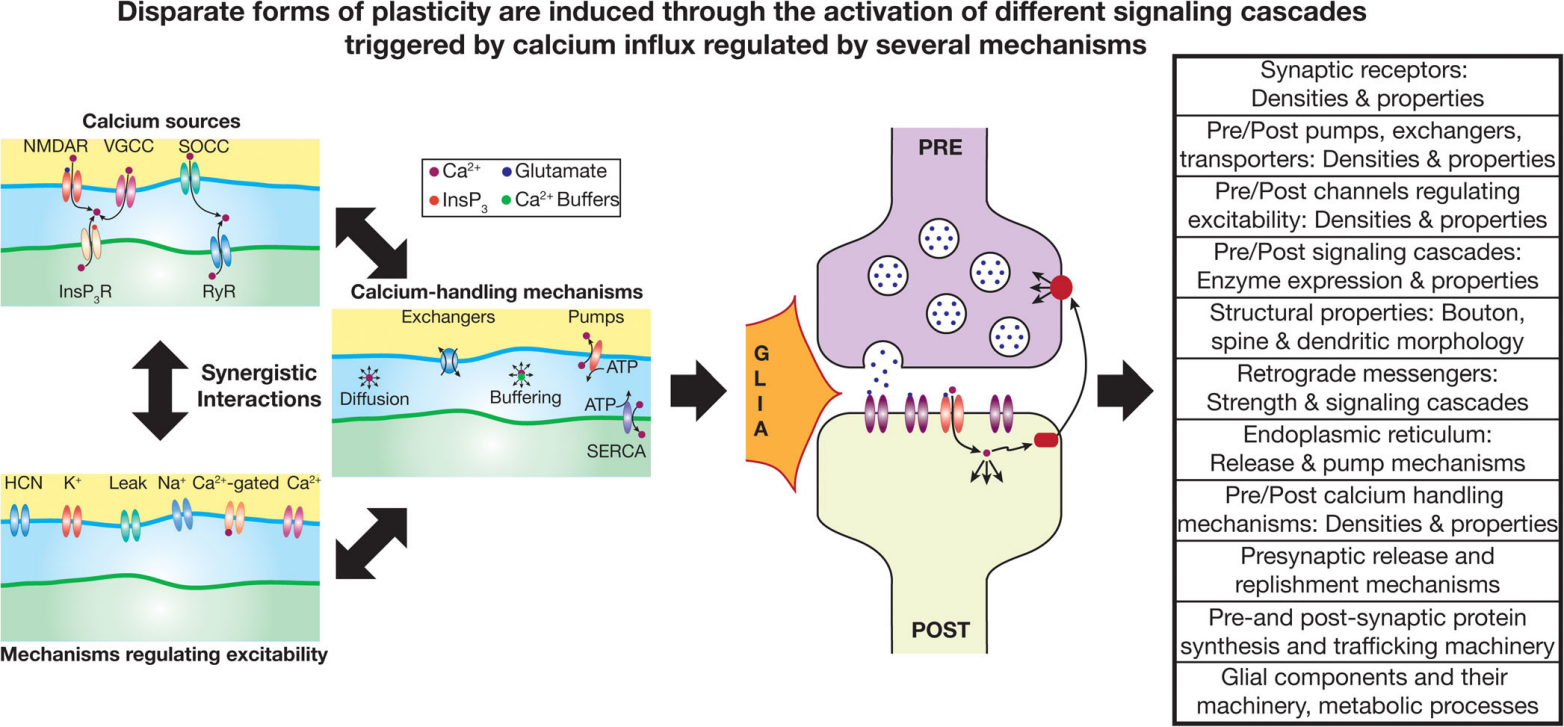
Disparate forms of synaptic and nonsynaptic plasticity are induced through the activation of different signaling cascades triggered by calcium influx regulated by several mechanisms, resulting in multiscale degeneracy in plasticity induction through expression. **(**Left) Synergistic interactions between several components results in cytosolic calcium influx following plasticity induction through activity protocols or behavioral experience of pathological insults. (Center) Disparate signaling cascades with diverse downstream targets are activated following postsynaptic calcium elevation. (Right) The activation of signaling cascades and their impact on their targets are not just limited to synaptic components, but span a large span of neuronal and network components. Several forms of synaptic and nonsynaptic plasticity express concomitantly in response to the same protocols or perturbations (Beck & Yaari, 2008; Johnston et al., 2016; Kim & Linden, 2007; Narayanan & Johnston, 2012) [Color figure can be viewed at wileyonlinelibrary.com]

It is now widely acknowledged that plasticity protocols and learning paradigms that were once assumed to exclusively recruit or induce synaptic plasticity also induce plasticity in other components (Figure 8), in a manner that could either be localized or global. Similar to the study of synaptic plasticity, specific activity protocols (most of which are similar, if not identical, to synaptic plasticity protocols) are employed to assess plasticity in other protein molecules and structural changes. Plasticity in voltage‐gated ion channels and other neuronal components that result in changes to neuronal intrinsic properties have been dubbed as *intrinsic plasticity*, and is known to occur in the hippocampus with reference to most activity‐dependent protocols employed for inducing synaptic plasticity (Brager & Johnston, 2007; Chung, Ge, et al., 2009; Chung, Qian, et al., 2009; Fan et al., 2005; Frick & Johnston, 2005; Frick et al., 2004; Johnston et al., 2003; Johnston & Narayanan, 2008; Kim & Linden, 2007; Lin et al., 2008; Losonczy et al., 2008; Magee, 2000; Mozzachiodi & Byrne, 2010; Narayanan & Johnston, 2007; Narayanan & Johnston, 2008; Narayanan & Johnston, 2012; Nelson & Turrigiano, 2008; Remy et al., 2010; Sjostrom et al., 2008; Spruston, 2008; Wang et al., 2003; Zhang & Linden, 2003). Although it is generally assumed that intrinsic plasticity refers only to global changes in intrinsic *excitability*, it is important to recognize that intrinsic plasticity encompasses *all* intrinsic properties that are mediated by neuronal constitutive components (Llinas, 1988; Marder, 2011; Marder, Abbott, Turrigiano, Liu, & Golowasch, 1996; Marder & Goaillard, 2006), including neuronal spectral selectivity conferred by specific sets of ion channels (Das et al., 2017; Hutcheon & Yarom, 2000) and calcium wave propagation mediated by receptors on the endoplasmic reticulum (Ross, 2012). These distinct intrinsic properties, including excitability, have been shown to undergo bidirectional changes in a manner that is local to specific neuronal locations or is global spanning all locations (Brager & Johnston, 2007; Das et al., 2017; Johnston & Narayanan, 2008; Narayanan, Dougherty, & Johnston, 2010; Narayanan & Johnston, 2007; Narayanan & Johnston, 2008).

As the protocols employed for inducing nonsynaptic (including intrinsic and structural) plasticity are at most instances identical to synaptic plasticity induction protocols, the broad mechanisms involved in the induction and in the translation of induction to expression are very similar to those for synaptic plasticity (Figure 8). Specifically, induction of intrinsic plasticity requires influx of cytosolic calcium with different kinetics and strengths of calcium translating to distinct strengths and directions of intrinsic plasticity (Brager & Johnston, 2007; Fan et al., 2005; Huang et al., 2005; Sjostrom et al., 2008; Wang et al., 2003). The components that mediate calcium entry for synaptic plasticity also mediate calcium entry for nonsynaptic plasticity, including NMDA receptors (Chung, Ge, et al., 2009; Chung, Qian, et al., 2009; Engert & Bonhoeffer, 1999; Fan et al., 2005; Frick et al., 2004; Huang et al., 2005; Lin et al., 2008; Losonczy et al., 2008; Matsuzaki, Honkura, Ellis‐Davies, & Kasai, 2004; Nagerl, Eberhorn, Cambridge, & Bonhoeffer, 2004; Narayanan & Johnston, 2007; Tonnesen, Katona, Rozsa, & Nagerl, 2014; Wang et al., 2003), voltage‐gated calcium channels (Chung, Ge, et al., 2009; Chung, Qian, et al., 2009; Lin et al., 2008; Wang et al., 2003) and receptors on the ER (Ashhad, Johnston, & Narayanan, 2015; Brager & Johnston, 2007; Brager et al., 2013; Clemens & Johnston, 2014; Kim, Brager, & Johnston, 2018; Narayanan et al., 2010). This implies that the arguments (3.3, 3.4) placed about synergistic interactions between different calcium sources and about degeneracy in the induction of synaptic plasticity extends to the induction of nonsynaptic plasticity as well (Figure 8).

As a direct consequence of the similarity in the protocols employed in inducing synaptic and intrinsic plasticity, the downstream mechanisms that mediate the translation from induction of nonsynaptic plasticity to its expression are also similar (Shah et al., 2010) to those that mediate a similar transition in synaptic plasticity (Figure 8). Several signaling cascades that are present on the pre‐ and post‐synaptic sides mediate this translation, with retrograde messengers acting as mechanisms that signal the elevation of postsynaptic calcium to the presynaptic terminals. Specifically, the same set of enzymes and messengers that mediate synaptic plasticity also mediate nonsynaptic plasticity (Figure 8). Examples to this equivalence include nonsynaptic forms of plasticity that are mediated by CaMKII (Fan et al., 2005; Huang et al., 2005; Lujan et al., 2009; Matsuzaki et al., 2004; Wang & Wagner, 1999), PKA (Lin et al., 2008; Narayanan et al., 2010; Rosenkranz et al., 2009) and MAPK (Rosenkranz et al., 2009; Yuan, Adams, Swank, Sweatt, & Johnston, 2002). However, there could be dissociation between the mechanisms that are involved in the translation to the expression of different forms of plasticity that are consequent to the *same* induction protocol, where different enzymes and messengers mediate different forms of plasticity (Brager & Johnston, 2007; Fan et al., 2005; Lin et al., 2008; Rosenkranz et al., 2009; Wang et al., 2003). As mentioned earlier (Section 3.5), the expression of plasticity in synapses could be mediated by plasticity in voltage‐gated calcium channels that are expressed in the presynaptic terminal, mediated by retrograde messengers and presynaptic signaling cascades, or by change in mechanisms that alter postsynaptic excitability, thus blurring the distinction between synaptic and certain forms of nonsynaptic plasticity.

Following the activation of different signaling cascades, akin to the expression of synaptic plasticity, several molecular processes, including synthesis, trafficking and post‐translational modification of the several membrane and cytosolic proteins, mediate the final step towards the expression of distinct forms of nonsynaptic plasticity (Figure 8). The mechanisms behind the trafficking of several ion channels have been studied (Cusdin, Clare, & Jackson, 2008; Jensen, Rasmussen, & Misonou, 2011; Lai & Jan, 2006; Lau & Zukin, 2007; Lujan et al., 2009; Shah et al., 2010; Vacher et al., 2008; Wenthold, Prybylowski, Standley, Sans, & Petralia, 2003), and it is now clear that plasticity is ubiquitous (Kim & Linden, 2007). In addition to these changes in cytosolic and membrane proteins, it has been shown that hippocampal spines undergo continuous structural changes, apart from demonstrations of distinct forms of structural plasticity in spines, dendrites and axons (Attardo, Fitzgerald, & Schnitzer, 2015; Chen, Lu, & Zuo, 2014; Emoto, 2011; Engert & Bonhoeffer, 1999; Ghiretti & Paradis, 2014; Grubb & Burrone, 2010a; Grubb & Burrone, 2010b; Grubb et al., 2011; Ikegaya et al., 2001; Johnston et al., 2016; Jungenitz et al., 2018; Luo & O'Leary, 2005; Matsuzaki et al., 2004; Nagerl et al., 2004; Tonnesen et al., 2014; Yuste & Bonhoeffer, 2001). Finally, the dynamics associated with the various glial functions and their interactions with neuronal and metabolic pathways could also undergo changes in response to behavioral experiences and activity (Araque et al., 2014; Ashhad & Narayanan, 2019; Baumann & Pham‐Dinh, 2001; Fields, 2010; Halassa & Haydon, 2010; Haydon & Carmignoto, 2006; Khakh & Sofroniew, 2015; Pannasch & Rouach, 2013; Perea et al., 2016; Sierra, Tremblay, & Wake, 2014; Volterra, Liaudet, & Savtchouk, 2014). It is therefore clear that there is no escape from the conclusion that activity‐ or experience‐ or pathology‐dependent plasticity does not confine itself to a few constitutive components, but is rather expansive and even ubiquitous (Kim & Linden, 2007). There are considerable overlaps in the mechanisms that mediate the induction and expression of these forms of plasticity, and many‐to‐one and one‐to‐many mappings between the induction protocol (or behavioral experience) and achieving specific levels of plasticity in specific components (Figure 8).

In summary, the lines of evidence provided above point to ample evidence for degeneracy in the process of their induction and expression of different forms of plasticity and their combinations, both in terms of their individual strengths and directions. This also implies that the same functional changes could be achieved through distinct combinations of plasticity mechanisms, thus pointing to a further dissociation between functional homeostasis and the plasticity mechanisms that yielded it. In other words, functional equivalence in terms of transition from one state to another does not necessarily translate to plasticity equivalence (where the route taken to achieve the transition is always identical). An important class of plasticity models has recognized the ubiquitous nature of plasticity, with models built within this framework of plasticity degeneracy. These models account for concomitant changes in multiple components, also accounting for disparate combinations of plasticity resulting in similar functional outcomes, rather than assuming plasticity equivalence in the face of functional equivalence (Abbott & LeMasson, 1993; Anirudhan & Narayanan, 2015; LeMasson et al., 1993; Mukunda & Narayanan, 2017; O'Leary et al., 2013; O'Leary et al., 2014; Siegel et al., 1994; Srikanth & Narayanan, 2015). Future theoretical and experimental investigations into hippocampal plasticity should therefore account for the truly ubiquitous nature of plasticity in designing their experiments and addressing outstanding questions, rather than assuming that plasticity is confined to one single component or the other (Bhalla, 2014; Kim & Linden, 2007).

### 3.7 Degeneracy in metaplasticity and in maintaining stability of learning

**Figure 9:**
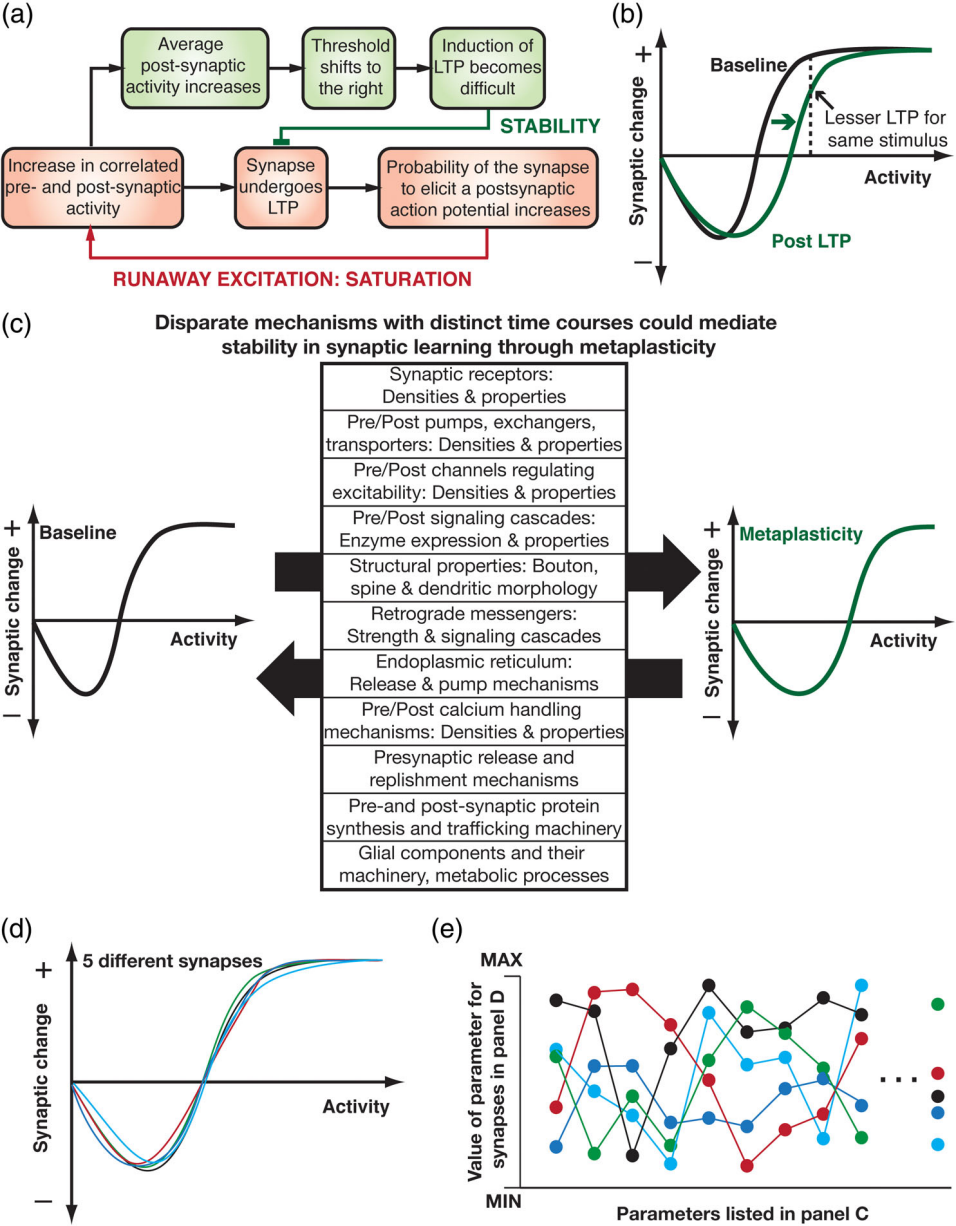
Disparate mechanisms with distinct time courses could mediate stability in synaptic learning through metaplasticity. (a, b) Hebbian synaptic plasticity is inherently unstable leading to runaway excitation in synaptic structure (a; orange boxes). The Bienenstock–Cooper–Munro (BCM) rule envisages the existence of a sliding threshold mechanism (b) which provides a negative feedback loop (b; green boxes) that would preclude runaway excitation by altering the rules for plasticity. Alteration of plasticity rules has been referred to as metaplasticity in the literature (Abraham & Bear, 1996; Bienenstock, Cooper, & Munro, 1982; Cooper & Bear, 2012). (c) Bidirectional metaplasticity could be mediated by any of the several mechanisms discussed in Figures 7 and 8 with reference to the expression of synaptic and nonsynaptic plasticity. (d,e) Similar plasticity profiles (d) could be achieved through disparate combinations of constituent parameter values (e). Cartoon illustrations are derived from conclusions drawn in previous studies (Abraham, 2008; Abraham & Bear, 1996; Anirudhan & Narayanan, 2015; Hulme et al., 2013; Sehgal et al., 2013) [Color figure can be viewed at wileyonlinelibrary.com]

Hebbian synaptic plasticity is inherently unstable. In the absence of concomitant homeostatic mechanisms, Hebbian plasticity would result in runaway excitation (Figure 9). Several theories and mechanisms have been proposed as a means to avoid this runaway excitation (Abbott, 2003; Abraham & Robins, 2005; Benuskova & Abraham, 2007; Honnuraiah & Narayanan, 2013; Jedlicka, Benuskova, & Abraham, 2015; Korte & Schmitz, 2016; Miller & MacKay, 1994; Nelson & Turrigiano, 2008; Turrigiano, 2007; Turrigiano, 2011; Turrigiano, 1999; Turrigiano, 2008; Turrigiano, 2017; Turrigiano & Nelson, 2000; van Rossum, Bi, & Turrigiano, 2000; Zenke, Gerstner, & Ganguli, 2017). A prominent theme that spans several such stability theories is metaplasticity (Abraham, 2008; Abraham & Bear, 1996; Abraham & Tate, 1997; Hulme et al., 2013), where the profile of plasticity concomitantly changes with the induction of plasticity (Figure 9a,b). An extremely useful mathematical treatise that has helped in the understanding metaplasticity and stability, especially for synaptic plasticity profiles in the hippocampus, is the Bienenstock–Cooper–Munro (BCM) rule (Bienenstock et al., 1982; Cooper & Bear, 2012; Shouval et al., 2002; Yeung, Shouval, Blais, & Cooper, 2004). This is despite the observation that the BCM framework and the synaptic plasticity framework in hippocampal synapses are not completely analogous to each other (Cooper, Intrator, Blais, & Shouval, 2004). It should also be noted that not all synapses follow a BCM‐like synaptic plasticity profile, and therefore a stability theory dependent on this rule is not generalizable to all synapses (Abbott & Nelson, 2000; Jorntell & Hansel, 2006).

Although the utility of BCM‐like synaptic rule in understanding stability in synaptic learning has been invaluable, the exact mechanisms that mediate the sliding modification threshold and the consequent metaplasticity has remained an open question. Several mechanisms (Figure 9c) involving changes in morphological characteristics, several receptors, ion channels and signaling cascades have been proposed as candidates for this role (Abraham, 2008; Abraham & Bear, 1996; Abraham, Mason‐Parker, Bear, Webb, & Tate, 2001; Abraham & Tate, 1997; Anirudhan & Narayanan, 2015; Bear, 2003; Bear, Cooper, & Ebner, 1987; Cooper & Bear, 2012; Hulme et al., 2013; Kalantzis & Shouval, 2009; Narayanan & Johnston, 2010; Philpot, Espinosa, & Bear, 2003; Philpot, Sekhar, Shouval, & Bear, 2001; Sehgal et al., 2013; Triesch, 2007). As any change in mechanisms that regulate the induction or expression of synaptic plasticity would result in a change in plasticity profiles (3.3, 3.5), it is not surprising that mechanisms that regulate synaptic plasticity are candidate mechanisms that mediate metaplasticity. Similar to the argument placed with reference to the mechanisms that mediate the expression of synaptic plasticity, the framework of degeneracy provides an elegant solution to the question on which of these is *the* mechanism that mediates the sliding modification threshold within a BCM‐like plasticity framework. It offers reconciliation to this conundrum by suggesting that disparate combinations of these distinct mechanisms could result in similar plasticity profiles (Figure 9d,e), thereby suggesting degeneracy in the emergence of metaplasticity and stability in synaptic learning (Anirudhan & Narayanan, 2015). Finally, it was traditionally assumed that stability and homeostatic mechanisms are slower compared to the encoding mechanisms. However, there are several lines of theoretical and experimental evidence, spanning several synaptic and intrinsic components as candidate mechanisms, for *concurrent* emergence of encoding, stability *and* activity homeostasis. These lines of evidence also argue for prominent advantages when encoding, homeostasis and stability mechanisms are *concurrent* (Abraham, Logan, Wolff, & Benuskova, 2007; Anirudhan & Narayanan, 2015; Benuskova & Abraham, 2007; Honnuraiah & Narayanan, 2013; Ibata, Sun, & Turrigiano, 2008; Jedlicka et al., 2015; Johnston & Narayanan, 2008; Narayanan & Johnston, 2007; Narayanan & Johnston, 2010; Nelson & Turrigiano, 2008; O'Leary et al., 2014; Triesch, 2007; Turrigiano, 2011; Turrigiano, 2008; Turrigiano, 2017; Zenke et al., 2017).

Within the framework of degeneracy, the goal of *concomitantly* achieving encoding‐driven plasticity, activity homeostasis and stable learning is achieved through disparate combinations of synaptic, intrinsic, glial and structural plasticity. With abundant experimental evidence for plasticity in each of these different components occurring in an activity‐ or experience‐dependent manner (Section 3.6), it is important that the analyses of stable learning broaden their focus beyond the narrow realm of stable *synaptic* learning. The current theories implicitly or explicitly assume that encoding is driven by synaptic plasticity, with several mechanisms contributing to the stability of this synaptic learning system. The metaplasticity framework also largely focuses on plasticity of *synaptic* plasticity profiles, although the mechanisms that mediate several forms of plasticity overlap with each other (Section 3.6). Future frameworks should therefore analyze concomitant learning *and* stability as a consequence of disparate forms of plasticity (Henderson & Gong, 2018), also assessing *metaplasticity of intrinsic, glial and structural plasticity*. While plasticity in synaptic structures form *a component* of learning and stability, given the abundant lines of experimental evidence on ubiquitous plasticity, it is extremely critical that learning and stability theories broaden their horizon to encompass all forms of plasticity and degeneracy therein.

### 3.8 Degeneracy in the generation and regulation of local field potentials

Extracellular field recordings are useful readouts of network activity in a given brain region. Local field potentials (LFP), the low pass filtered version of field recordings have traditionally been thought to provide information about afferent synaptic activity. LFPs recorded from within the hippocampus exhibit signature activity patterns that are dependent on the behavioral state of the animal. For instance, they manifest strong oscillations in the theta frequency range (4–10 Hz) during exploratory behavior and during rapid eye moment (REM) sleep, and show characteristic sharp‐wave ripple patterns during rest and non‐REM sleep. These distinct activity patterns have been postulated to serve specific functions such as in the consolidation of memory and in neural encoding of space (Buzsaki, 1986; Buzsaki, 1989; Buzsaki, 2002; Buzsaki, 2006; Buzsaki, 2015; Buzsaki & Moser, 2013; Colgin, 2013; English et al., 2014; Grosmark, Mizuseki, Pastalkova, Diba, & Buzsaki, 2012; Hartley et al., 2014; Lisman & Jensen, 2013; Mizuseki & Buzsaki, 2014; Montgomery, Sirota, & Buzsaki, 2008; Moser et al., 2008; Moser, Rowland, & Moser, 2015; Tononi & Cirelli, 2006; Wilson & McNaughton, 1994; Ylinen et al., 1995; Ylinen et al., 1995).

Although these signature patterns of extracellular events manifest as repeating motifs, there are strong lines of theoretical and experimental evidence that they emerge from very disparate structures. For instance, theta oscillations in the hippocampus have shown to be afferent from two reciprocally connected subcortical nuclei that act as pacemakers, the medial septum‐diagonal band of Broca and the supramammillary region. Apart from these two subcortical nuclei, inputs from entorhinal cortex and CA3 also play an important role in the generation of theta oscillations in the hippocampus. Furthermore, theoretical modeling and in vitro data also suggest that an intact hippocampus could sustain theta oscillations on its own in a manner that is dependent on intra‐hippocampal excitatory and inhibitory synaptic connections (Buzsaki, 2002; Buzsaki, 2006; Colgin, 2013; Colgin, 2016; Goutagny, Jackson, & Williams, 2009; Kamondi, Acsady, Wang, & Buzsaki, 1998; Traub, Miles, & Wong, 1989).

A similar analysis, in terms of disparate underlying sources and mechanisms, holds for gamma frequency oscillations that are observed in the hippocampus as well (Buzsaki & Wang, 2012; Colgin, 2016; Colgin & Moser, 2010; Csicsvari, Jamieson, Wise, & Buzsaki, 2003; Wang, 2010; Wang & Buzsaki, 1996). In addition, apart from synaptic contributions to the LFPs, it is now clear that return transmembrane currents from sub‐ and supra‐threshold somatodendritic ion channels also alter the LFP in terms of their frequency content, amplitude and phase (Buzsaki, Anastassiou, & Koch, 2012; Einevoll, Kayser, Logothetis, & Panzeri, 2013; Ness, Remme, & Einevoll, 2016; Reimann et al., 2013; Schomburg, Anastassiou, Buzsaki, & Koch, 2012; Sinha & Narayanan, 2015; Taxidis, Anastassiou, Diba, & Koch, 2015). In addition, several mechanisms such ephaptic coupling, heterogeneous extracellular resistivity, glial and axonal transmembrane mechanisms also contribute and regulate local field potentials, resulting in a complexity that spans almost all parameters of the local network (Anastassiou & Koch, 2015; Buzsaki et al., 2012; Einevoll et al., 2013; Kajikawa & Schroeder, 2011; Katzner et al., 2009; Linden et al., 2011).

From the complexity involved in the generation and regulation of hippocampal LFPs, with several brain regions and several constitutive network components contributing to their emergence, it is easy to discern that similar LFP patterns could be achieved through nonunique combinations of disparate components. Irrespective of whether it is the manifestation of an oscillatory pattern in a given frequency range (Buzsaki, 2002; Buzsaki & Wang, 2012; Colgin, 2013; Colgin & Moser, 2010; Csicsvari et al., 2003), or the emergence of sharp wave ripples (Buzsaki, 2015; English et al., 2014; Taxidis et al., 2015), or the emergence of resonance in the LFP power spectral density (Ness et al., 2016), or achieving a given phase of single‐neuron firing with reference to an LFP oscillation (Sinha & Narayanan, 2015), the routes are several and involve several disparate structural components. Thus, there is evidence for degeneracy in the mechanisms that mediate and regulate local field potentials, implying that extreme caution should be exercised in making one‐to‐one relationships between constitutive components and specific aspects of LFP recordings (Anastassiou & Koch, 2015; Buzsaki et al., 2012; Einevoll et al., 2013; Kajikawa & Schroeder, 2011; Katzner et al., 2009; Linden et al., 2011).

### 3.9 Degeneracy in neural coding

A particularly thorny debate that has spanned decades is about the codes employed by neurons in encoding their inputs. The crux of the debate has been about whether neurons encode information in the rate of or in the precise timing of action potential firing (Buzsaki, Logothetis, & Singer, 2013; Engel, Fries, & Singer, 2001; Engel & Singer, 2001; Fries, Nikolic, & Singer, 2007; Gallistel, 2017; Jaramillo & Kempter, 2017; London, Roth, Beeren, Hausser, & Latham, 2010; Panzeri et al., 2017; Shadlen & Newsome, 1994; Shadlen & Newsome, 1995; Shadlen & Newsome, 1998; Singer et al., 1997; Softky, 1994; Softky, 1995). Arguments against temporal coding have raised questions about the ability of neurons to perform millisecond‐or‐submillisecond coincidence detection that is essential for decoding a temporal code, about the relevance of precise timing in the face of noise and variability in neuronal responses to identical stimuli and about the ability of neuronal networks to reliably propagate synchronous firing (London et al., 2010; Panzeri et al., 2017; Shadlen & Newsome, 1994; Shadlen & Newsome, 1998). Counterarguments have relied on the demonstration of millisecond‐or‐submillisecond coincidence detection in active dendritic structures, on the dependence of synchrony propagation on neuronal intrinsic properties *and* input structure and on the existence of temporally precise cell assemblies that could mitigate the overall background noise in decoding the precise timing of inputs (Buzsaki, 2010; Buzsaki et al., 2013; Das & Narayanan, 2015; Das & Narayanan, 2017; Diesmann, Gewaltig, & Aertsen, 1999; Engel et al., 2001; Engel & Singer, 2001; Fries et al., 2007; Golding & Oertel, 2012; Hong, Ratte, Prescott, & De Schutter, 2012; Pastalkova, Itskov, Amarasingham, & Buzsaki, 2008; Reyes, 2003; Singer et al., 1997; Softky, 1994).

The expression of *coding* degeneracy in the cellular and network scales (Leonardo, 2005), in terms of the ability of disparate structural components to elicit similar input‐output characteristics, is clear from the lines of evidence presented earlier (Section 2.2). In addition, employing electrophysiological recordings and computational models to assess subthreshold resonance and spike triggered average (STA) of model neurons, it has been shown that hippocampal pyramidal neurons are selective to different input features (including spectral features and temporal coincidence of inputs) depending on the dendritic location of their inputs. This location‐dependent feature encoding is mediated by ion channel expression profiles, and could be achieved through disparate combinations of different ion channel expression profiles (Das & Narayanan, 2014; Das & Narayanan, 2015; Das & Narayanan, 2017; Das et al., 2017; Narayanan & Johnston, 2007; Narayanan & Johnston, 2012; Rathour et al., 2016; Rathour & Narayanan, 2012a; Rathour & Narayanan, 2014). Given the well‐established strong relationship between STA and types of coding (Ratte, Hong, De Schutter, & Prescott, 2013), this location‐dependent scenario argues for location‐dependent forms of coding. Specifically, the soma and proximal dendrites show class I STA (integrator) and the distal dendrites manifest class II STA (coincidence detector) as a consequence of the differential expression of different channels (Das & Narayanan, 2015). Therefore, it seems reasonable to postulate that the proximal and distal regions are respectively geared towards rate and temporal coding, with this location‐dependent differential coding strategy extending to cortical and hippocampal neurons (Branco & Hausser, 2010; Branco & Hausser, 2011; Das & Narayanan, 2015). Finally, behaviorally‐driven neuromodulatory inputs and activity‐dependent plasticity could markedly alter the operating mode and the class of excitability of compartments of a single neuron, and the type of coding employed by a neuron is dependent not just on its operating mode but also the specific characteristics of the input. Thus, even from the perspective of encoding strategies *within* a single neuron, the arguments that pitch rate coding *against* temporal coding are oversimplifying the complexity of neural encoding and decoding. Instead, there are broad lines of evidence pointing to a hybrid rate/temporal coding system that encompasses degeneracy by achieving encoding goals through disparate combinations of several cellular and network components in a manner that is strongly dependent on several spatiotemporal aspects of neuronal and behavioral state (Das & Narayanan, 2014; Das & Narayanan, 2015; Das et al., 2017; Diesmann et al., 1999; Lee & Dan, 2012; Marder, 2012; Marder & Thirumalai, 2002; Ratte et al., 2013).

With reference to neural codes for features of the external environment, the coding of spatial location of animal in the hippocampus is an ideal instance of hybrid encoding schema that expresses degeneracy. Unlike the argument for rate versus temporal coding that seems to drive the narrative otherwise (Buzsaki et al., 2013; Engel et al., 2001; Engel & Singer, 2001; Fries et al., 2007; Gallistel, 2017; Jaramillo & Kempter, 2017; London et al., 2010; Panzeri et al., 2017; Shadlen & Newsome, 1994; Shadlen & Newsome, 1995; Shadlen & Newsome, 1998; Singer et al., 1997; Softky, 1994; Softky, 1995; Srivastava et al., 2017), hippocampal physiologists have concurred on the existence of dual/hybrid encoding schema for place‐specific encoding. Specifically, place cells in the hippocampus elicit higher rates of firing when the animal enters a specific place field. In conjunction, the phase of action potential firing of place cells with reference to the extracellular theta rhythm also advances as a function of spatial location of the animal within the place field. Thus, hippocampal place cells employ a dual code of firing rate *and* phase of firing (temporal coding involving the precise timing of action potential firing) to represent spatial location of the animal (Ahmed & Mehta, 2009; Buzsaki & Moser, 2013; Derdikman & Moser, 2010; Hartley et al., 2014; Harvey, Collman, Dombeck, & Tank, 2009; Huxter, Burgess, & O'Keefe, 2003; Lisman, 2005; Lisman & Jensen, 2013; Mehta, Lee, & Wilson, 2002; Moser et al., 2008; Moser et al., 2015; O'Keefe, 1976; O'Keefe, 1979; O'Keefe & Burgess, 1999; O'Keefe & Burgess, 2005; O'Keefe, Burgess, Donnett, Jeffery, & Maguire, 1998; O'Keefe & Conway, 1978; O'Keefe & Recce, 1993; Skaggs, McNaughton, Wilson, & Barnes, 1996). In certain cases, it has been shown that the two coding schema act independent of each other and could act as the fail‐safe mechanisms for each other (Aghajan et al., 2015; Huxter et al., 2003).

Whereas these lines of evidence make a case for employing disparate coding schemas in encoding the same input, the case for disparate mechanisms involved in encoding and maintaining the rate and temporal codes is also strong. Specifically, the role of afferent synaptic drive, local inhibition, several ion channels and receptors, dendritic spikes, spatiotemporal interactions between somatodendritic channels and receptors, and plasticity in each of these components have all been implicated in the emergence and maintenance of these codes (Basak & Narayanan, 2018b; Bittner et al., 2015; Danielson et al., 2016; Geisler et al., 2010; Geisler, Robbe, Zugaro, Sirota, & Buzsaki, 2007; Grienberger, Milstein, Bittner, Romani, & Magee, 2017; Harvey et al., 2009; Lee, Lin, & Lee, 2012; Losonczy, Zemelman, Vaziri, & Magee, 2010; Magee, 2001; Nakashiba, Young, McHugh, Buhl, & Tonegawa, 2008; Nakazawa, McHugh, Wilson, & Tonegawa, 2004; Nolan et al., 2004; Royer et al., 2012; Sheffield & Dombeck, 2015; Skaggs et al., 1996; Tsien et al., 1996; Wills, Lever, Cacucci, Burgess, & O'Keefe, 2005). In addition, there are lines of experimental evidence that suggest that subthreshold afferent synaptic inputs from several place fields arrive onto a single place cell, and that a silent cell could be converted to a place cell for *any* of these place fields by an appropriate plasticity‐inducing stimulus (Bittner et al., 2015; Lee et al., 2012), suggesting that disparate cells could achieve the same function of encoding a given spatial location. The expression profiles of several channels and receptors control the overall excitability of a neuron (Section 2.2), and there are several mechanisms that regulate the phase of intracellular voltage oscillations with reference to an external reference or to the overall afferent current (Geisler et al., 2010; Geisler et al., 2007; Harvey et al., 2009; Narayanan & Johnston, 2008; Rathour et al., 2016; Rathour & Narayanan, 2012a; Rathour & Narayanan, 2014; Sinha & Narayanan, 2015; Skaggs et al., 1996). Together, these studies point to the possibility that similar rate *and* phase spatial codes in a neuron could be achieved through disparate combinations of constituent components, and several neurons could encode for the same place field with distinct combinations of these mechanisms. Future studies could further explore the manifestation of degeneracy in spatial coding in the hippocampus, focusing on the hybrid code involving rate as well as phase encoding of input features.

### 3.10 Degeneracy in learning and memory

Behavior emerges as a consequence of coordinated activity of multiple brain regions in conjunction with sensory and motor systems (Bennett & Hacker, 2003; Jazayeri & Afraz, 2017; Krakauer et al., 2017; Tytell et al., 2011; Vetere et al., 2017). The hippocampus has been implicated in several forms of spatial and nonspatial learning, with strong links to episodic memory (Anderson et al., 2007; Bird & Burgess, 2008; Bliss & Collingridge, 1993; Bunsey & Eichenbaum, 1996; Lynch, 2004; Marr, 1971; Martin et al., 2000; Martinez and Derrick, Martinez Jr. & Derrick, 1996; Mayford et al., 2012; Morris, 1989; Morris et al., 1986; Morris, Garrud, Rawlins, & O'Keefe, 1982; Moser et al., 2015; Nakazawa et al., 2004; Neves, Cooke, & Bliss, 2008; Rajasethupathy et al., 2015; Scoville & Milner, 1957; Squire, Stark, & Clark, 2004; Whitlock, Heynen, Shuler, & Bear, 2006).

The quest for *the* mechanistic basis for learning and memory in the hippocampus has spanned several decades, especially since the strong links between the hippocampal lesions and specific forms of memory were established (Scoville & Milner, 1957). This quest has spanned several scales of analysis, with efforts to link specific genes, receptors, channels and forms of cellular plasticity to learning and memory. Several studies have assessed the link between specific behavioral tasks and cellular/molecular substrates through targeted pharmacological blockades or genetic manipulations. The existence of divergent and numerous cellular/molecular components that impair *specific* learning tasks have been unveiled by these efforts, revealing considerable complexity in the plasticity networks and systems biology of learning and memory. As is evident from this complexity and associated animal‐to‐animal and cell‐to‐cell variability (which involves the ensemble of mechanisms and interactions discussed above not just from within the hippocampus but also from other brain regions), demonstrating causality with reference to learning and memory and *any one specific form of plasticity or cellular/molecular substrate*, has proven extremely challenging (Andersen, Morris, Amaral, Bliss, & O'Keefe, 2006; Bennett & Hacker, 2003; Bhalla, 2014; Bhalla & Iyengar, 1999; Bliss & Collingridge, 1993; Collingridge & Bliss, 1987; Jazayeri & Afraz, 2017; Kandel, 2001; Kandel et al., 2014; Kim & Linden, 2007; Kotaleski & Blackwell, 2010; Krakauer et al., 2017; Lynch, 2004; Manninen et al., 2010; Martin et al., 2000; Martinez and Derrick, 1996; Mayford et al., 2012; Mozzachiodi & Byrne, 2010; Neves, Cooke, & Bliss, 2008; Zhang & Linden, 2003).

The complexities of the networks that are involved in learning and memory are only compounded by the many‐to‐many mappings that are observed between behavioral observations and molecular/cellular components, the joint occurrence of several forms of plasticity with the *same* protocols (Section 3.6), the concurrent impairments in different forms of plasticity by blockade of the *same* signaling cascades (Section 3.6), the dissociations between different learning tasks and the compensatory mechanisms that are associated with the knockout of specific genes (Bailey, Rustay, & Crawley, 2006; Jazayeri & Afraz, 2017; Krakauer et al., 2017; Mayford et al., 2012; Tsokas et al., 2016). For instance, the knockout of GluA1 (also referred to as GluR1 or GluRA), an AMPAR subunit that is important for expression of certain forms of synaptic plasticity, impaired only some forms of synaptic plasticity and not others at the cellular scale of analysis (Hoffman, Sprengel, & Sakmann, 2002; Phillips, Hardingham, & Fox, 2008; Zamanillo et al., 1999). Similarly, at the behavioral level, although behavioral deficits were observed in certain learning tasks in GluA1 knockout mice, the knock out did not alter behavior in other learning tasks (Reisel et al., 2002; Zamanillo et al., 1999). Several examples of such dissociations are reviewed in (Mayford et al., 2012), further emphasizing the difficulty in assigning a causal link between learning and memory and *any one specific form of plasticity or cellular/molecular substrate*.

Although this parametric and interactional complexity might seem exasperating if the goal is to pinpoint *the* cellular/molecular component that is involved in hippocampal‐dependent learning and memory, it is an extremely useful substrate for the effective expression of degeneracy in achieving the goal of robust learning and memory. The ability to achieve very similar learning indices through multiple routes involving disparate forms of plasticity in several constitutive components tremendously increases the ability of the system to achieve robust learning. As a consequence of the several forms of variability and state‐dependence exhibited by the learning system, in terms of the underlying components, their plasticity and combinatorial interactions, it is possible that some of these disparate routes may not involve specific cellular/molecular components or forms of plasticity in the process of achieving certain learning goals. This also implies animal‐to‐animal and trial‐to‐trial variability in the mechanisms that mediate learning, thereby calling for utmost caution in assigning one‐to‐one relationships between behavioral learning and specific forms of plasticity in any single brain region (Bailey et al., 2006; Bennett & Hacker, 2003; Jazayeri & Afraz, 2017; Krakauer et al., 2017; Mayford et al., 2012; O'Leary & Marder, 2014; Sieling, Bedecarrats, Simmers, Prinz, & Nargeot, 2014; Tsokas et al., 2016; Vogelstein et al., 2014).

## 4 THE CAUSALITY CONUNDRUM

It is clear from the analyses above that theoretical and experimental evidence exist for: (a) several disparate combinations of distinct constitutive components elicit analogous function; (b) there are forms of animal‐to‐animal (channel‐to‐channel, neuron‐to‐neuron, network‐to‐network, etc.) variability in terms of the contributions of specific constitutive components that mediate similar function; and (c) the components that mediate similar function, and their relative contributions to the emergence of this function are state‐dependent, and could undergo experience‐dependent plasticity (towards maintaining robustness of that function or towards learning‐dependent alteration of function). Juxtaposed against these observations is the question on whether it is even possible to exclusively assign causal one‐to‐one relationships between function and specific constitutive components. Evidence for the existence of degeneracy, variability and adaptability have made us acutely aware of the possibility that we could be committing mereological fallacies (Bennett & Hacker, 2003; Varzi, 2016), whereby we assign specific behavioral roles to parts of the animal's brain or to plasticity therein (Bailey et al., 2006; Jazayeri & Afraz, 2017; Krakauer et al., 2017; Mayford et al., 2012; O'Leary & Marder, 2014; Sieling et al., 2014; Tsokas et al., 2016; Vogelstein et al., 2014).

### 4.1 Inevitable flaws in an experimental plan to establish causality that leaps across multiple scales

Let us chart a hypothetical experimental plan where we are interested in demonstrating that a specific form of learning behavior is dependent on plasticity in one specific component (let us say component X) in a brain region of our choice (let us say hippocampus). We first measure in vivo plasticity in component X along with its time course, and let us say that we find a prominent correlation between this time course and the time course of behavioral learning. Next, we introduce an established blocker of plasticity in component X specifically into the hippocampus, and find that this blocks both the plasticity in component X in vivo and impairs learning. We repeat similar experiments with (a) an established pharmacological blocker of component X infused into the hippocampus; (b) transgenic manipulations that take out component X completely in the hippocampus; (c) a pharmacological blocker that leaves component X intact, but impairs its plasticity by blocking a mechanism that induces plasticity in component X; and (d) genetic knockout of mechanisms that mediates plasticity in component X. Let us say that learning was impaired in all four cases, and there was no plasticity in component X in the last two cases (in the first two cases component X was completely abolished). As a final experiment in testing the hypothesis to link plasticity in hippocampal component X to the specific learning behavior, we artificially alter component X and consequently find behavioral signatures related to the learning process. Therefore, we have shown that component X and its plasticity are necessary and sufficient for the specific learning behavior. This experimental plan is broadly similar to that proposed by (Stevens, 1998) to test the hypothesis that auditory synapses in the amygdala become strengthened by LTP during behavioral training that attaches “fear” to the tone, and that the memory of the tone as a fear‐producing stimulus resides in the strength of the synapses from the auditory thalamus (Stevens, 1998):

“How could this idea be tested? It should be that (1) blocking LTP prevents fear learning; (2) the sensory pathways from the thalamus and cortex to the amygdala are capable of LTP; (3) auditory fear conditioning increases the amygdala's postsynaptic response to the tone, and these increases are prevented by blocking LTP pharmacologically or in another way; and (4) inducing LTP in the thalamoamygdaloid pathway attaches “fear” to appropriate sensory stimuli.”

Although this experimental plan has shown that component X and its plasticity are necessary and sufficient for the specific learning behavior, given the complexity that we have elucidated thus far, this experimental design *does not* provide a causal link between component X or its plasticity with behavior. First, we were so focused on component X that we implicitly precluded the change of any other component either in the hippocampus or in other brain region. Given the rich complexity in the distinct components, their plasticity and interactions among them, it is infeasible that only component X in the hippocampus was changing in response to the behavioral stimulus. It is now well established that several cellular components change in response the same calcium signal or the activation of the same signaling cascade, and there are several parallel homeostasis mechanisms as well. This implies that altering component X in the hippocampus *without* altering anything else across the brain is highly unlikely. Therefore, if we had performed the same set of experiments on another component Y, we might have arrived at similar conclusions (including correlated time courses). In other words, it is important not to interpret measurement correlations as evidence for causation, and to understand that absence of measurements in other forms of plasticity or plasticity in other brain regions does not mean they do not coexist with the form of plasticity that we are focused on.

Second, when we blocked plasticity in component X, given the complexities elucidated above, it is highly unlikely that we *specifically* blocked plasticity in component X without disturbing plasticity in any other measurement or without introducing metaplasticity in some other form of plasticity (3.6, 3.7). For instance, from a cellular perspective, theta burst pairing results in plasticity of synaptic strength and of HCN, *A*‐type K^+^ and SK channels, and pharmacologically blocking NMDAR receptors impairs plasticity not just in one of them, but in *all* of them (Fan et al., 2005; Frick et al., 2004; Lin et al., 2008; Losonczy et al., 2008). Thus if we had observed impairment of plasticity in only one of these components, we would have wrongly concluded that to be the only component that changes with TBP. Returning to our experimental plan on the role of component X, there could also be several other secondary and unintended effects of blocking plasticity in component X that spans the hippocampus and other brain regions (Bhalla, 2014; Jazayeri & Afraz, 2017; Kotaleski & Blackwell, 2010; Krakauer et al., 2017; Otchy et al., 2015). Thus, it is prudent not to dismiss absence of measurements as absence of evidence for other components being involved in the overall behavior.

Third, when we performed the experiment of artificially altering component X, it is obvious that it is highly unlikely that we achieved this without disturbing any other component in some brain region or without introducing metaplasticity in some form of plasticity. Therefore, the alternate interpretations of our observations (other than the “linear narrative” that concludes “plasticity in hippocampal component X mediates learning behavior”) are innumerable given the staggering complexity of the underlying system and the degeneracy involved in accomplishing the learning task. Ruling out *all these* alternate interpretations is essential for convergence to the linear narrative, but is rather impossible because measurements of all constitutive components in all brain regions is currently infeasible. From a nonlinear dynamical system perspective (Guckenheimer & Holmes, 1983; Nayfeh & Balachandran, 1995; Strogatz, 2014), our “linear narrative” and the associated inference are equivalent to declaring a component to be critically important for system performance because perturbation to that one component—which is part of a high‐dimensional, adaptive, nonlinear dynamical system with strong coupling across dimensions—collapses the system. Additionally, especially given the expression of degeneracy, in our artificial perturbation experiment, we showed that the system *could* perform a specific behavior when we introduced a perturbation to component X. However, this observation does not necessarily imply that the system *does* employ a similar perturbation to component X to elicit the same behavior under normal ethological conditions (Adamantidis et al., 2015). Given the degeneracy framework, it is important to appreciate that the existence of *a* solution neither implies its uniqueness nor does it ensure that the solution is employed by the physiological system under standard ethological conditions.

### 4.2 Degeneracy: The way forward

It is important to distinguish between understanding functionality that emerges through interactions between components in an adjacent scale and efforts aimed at causality that leaps across multiple scales. It is clear that assessing interactions between constitutive components in the emergence of function in an adjacent scale have provided invaluable insights in neuroscience. As an example, the question on how different ionic currents at the molecular scale interact to result in the emergence of an action potential in the cellular scale (Hodgkin & Huxley, 1952) has revolutionized several aspects of neuroscience over the past several decades. Even within the framework of degeneracy, the question on whether and how disparate combinations of parameters in a given scale could result in similar functionality in an adjacent scale have provided deep insights into how the nervous system might be solving the robustness problem in the face of variability (Anirudhan & Narayanan, 2015; Dhawale, Smith, & Olveczky, 2017; Foster et al., 1993; Gjorgjieva et al., 2016; Goldman et al., 2001; Katz, 2016; Marder, 2011; Marder & Goaillard, 2006; Marder et al., 2015; Marder & Taylor, 2011; Mukunda & Narayanan, 2017; O'Leary & Marder, 2014; Prinz et al., 2004; Rathour & Narayanan, 2012a; Rathour & Narayanan, 2014; Taylor et al., 2009).

However, causal leaps beyond a single scale of analysis should be treated with extreme caution. For instance, approaches assuming a unique reductionist solution for a behavioral observation will invariably end up in apparently contradictory conclusions about *the* mechanism that mediates behavior. Prominent among the several reasons that result in these apparent contradictions—such as adaptive compensations and animal‐to‐animal variability—is inherent degeneracy, where disparate combinations of components could result in identical behavior in a manner that is dependent on several factors, including behavioral state. The flaws that emerge in an experimental plan to establish causality that leaps multiple scales in a nonlinear dynamical system that expresses degeneracy are obvious from the analysis presented above. Here, it is critical to ask the impossible question on whether we are sure that *nothing else* has changed in neurons (and other cells) of the same brain region or the other, which could be mediating/contributing to the observed behavioral changes *before* declaring a causal one‐to‐one relationship between a molecular/cellular component and behavior.

This is especially important because there are several properties that *emerge* at each jump along the multiscale axis of neuroscience (Figure 1a), and leaps across multiple scales (like genes to behavior) traverses several *emergent* properties owing to innumerable nonlinear processes that exhibit degeneracy. This yields a system that is intractable even at the scale where the perturbations were introduced because of the complex feedback loops spanning several scales that mediate homeostasis and adaptation. Consequently, the outcomes of any perturbation at any scale are critically dependent on several components across scales, the nature of interactions of these components with the perturbation and importantly on the adaptation that is triggered by the perturbation in all these components across scales. Therefore, extreme caution should be exercised in assigning causal one‐to‐one relationship between components (or manifolds) that are several scales apart along the multi‐scale axis (Bennett & Hacker, 2003; Jazayeri & Afraz, 2017; Krakauer et al., 2017; Otchy et al., 2015).

Together, while degeneracy is an invaluable asset to evolution, physiology and behavior in achieving robust functions through several degrees of freedom, it makes the resultant complex system rather intractable. This intractability makes it nearly impossible to achieve the goals of reductionism, where the pursuit has largely been for causal one‐to‐one relationships that leap across several scales. Several thorny debates in the field about apparent contradictions involving different components mediating the same function could be put to rest if this requirement of one‐to‐one relationships is relaxed. Specifically, the ubiquitous expression of degeneracy spanning multiple scales offers an ideal reconciliation to these controversies, through the recognition that the distinct routes to achieve a functional goal are not necessarily contradictory to each other, but are alternate routes that the system might recruit towards accomplishment of the goal. The intense drive to make leaps across multiple scales to establish unique one‐to‐one relationships should instead be replaced by a steadfast recognition for degeneracy as an essential component in physiology, behavior and evolution. This recognition, apart from precluding one‐to‐one relationships, would provide clear warnings in assigning causal relationships that leap across multiple scales and multiple emergent properties. Importantly, this recognition would pave the way for a strong focus on integrative and holistic treatises to neuroscience and behavior, arguments for which have only been growing over the years (Bennett & Hacker, 2003; Edelman & Gally, 2001; Jazayeri & Afraz, 2017; Krakauer et al., 2017; Tononi & Edelman, 1998; Tononi et al., 1998; Tononi, Sporns, & Edelman, 1994; Tytell et al., 2011). Future approaches should recognize that behavior emerges from disparate combinations of tightly cross‐coupled multi‐scale emergent properties, each diverging and converging at each scale of analysis through degeneracy spanning complex parametric and interactional spaces. Large‐scale databases related to neuronal morphology, models and physiology—such as the Allen brain atlas (Sunkin et al., 2013), ICGenealogy (Podlaski et al., 2017), Channelpedia (Ranjan et al., 2011), Neuromorpho (Ascoli, Donohue, & Halavi, 2007), ModelDB (Hines, Morse, Migliore, Carnevale, & Shepherd, 2004) and Neuroelectro (Tripathy, Savitskaya, Burton, Urban, & Gerkin, 2014)—provide ideal tools for such analyses involving large parametric spaces, and could provide critical insights about the role of degeneracy in the emergence of robust brain physiology and its links to behavior.

**Figure 10:**
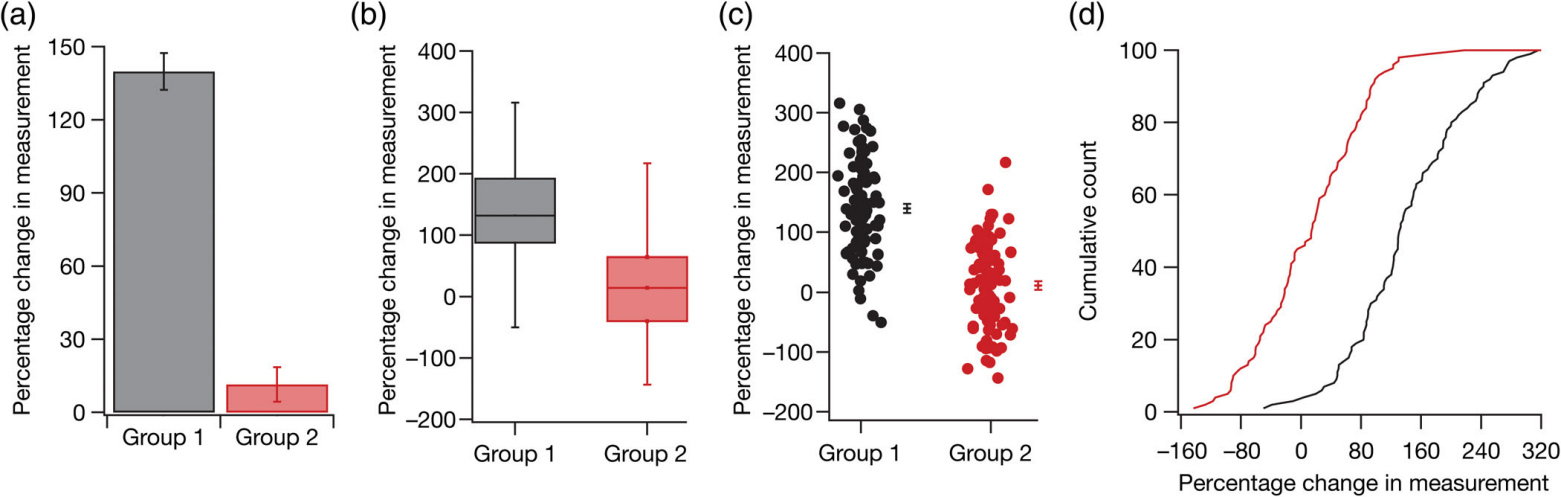
Artificial data from a hypothetical experiment emphasizing the importance of data reporting. (a–d) Artificial data on “percentage change in measurement” belonging to two groups (Group 1 and Group 2) reported as mean with error bars representing *SEM* (a), as median with all four quartiles (b), as a beeswarm plot (c; also showing mean and *SEM*) and as a cumulative histogram of percentage changes (d) [Color figure can be viewed at wileyonlinelibrary.com]

An important aspect in assessing the expression of degeneracy in several functions has to do with how data reporting and interpretation are performed (Marder & Taylor, 2011). Specifically, consider a hypothetical experiment where the percentage change (plasticity) in a measurement was recorded under two different conditions (Figure 10). Let us consider “Group 1” to represent baseline conditions, and “Group 2” to represent a condition where the experiments were performed in the presence of a certain pharmacological agent. Data is typically reported *only* as mean and SEM, and a statistical test is employed to report a significant difference (*p*<.0001 with Student's *t* test in this case) between data collected in “Group 1” and in “Group 2” (Figure 10a). The interpretation of such data is typically derived from the differences in the mean and the *p* value of the statistical test. In the specific case under consideration, the interpretation and the main reporting typically would be that the presence of the pharmacological agent “completely blocks” plasticity.

However, this constitutes a scenario where heterogeneities are hidden under statistics, and *interpreting the average* results in one‐to‐one conclusions. If the data were represented to show the entire distribution (Figure 10b–d), it becomes clear that there were several experiments within each group where the plasticity was similar in the presence or the absence of the pharmacological agent. These representations put to question the earlier interpretation that the pharmacological agent *completely* blocked plasticity, apart from providing the entire dataset and including the heterogeneities that are inherent to the biological system under consideration. This example emphasizes the need to present *all* data rather than providing only the summary statistics, and points to the perils of interpreting experiments based merely on summary statistics (Marder & Taylor, 2011).

Importantly, if the application of another pharmacological agent blocking a different structural component also provides similar data, employing only the summary statistics for interpretation would result in a conflict because two different structural components “completely block” plasticity! However, assessment of the entire data set opens up the possibility for the expression of degeneracy in the system. In this scenario, two different structural components, that are potentially interdependently expressed (Marder & Taylor, 2011), with distinct quantitative contributions (e.g., Figure 5c) resulted in the expression of similar amount of overall plasticity. Therefore, from the perspective of assessing degeneracy, it is extremely important that results of *all* experiments are reported, and that interpretations specifically account for biological heterogeneities (Marder & Taylor, 2011).

## 5 CONCLUSIONS

In this review, we systematically presented lines of evidence pointing to the ubiquitous expression of degeneracy spanning several scales of the mammalian hippocampus. We argued that the framework of degeneracy in an encoding system should not be viewed from the limited perspective of maintaining homeostasis, but should be assessed from the perspective of achieving the twin goals of encoding information and maintaining homeostasis. Within the broad framework of degeneracy, it is extremely important that future studies focus on the fundamental questions on (a) how does the brain change its constituent components towards encoding new information without jeopardizing homeostasis?; and (b) how do homeostatic mechanisms maintain robust function without affecting learning‐induced changes in the brain? Without an effective answer to this overall question on concomitant learning and homeostasis in the face of staggeringly combinatorial complexity, our understanding of the nervous system in terms of its ability to systematically adapt to the environment will remain incomplete. Although the core conclusions on degeneracy reviewed and analyzed here would extend to other mammalian brain regions and functions that they have been implicated in encoding processes, this extrapolation should be preceded by careful assessment of the specifics associated with the constitutive components and specific interactions there. Additionally, although our focus here was on encoding, homeostasis and physiology, it is important that future studies also assess the implications for degeneracy in the emergence of pathological conditions (Edelman & Gally, 2001; O'Leary et al., 2014).

Finally, returning to the distinction between the “structure defines function” and the “form follows function” perspectives, it seems like the distinction also seemingly extends to the methodology that is deemed appropriate for assessing neuronal systems. At one end, a strong emphasis is placed on the requirement for an experimental approach (Buzsaki, 2006):

“The complexity and precision of brain wiring make an experimental approach absolutely necessary. No amount of introspection or algorithmic modeling can help without parallel empirical exploration.”

At the other end, the emphasis, reflecting Richard Feymann's quote “What I cannot create, I do not understand”, is on in silico approaches (Sakmann, 2017):

“At present however, it seems that “What we cannot reconstruct in silico and model we have not understood”.”

Within the degeneracy framework, however, it is starkly evident from existing literature reviewed here that a holistic combination of computational and experimental techniques is indispensible towards understanding structure‐function relationships and the associated complexities (Basak & Narayanan, 2018b; Das et al., 2017; Edelman & Gally, 2001; Foster et al., 1993; Marder, 1998; Marder, 2011; Marder & Goaillard, 2006; Marder & Taylor, 2011; Mishra & Narayanan, 2019; Mittal & Narayanan, 2018; Rathour et al., 2016; Rathour & Narayanan, 2012a; Rathour & Narayanan, 2012b; Rathour & Narayanan, 2014; Sporns et al., 2000; Tononi & Edelman, 1998; Tononi et al., 1998; Tononi et al., 1994; Tononi et al., 1996; Tononi et al., 1999).

Emphasizing the strong links between biology and evolution, Theodosius Dobzhansky had written, “nothing in biology makes sense except in the light of evolution” (Dobzhansky, 1973). Given the ubiquitous prevalence of degeneracy and its strong links to evolution (Edelman & Gally, 2001), it is perhaps apt to add a corollary to this quote and state “nothing in physiology makes sense except in the light of degeneracy”.

## CONFLICT OF INTEREST

The authors declare no conflict of interest.

## Data Availability

Data sharing is not applicable to this article as no new data were created or analyzed in this study. This is a review paper.

## References

[hipo23139-bib-0001] Abbott, L. F. (2003). Balancing homeostasis and learning in neural circuits. Zoology (Jena, Germany), 106(4), 365–371. 10.1078/0944-2006-00133 16351920

[hipo23139-bib-0002] Abbott, L. F. , & LeMasson, G. (1993). Analysis of Neuron Models with Dynamically Regulated Conductances. Neural Computation, 5, 823–842.

[hipo23139-bib-0003] Abbott, L. F. , & Nelson, S. B. (2000). Synaptic plasticity: taming the beast. Nature Neuroscience, 3(Suppl), 1178–1183. 11127835 10.1038/81453

[hipo23139-bib-0004] Abbott, L. F. , & Regehr, W. G. (2004). Synaptic computation. Nature, 431(7010), 796–803. 15483601 10.1038/nature03010

[hipo23139-bib-0005] Abraham, W. C. (2008). Metaplasticity: tuning synapses and networks for plasticity. Nature Reviews. Neuroscience, 9(5), 387. 18401345 10.1038/nrn2356

[hipo23139-bib-0006] Abraham, W. C. , & Bear, M. F. (1996). Metaplasticity: the plasticity of synaptic plasticity. Trends in Neurosciences, 19(4), 126–130. 8658594 10.1016/s0166-2236(96)80018-x

[hipo23139-bib-0007] Abraham, W. C. , Logan, B. , Wolff, A. , & Benuskova, L. (2007). "Heterosynaptic" LTD in the dentate gyrus of anesthetized rat requires homosynaptic activity. Journal of Neurophysiology, 98(2), 1048–1051. 17537906 10.1152/jn.00250.2007

[hipo23139-bib-0008] Abraham, W. C. , Mason‐Parker, S. E. , Bear, M. F. , Webb, S. , & Tate, W. P. (2001). Heterosynaptic metaplasticity in the hippocampus in vivo: a BCM‐like modifiable threshold for LTP. Proceedings of the National Academy of Sciences of the United States of America, 98(19), 10924–10929. 11517323 10.1073/pnas.181342098 PMC58575

[hipo23139-bib-0009] Abraham, W. C. , & Robins, A. (2005). Memory retention‐‐the synaptic stability versus plasticity dilemma. Trends in Neurosciences, 28(2), 73–78. 15667929 10.1016/j.tins.2004.12.003

[hipo23139-bib-0010] Abraham, W. C. , & Tate, W. P. (1997). Metaplasticity: a new vista across the field of synaptic plasticity. Progress in Neurobiology, 52(4), 303–323. 9247968 10.1016/s0301-0082(97)00018-x

[hipo23139-bib-0011] Achard, P. , & De Schutter, E. (2006). Complex parameter landscape for a complex neuron model. PLoS Computational Biology, 2(7), e94. 16848639 10.1371/journal.pcbi.0020094 PMC1513272

[hipo23139-bib-0012] Adamantidis, A. , Arber, S. , Bains, J. S. , Bamberg, E. , Bonci, A. , Buzsaki, G. , et al. (2015). Optogenetics: 10 years after ChR2 in neurons‐‐views from the community. Nature Neuroscience, 18(9), 1202–1212. 26308981 10.1038/nn.4106

[hipo23139-bib-0013] Adelman, J. P. , Maylie, J. , & Sah, P. (2012). Small‐conductance Ca2+‐activated K+ channels: form and function. Annual Review of Physiology, 74, 245–269. 10.1146/annurev-physiol-020911-153336 21942705

[hipo23139-bib-0014] Aghajan, Z. M. , Acharya, L. , Moore, J. J. , Cushman, J. D. , Vuong, C. , & Mehta, M. R. (2015). Impaired spatial selectivity and intact phase precession in two‐dimensional virtual reality. Nature Neuroscience, 18(1), 121–128. 25420065 10.1038/nn.3884

[hipo23139-bib-0015] Ahmed, O. J. , & Mehta, M. R. (2009). The hippocampal rate code: anatomy, physiology and theory. Trends in Neurosciences, 32(6), 329–338. 19406485 10.1016/j.tins.2009.01.009 PMC3066563

[hipo23139-bib-0016] Albantakis, L. , Hintze, A. , Koch, C. , Adami, C. , & Tononi, G. (2014). Evolution of integrated causal structures in animats exposed to environments of increasing complexity. PLoS Computational Biology, 10(12), e1003966. 25521484 10.1371/journal.pcbi.1003966 PMC4270440

[hipo23139-bib-0017] Alberini, C. M. (2009). Transcription factors in long‐term memory and synaptic plasticity. Physiological Reviews, 89(1), 121–145. 19126756 10.1152/physrev.00017.2008 PMC3883056

[hipo23139-bib-0018] Allen, N. J. , & Barres, B. A. (2005). Signaling between glia and neurons: focus on synaptic plasticity. Current Opinion in Neurobiology, 15(5), 542–548. 16144764 10.1016/j.conb.2005.08.006

[hipo23139-bib-0019] Allen, N. J. , & Barres, B. A. (2009). Neuroscience: Glia ‐ more than just brain glue. Nature, 457(7230), 675–677. 19194443 10.1038/457675a

[hipo23139-bib-0020] Amarillo, Y. , De Santiago‐Castillo, J. A. , Dougherty, K. , Maffie, J. , Kwon, E. , Covarrubias, M. , & Rudy, B. (2008). Ternary Kv4.2 channels recapitulate voltage‐dependent inactivation kinetics of A‐type K+ channels in cerebellar granule neurons. The Journal of Physiology, 586(8), 2093–2106. 18276729 10.1113/jphysiol.2007.150540 PMC2465190

[hipo23139-bib-0021] Anastassiou, C. A. , & Koch, C. (2015). Ephaptic coupling to endogenous electric field activity: why bother? Current Opinion in Neurobiology, 31, 95–103. 25265066 10.1016/j.conb.2014.09.002

[hipo23139-bib-0022] Andersen, P. , Morris, R. , Amaral, D. , Bliss, T. , & O'Keefe, J. (2006). The hippocampus book. New York, USA: Oxford University Press.

[hipo23139-bib-0023] Anderson, D. , Mehaffey, W. H. , Iftinca, M. , Rehak, R. , Engbers, J. D. , Hameed, S. , … Turner, R. W. (2010). Regulation of neuronal activity by Cav3‐Kv4 channel signaling complexes. Nature Neuroscience, 13(3), 333–337. 20154682 10.1038/nn.2493

[hipo23139-bib-0024] Anderson, P. , Morris, R. , Amaral, D. , Bliss, T. V. , & O'Keefe, J. (2007). The Hippocampus Book. New York: Oxford University Press.

[hipo23139-bib-0025] Andrasfalvy, B. K. , & Magee, J. C. (2001). Distance‐dependent increase in AMPA receptor number in the dendrites of adult hippocampal CA1 pyramidal neurons. The Journal of Neuroscience, 21(23), 9151–9159. 11717348 10.1523/JNEUROSCI.21-23-09151.2001 PMC6763889

[hipo23139-bib-0026] Andrasfalvy, B. K. , & Mody, I. (2006). Differences between the scaling of miniature IPSCs and EPSCs recorded in the dendrites of CA1 mouse pyramidal neurons. The Journal of Physiology, 576(Pt 1), 191–196. 16887875 10.1113/jphysiol.2006.115428 PMC1995648

[hipo23139-bib-0027] Anirudhan, A. , & Narayanan, R. (2015). Analogous synaptic plasticity profiles emerge from disparate channel combinations. The Journal of Neuroscience, 35(11), 4691–4705. 25788686 10.1523/JNEUROSCI.4223-14.2015 PMC6605137

[hipo23139-bib-0028] Araque, A. (2008). Astrocytes process synaptic information. Neuron Glia Biology, 4(1), 3–10. 19250562 10.1017/S1740925X09000064

[hipo23139-bib-0029] Araque, A. , Carmignoto, G. , Haydon, P. G. , Oliet, S. H. , Robitaille, R. , & Volterra, A. (2014). Gliotransmitters travel in time and space. Neuron, 81(4), 728–739. 24559669 10.1016/j.neuron.2014.02.007 PMC4107238

[hipo23139-bib-0030] Araque, A. , Parpura, V. , Sanzgiri, R. P. , & Haydon, P. G. (1999). Tripartite synapses: glia, the unacknowledged partner. Trends in Neurosciences, 22(5), 208–215. 10322493 10.1016/s0166-2236(98)01349-6

[hipo23139-bib-0031] Armstrong, C. M. , & Bezanilla, F. (1974). Charge movement associated with the opening and closing of the activation gates of the Na channels. The Journal of General Physiology, 63(5), 533–552. 4824995 10.1085/jgp.63.5.533 PMC2203568

[hipo23139-bib-0032] Ascoli, G. A. , Donohue, D. E. , & Halavi, M. (2007). NeuroMorpho.Org: a central resource for neuronal morphologies. The Journal of Neuroscience, 27(35), 9247–9251. 17728438 10.1523/JNEUROSCI.2055-07.2007 PMC6673130

[hipo23139-bib-0033] Ashhad, S. , Johnston, D. , & Narayanan, R. (2015). Activation of InsP3 receptors is sufficient for inducing graded intrinsic plasticity in rat hippocampal pyramidal neurons. Journal of Neurophysiology, 113(7), 2002–2013. 25552640 10.1152/jn.00833.2014 PMC4416566

[hipo23139-bib-0034] Ashhad, S. , & Narayanan, R. (2013). Quantitative interactions between the A‐type K+ current and inositol trisphosphate receptors regulate intraneuronal Ca2+ waves and synaptic plasticity. The Journal of Physiology, 591(Pt 7), 1645–1669. 23283761 10.1113/jphysiol.2012.245688 PMC3624844

[hipo23139-bib-0035] Ashhad, S. , & Narayanan, R. (2016). Active dendrites regulate the impact of gliotransmission on rat hippocampal pyramidal neurons. Proceedings of the National Academy of Sciences of the United States of America, 113(23), E3280–E3289. 27217559 10.1073/pnas.1522180113 PMC4988595

[hipo23139-bib-0036] Ashhad, S. , & Narayanan, R. (2019). Stores, Channels, Glue, and Trees: Active Glial and Active Dendritic Physiology. Molecular Neurobiology, 56(3), 2278–2299. 30014322 10.1007/s12035-018-1223-5 PMC6394607

[hipo23139-bib-0037] Attardo, A. , Fitzgerald, J. E. , & Schnitzer, M. J. (2015). Impermanence of dendritic spines in live adult CA1 hippocampus. Nature, 523(7562), 592–596. 26098371 10.1038/nature14467 PMC4648621

[hipo23139-bib-0038] Atwood, B. K. , Lovinger, D. M. , & Mathur, B. N. (2014). Presynaptic long‐term depression mediated by Gi/o‐coupled receptors. Trends in Neurosciences, 37(11), 663–673. 25160683 10.1016/j.tins.2014.07.010 PMC4252515

[hipo23139-bib-0039] Augustine, G. J. , Santamaria, F. , & Tanaka, K. (2003). Local calcium signaling in neurons. Neuron, 40(2), 331–346. 14556712 10.1016/s0896-6273(03)00639-1

[hipo23139-bib-0040] Baba, A. , Yasui, T. , Fujisawa, S. , Yamada, R. X. , Yamada, M. K. , Nishiyama, N. , … Ikegaya, Y. (2003). Activity‐evoked capacitative Ca2+ entry: implications in synaptic plasticity. The Journal of Neuroscience, 23(21), 7737–7741. 12944501 10.1523/JNEUROSCI.23-21-07737.2003 PMC6740588

[hipo23139-bib-0041] Bading, H. , Ginty, D. D. , & Greenberg, M. E. (1993). Regulation of gene expression in hippocampal neurons by distinct calcium signaling pathways. Science, 260(5105), 181–186. 8097060 10.1126/science.8097060

[hipo23139-bib-0042] Bailey, K. R. , Rustay, N. R. , & Crawley, J. N. (2006). Behavioral phenotyping of transgenic and knockout mice: practical concerns and potential pitfalls. ILAR Journal, 47(2), 124–131. 16547369 10.1093/ilar.47.2.124

[hipo23139-bib-0043] Balch, W. E. , Morimoto, R. I. , Dillin, A. , & Kelly, J. W. (2008). Adapting proteostasis for disease intervention. Science, 319(5865), 916–919. 18276881 10.1126/science.1141448

[hipo23139-bib-0044] Bargmann, C. I. , & Marder, E. (2013). From the connectome to brain function. Nature Methods, 10(6), 483–490. 23866325 10.1038/nmeth.2451

[hipo23139-bib-0045] Barnard, E. A. , Skolnick, P. , Olsen, R. W. , Mohler, H. , Sieghart, W. , Biggio, G. , … Langer, S. Z. (1998). International Union of Pharmacology. XV. Subtypes of gamma‐aminobutyric acidA receptors: classification on the basis of subunit structure and receptor function. Pharmacological Reviews, 50(2), 291–313. 9647870

[hipo23139-bib-0046] Basak, R. , & Narayanan, R. (2018a). Active dendrites regulate the spatiotemporal spread of signaling microdomains. PLoS Computational Biology, 14(11), e1006485. 30383745 10.1371/journal.pcbi.1006485 PMC6233924

[hipo23139-bib-0047] Basak, R. , & Narayanan, R. (2018b). Spatially dispersed synapses yield sharply‐tuned place cell responses through dendritic spike initiation. The Journal of Physiology, 596(17), 4173–4205. 29893405 10.1113/JP275310 PMC6117611

[hipo23139-bib-0048] Basu J , Zaremba JD , Cheung SK , Hitti FL , Zemelman BV , Losonczy A , Siegelbaum SA . 2016 Gating of hippocampal activity, plasticity, and memory by entorhinal cortex long‐range inhibition. Science 351(6269):aaa5694. 10.1126/science.aaa5694 PMC4920085 26744409

[hipo23139-bib-0049] Baumann, N. , & Pham‐Dinh, D. (2001). Biology of oligodendrocyte and myelin in the mammalian central nervous system. Physiological Reviews, 81(2), 871–927. 11274346 10.1152/physrev.2001.81.2.871

[hipo23139-bib-0050] Bazargani, N. , & Attwell, D. (2016). Astrocyte calcium signaling: the third wave. Nature Neuroscience, 19(2), 182–189. 26814587 10.1038/nn.4201

[hipo23139-bib-0051] Bear, M. F. (2003). Bidirectional synaptic plasticity: from theory to reality. Philosophical Transactions of the Royal Society of London. Series B, Biological Sciences, 358(1432), 649–655. 12740110 10.1098/rstb.2002.1255 PMC1693164

[hipo23139-bib-0052] Bear, M. F. , Cooper, L. N. , & Ebner, F. F. (1987). A physiological basis for a theory of synapse modification. Science, 237(4810), 42–48. 3037696 10.1126/science.3037696

[hipo23139-bib-0053] Beck, H. , & Yaari, Y. (2008). Plasticity of intrinsic neuronal properties in CNS disorders. Nature Reviews. Neuroscience, 9(5), 357–369. 18425090 10.1038/nrn2371

[hipo23139-bib-0054] Beining M , Mongiat LA , Schwarzacher SW , Cuntz H , Jedlicka P . 2017 T2N as a new tool for robust electrophysiological modeling demonstrated for mature and adult‐born dentate granule cells. Elife 6. 10.7554/eLife.26517 PMC5737656 29165247

[hipo23139-bib-0055] Bellman, R. E. (1957). Dynamic programming. Princeton, NJ: Princeton University Press.

[hipo23139-bib-0056] Bennett, M. R. , & Hacker, P. M. S. (2003). Philosophical Foundations of Neuroscience. Malden, MA: Wiley‐Blackwell.

[hipo23139-bib-0057] Benuskova, L. , & Abraham, W. C. (2007). STDP rule endowed with the BCM sliding threshold accounts for hippocampal heterosynaptic plasticity. Journal of Computational Neuroscience, 22(2), 129–133. 17053995 10.1007/s10827-006-0002-x

[hipo23139-bib-0058] Bernard, C. , Shah, M. , & Johnston, D. (2007). Dendrites and disease In Stuart G., Spruston N., & Hausser M. (Eds.), Dendrites (2 nd ed.). New York: Oxford University Press.

[hipo23139-bib-0059] Berridge, M. J. (1998). Neuronal calcium signaling. Neuron, 21(1), 13–26. 9697848 10.1016/s0896-6273(00)80510-3

[hipo23139-bib-0060] Berridge, M. J. (2002). The endoplasmic reticulum: a multifunctional signaling organelle. Cell Calcium, 32(5‐6), 235–249. 12543086 10.1016/s0143416002001823

[hipo23139-bib-0061] Berridge, M. J. (2006). Calcium microdomains: organization and function. Cell Calcium, 40(5‐6), 405–412. 17030366 10.1016/j.ceca.2006.09.002

[hipo23139-bib-0062] Berridge, M. J. , Lipp, P. , & Bootman, M. D. (2000). The versatility and universality of calcium signalling. Nature Reviews. Molecular Cell Biology, 1(1), 11–21. 11413485 10.1038/35036035

[hipo23139-bib-0063] Bezprozvanny, I. , Watras, J. , & Ehrlich, B. E. (1991). Bell‐shaped calcium‐response curves of Ins(1,4,5)P3‐ and calcium‐gated channels from endoplasmic reticulum of cerebellum. Nature, 351(6329), 751–754. 1648178 10.1038/351751a0

[hipo23139-bib-0064] Bhalla, U. S. (2014). Multiscale modeling and synaptic plasticity. Progress in Molecular Biology and Translational Science, 123, 351–386. 24560151 10.1016/B978-0-12-397897-4.00012-7

[hipo23139-bib-0065] Bhalla, U. S. , & Iyengar, R. (1999). Emergent properties of networks of biological signaling pathways. Science, 283(5400), 381–387. 9888852 10.1126/science.283.5400.381

[hipo23139-bib-0066] Bi, G. Q. , & Poo, M. M. (1998). Synaptic modifications in cultured hippocampal neurons: dependence on spike timing, synaptic strength, and postsynaptic cell type. The Journal of Neuroscience, 18(24), 10464–10472. 9852584 10.1523/JNEUROSCI.18-24-10464.1998 PMC6793365

[hipo23139-bib-0067] Bickle, J. (2015). Marr and reductionism. Topics in Cognitive Science, 7(2), 299–311. 25772159 10.1111/tops.12134

[hipo23139-bib-0068] Biel, M. , Wahl‐Schott, C. , Michalakis, S. , & Zong, X. (2009). Hyperpolarization‐activated cation channels: from genes to function. Physiological Reviews, 89(3), 847–885. 19584315 10.1152/physrev.00029.2008

[hipo23139-bib-0069] Bienenstock, E. L. , Cooper, L. N. , & Munro, P. W. (1982). Theory for the development of neuron selectivity: orientation specificity and binocular interaction in visual cortex. The Journal of Neuroscience, 2(1), 32–48. 7054394 10.1523/JNEUROSCI.02-01-00032.1982 PMC6564292

[hipo23139-bib-0070] Bird, C. M. , & Burgess, N. (2008). The hippocampus and memory: insights from spatial processing. Nature Reviews. Neuroscience, 9(3), 182–194. 18270514 10.1038/nrn2335

[hipo23139-bib-0071] Birnbaum, S. G. , Varga, A. W. , Yuan, L. L. , Anderson, A. E. , Sweatt, J. D. , & Schrader, L. A. (2004). Structure and function of Kv4‐family transient potassium channels. Physiological Reviews, 84(3), 803–833. 15269337 10.1152/physrev.00039.2003

[hipo23139-bib-0072] Bittner, K. C. , Grienberger, C. , Vaidya, S. P. , Milstein, A. D. , Macklin, J. J. , Suh, J. , … Magee, J. C. (2015). Conjunctive input processing drives feature selectivity in hippocampal CA1 neurons. Nature Neuroscience, 18(8), 1133–1142. 26167906 10.1038/nn.4062 PMC4888374

[hipo23139-bib-0073] Bittner, K. C. , Milstein, A. D. , Grienberger, C. , Romani, S. , & Magee, J. C. (2017). Behavioral time scale synaptic plasticity underlies CA1 place fields. Science, 357(6355), 1033–1036. 28883072 10.1126/science.aan3846 PMC7289271

[hipo23139-bib-0074] Bliss, T. V. , & Collingridge, G. L. (1993). A synaptic model of memory: long‐term potentiation in the hippocampus. Nature, 361(6407), 31–39. 8421494 10.1038/361031a0

[hipo23139-bib-0075] Bliss, T. V. , & Gardner‐Medwin, A. R. (1973). Long‐lasting potentiation of synaptic transmission in the dentate area of the unanaestetized rabbit following stimulation of the perforant path. The Journal of Physiology, 232(2), 357–374. 4727085 10.1113/jphysiol.1973.sp010274 PMC1350459

[hipo23139-bib-0076] Bliss, T. V. , & Lomo, T. (1973). Long‐lasting potentiation of synaptic transmission in the dentate area of the anaesthetized rabbit following stimulation of the perforant path. The Journal of Physiology, 232(2), 331–356. 4727084 10.1113/jphysiol.1973.sp010273 PMC1350458

[hipo23139-bib-0077] Bouchard, R. , Pattarini, R. , & Geiger, J. D. (2003). Presence and functional significance of presynaptic ryanodine receptors. Progress in Neurobiology, 69(6), 391–418. 12880633 10.1016/s0301-0082(03)00053-4

[hipo23139-bib-0078] Brager, D. H. , & Johnston, D. (2007). Plasticity of intrinsic excitability during long‐term depression is mediated through mGluR‐dependent changes in $I_{\rm h}$ in hippocampal CA1 pyramidal neurons. The Journal of Neuroscience, 27, 13926–13937. 18094230 10.1523/JNEUROSCI.3520-07.2007 PMC6673524

[hipo23139-bib-0079] Brager, D. H. , & Johnston, D. (2014). Channelopathies and dendritic dysfunction in fragile X syndrome. Brain Research Bulletin, 103C, 11–17. 10.1016/j.brainresbull.2014.01.002 PMC4049233 24462643

[hipo23139-bib-0080] Brager, D. H. , Lewis, A. S. , Chetkovich, D. M. , & Johnston, D. (2013). Short‐ and long‐term plasticity in CA1 neurons from mice lacking h‐channel auxiliary subunit TRIP8b. Journal of Neurophysiology, 110(10), 2350–2357. 23966674 10.1152/jn.00218.2013 PMC3841871

[hipo23139-bib-0081] Branco, T. , & Hausser, M. (2010). The single dendritic branch as a fundamental functional unit in the nervous system. Current Opinion in Neurobiology, 20(4), 494–502. 20800473 10.1016/j.conb.2010.07.009

[hipo23139-bib-0082] Branco, T. , & Hausser, M. (2011). Synaptic integration gradients in single cortical pyramidal cell dendrites. Neuron, 69(5), 885–892. 21382549 10.1016/j.neuron.2011.02.006 PMC6420135

[hipo23139-bib-0083] Budde, T. , Meuth, S. , & Pape, H. C. (2002). Calcium‐dependent inactivation of neuronal calcium channels. Nature Reviews. Neuroscience, 3(11), 873–883. 12415295 10.1038/nrn959

[hipo23139-bib-0084] Bunsey, M. , & Eichenbaum, H. (1996). Conservation of hippocampal memory function in rats and humans. Nature, 379(6562), 255–257. 8538790 10.1038/379255a0

[hipo23139-bib-0085] Buzsaki, G. (1986). Hippocampal sharp waves: their origin and significance. Brain Research, 398(2), 242–252. 3026567 10.1016/0006-8993(86)91483-6

[hipo23139-bib-0086] Buzsaki, G. (1989). Two‐stage model of memory trace formation: a role for "noisy" brain states. Neuroscience, 31(3), 551–570. 2687720 10.1016/0306-4522(89)90423-5

[hipo23139-bib-0087] Buzsaki, G. (2002). Theta oscillations in the hippocampus. Neuron, 33(3), 325–340. 11832222 10.1016/s0896-6273(02)00586-x

[hipo23139-bib-0088] Buzsaki, G. (2006). Rhythms of the brain. New York: Oxford University Press.

[hipo23139-bib-0089] Buzsaki, G. (2010). Neural syntax: cell assemblies, synapsembles, and readers. Neuron, 68(3), 362–385. 21040841 10.1016/j.neuron.2010.09.023 PMC3005627

[hipo23139-bib-0090] Buzsaki, G. (2015). Hippocampal sharp wave‐ripple: A cognitive biomarker for episodic memory and planning. Hippocampus, 25(10), 1073–1188. 26135716 10.1002/hipo.22488 PMC4648295

[hipo23139-bib-0091] Buzsaki, G. , Anastassiou, C. A. , & Koch, C. (2012). The origin of extracellular fields and currents‐‐EEG, ECoG, LFP and spikes. Nature Reviews. Neuroscience, 13(6), 407–420. 22595786 10.1038/nrn3241 PMC4907333

[hipo23139-bib-0092] Buzsaki, G. , Logothetis, N. , & Singer, W. (2013). Scaling brain size, keeping timing: evolutionary preservation of brain rhythms. Neuron, 80(3), 751–764. 24183025 10.1016/j.neuron.2013.10.002 PMC4009705

[hipo23139-bib-0093] Buzsaki, G. , & Moser, E. I. (2013). Memory, navigation and theta rhythm in the hippocampal‐entorhinal system. Nature Neuroscience, 16(2), 130–138. 23354386 10.1038/nn.3304 PMC4079500

[hipo23139-bib-0094] Buzsaki, G. , & Wang, X. J. (2012). Mechanisms of gamma oscillations. Annual Review of Neuroscience, 35, 203–225. 10.1146/annurev-neuro-062111-150444 PMC4049541 22443509

[hipo23139-bib-0095] Cantrell, A. R. , & Catterall, W. A. (2001). Neuromodulation of Na+ channels: an unexpected form of cellular plasticity. Nature Reviews. Neuroscience, 2(6), 397–407. 11389473 10.1038/35077553

[hipo23139-bib-0096] Carlson, J. M. , & Doyle, J. (2002). Complexity and robustness. Proceedings of the National Academy of Sciences of the United States of America, 99(Suppl 1), 2538–2545. 11875207 10.1073/pnas.012582499 PMC128573

[hipo23139-bib-0097] Catterall, W. A. (1993). Structure and function of voltage‐gated ion channels. Trends in Neurosciences, 16(12), 500–506. 7509519 10.1016/0166-2236(93)90193-p

[hipo23139-bib-0098] Catterall, W. A. (1995). Structure and function of voltage‐gated ion channels. Annual Review of Biochemistry, 64, 493–531. 10.1146/annurev.bi.64.070195.002425 7574491

[hipo23139-bib-0099] Chen, C. C. , Lu, J. , & Zuo, Y. (2014). Spatiotemporal dynamics of dendritic spines in the living brain. Frontiers in Neuroanatomy, 8, 28. 24847214 10.3389/fnana.2014.00028 PMC4023020

[hipo23139-bib-0100] Chen, X. , Yuan, L. L. , Zhao, C. , Birnbaum, S. G. , Frick, A. , Jung, W. E. , … Johnston, D. (2006). Deletion of Kv4.2 gene eliminates dendritic A‐type K+ current and enhances induction of long‐term potentiation in hippocampal CA1 pyramidal neurons. The Journal of Neuroscience, 26(47), 12143–12151. 17122039 10.1523/JNEUROSCI.2667-06.2006 PMC6675426

[hipo23139-bib-0101] Cheong, R. , Rhee, A. , Wang, C. J. , Nemenman, I. , & Levchenko, A. (2011). Information transduction capacity of noisy biochemical signaling networks. Science, 334(6054), 354–358. 21921160 10.1126/science.1204553 PMC3895446

[hipo23139-bib-0102] Chevaleyre, V. , Takahashi, K. A. , & Castillo, P. E. (2006). Endocannabinoid‐mediated synaptic plasticity in the CNS. Annual Review of Neuroscience, 29, 37–76. 10.1146/annurev.neuro.29.051605.112834 16776579

[hipo23139-bib-0103] Choe, C. U. , & Ehrlich, B. E. (2006). The inositol 1,4,5‐trisphosphate receptor (IP3R) and its regulators: sometimes good and sometimes bad teamwork. Science's STKE, 2006(363), re15. 10.1126/stke.3632006re15 17132820

[hipo23139-bib-0104] Christie, B. R. , Kerr, D. S. , & Abraham, W. C. (1994). Flip side of synaptic plasticity: long‐term depression mechanisms in the hippocampus. Hippocampus, 4(2), 127–135. 7951687 10.1002/hipo.450040203

[hipo23139-bib-0105] Christie, B. R. , Magee, J. C. , & Johnston, D. (1996). The role of dendritic action potentials and Ca2+ influx in the induction of homosynaptic long‐term depression in hippocampal CA1 pyramidal neurons. Learning & Memory, 3(2‐3), 160–169. 10456086 10.1101/lm.3.2-3.160

[hipo23139-bib-0106] Christie, B. R. , Schexnayder, L. K. , & Johnston, D. (1997). Contribution of voltage‐gated Ca2+ channels to homosynaptic long‐term depression in the CA1 region in vitro. Journal of Neurophysiology, 77(3), 1651–1655. 9084630 10.1152/jn.1997.77.3.1651

[hipo23139-bib-0107] Chung, H. J. , Ge, W. P. , Qian, X. , Wiser, O. , Jan, Y. N. , & Jan, L. Y. (2009). G protein‐activated inwardly rectifying potassium channels mediate depotentiation of long‐term potentiation. Proceedings of the National Academy of Sciences of the United States of America, 106(2), 635–640. 19118199 10.1073/pnas.0811685106 PMC2613041

[hipo23139-bib-0108] Chung, H. J. , Qian, X. , Ehlers, M. , Jan, Y. N. , & Jan, L. Y. (2009). Neuronal activity regulates phosphorylation‐dependent surface delivery of G protein‐activated inwardly rectifying potassium channels. Proceedings of the National Academy of Sciences of the United States of America, 106(2), 629–634. 19118198 10.1073/pnas.0811615106 PMC2613039

[hipo23139-bib-0109] Churchland, P. , & Sejnowski, T. (1992). The Computational Brain. Cambridge, MA: MIT Press.

[hipo23139-bib-0110] Churchland, P. S. , & Sejnowski, T. J. (1988). Perspectives on cognitive neuroscience. Science, 242(4879), 741–745. 3055294 10.1126/science.3055294

[hipo23139-bib-0111] Clemens, A. M. , & Johnston, D. (2014). Age‐ and location‐dependent differences in store depletion‐induced h‐channel plasticity in hippocampal pyramidal neurons. Journal of Neurophysiology, 111(6), 1369–1382. 24381027 10.1152/jn.00839.2013 PMC3949316

[hipo23139-bib-0112] Cole, K. S. (1968). Membranes, ions and impulses: A chapter of classical biophysics. Berkeley: University of California, Berkeley press.

[hipo23139-bib-0113] Colgin, L. L. (2013). Mechanisms and Functions of Theta Rhythms. Annual Review of Neuroscience, 36(36), 295–312. 10.1146/annurev-neuro-062012-170330 23724998

[hipo23139-bib-0114] Colgin, L. L. (2016). Rhythms of the hippocampal network. Nature Reviews. Neuroscience, 17(4), 239–249. 26961163 10.1038/nrn.2016.21 PMC4890574

[hipo23139-bib-0115] Colgin, L. L. , & Moser, E. I. (2010). Gamma oscillations in the hippocampus. Physiology (Bethesda), 25(5), 319–329. 20940437 10.1152/physiol.00021.2010

[hipo23139-bib-0116] Collingridge, G. L. , & Bliss, T. V. P. (1987). NMDA receptors ‐ their role in long‐term potentiation. Trends in Neurosciences, 10(7), 288–293.

[hipo23139-bib-0117] Collingridge, G. L. , Kehl, S. J. , & McLennan, H. (1983). Excitatory amino acids in synaptic transmission in the Schaffer collateral‐commissural pathway of the rat hippocampus. The Journal of Physiology, 334, 33–46. 6306230 10.1113/jphysiol.1983.sp014478 PMC1197298

[hipo23139-bib-0118] Cooper, L. N. , & Bear, M. F. (2012). The BCM theory of synapse modification at 30: interaction of theory with experiment. Nature Reviews. Neuroscience, 13(11), 798–810. 23080416 10.1038/nrn3353

[hipo23139-bib-0119] Cooper, L. N. , Intrator, N. , Blais, B. S. , & Shouval, H. Z. (2004). Theory of cortical plasticity. Singapore: World Scientific Publishing Company.

[hipo23139-bib-0120] Csicsvari, J. , Jamieson, B. , Wise, K. D. , & Buzsaki, G. (2003). Mechanisms of gamma oscillations in the hippocampus of the behaving rat. Neuron, 37(2), 311–322. 12546825 10.1016/s0896-6273(02)01169-8

[hipo23139-bib-0121] Cusdin, F. S. , Clare, J. J. , & Jackson, A. P. (2008). Trafficking and cellular distribution of voltage‐gated sodium channels. Traffic, 9(1), 17–26. 17988224 10.1111/j.1600-0854.2007.00673.x

[hipo23139-bib-0122] Dam, A. M. (1980). Epilepsy and neuron loss in the hippocampus. Epilepsia, 21(6), 617–629. 6777154 10.1111/j.1528-1157.1980.tb04315.x

[hipo23139-bib-0123] Dan, Y. , & Poo, M. M. (2006). Spike timing‐dependent plasticity: from synapse to perception. Physiological Reviews, 86(3), 1033–1048. 16816145 10.1152/physrev.00030.2005

[hipo23139-bib-0124] Danielson, N. B. , Zaremba, J. D. , Kaifosh, P. , Bowler, J. , Ladow, M. , & Losonczy, A. (2016). Sublayer‐Specific Coding Dynamics during Spatial Navigation and Learning in Hippocampal Area CA1. Neuron, 91(3), 652–665. 27397517 10.1016/j.neuron.2016.06.020 PMC4975984

[hipo23139-bib-0125] Das, A. , & Narayanan, R. (2014). Active dendrites regulate spectral selectivity in location‐dependent spike initiation dynamics of hippocampal model neurons. The Journal of Neuroscience, 34(4), 1195–1211. 24453312 10.1523/JNEUROSCI.3203-13.2014 PMC6705308

[hipo23139-bib-0126] Das, A. , & Narayanan, R. (2015). Active dendrites mediate stratified gamma‐range coincidence detection in hippocampal model neurons. The Journal of Physiology, 593(16), 3549–3576. 26018187 10.1113/JP270688 PMC4560584

[hipo23139-bib-0127] Das, A. , & Narayanan, R. (2017). Theta‐frequency selectivity in the somatic spike triggered average of rat hippocampal pyramidal neurons is dependent on HCN channels. Journal of Neurophysiology, 118(4), 2251–2266. 28768741 10.1152/jn.00356.2017 PMC5626898

[hipo23139-bib-0128] Das, A. , Rathour, R. K. , & Narayanan, R. (2017). Strings on a Violin: Location Dependence of Frequency Tuning in Active Dendrites. Frontiers in Cellular Neuroscience, 11, 72. 28348519 10.3389/fncel.2017.00072 PMC5346355

[hipo23139-bib-0129] de Lanerolle, N. C. , Kim, J. H. , Robbins, R. J. , & Spencer, D. D. (1989). Hippocampal interneuron loss and plasticity in human temporal lobe epilepsy. Brain Research, 495(2), 387–395. 2569920 10.1016/0006-8993(89)90234-5

[hipo23139-bib-0130] De Pitta, M. , Volman, V. , Berry, H. , & Ben‐Jacob, E. (2011). A tale of two stories: astrocyte regulation of synaptic depression and facilitation. PLoS Computational Biology, 7(12), e1002293. 22162957 10.1371/journal.pcbi.1002293 PMC3228793

[hipo23139-bib-0131] Deitmer, J. W. , McCarthy, K. D. , Scemes, E. , & Giaume, C. (2006). Information processing and transmission in glia: calcium signaling and transmitter release. Glia, 54(7), 639–641. 17006933 10.1002/glia.20428

[hipo23139-bib-0132] Derdikman, D. , & Moser, E. I. (2010). A manifold of spatial maps in the brain. Trends in Cognitive Sciences, 14(12), 561–569. 20951631 10.1016/j.tics.2010.09.004

[hipo23139-bib-0133] Derkach, V. , Barria, A. , & Soderling, T. R. (1999). Ca2+/calmodulin‐kinase II enhances channel conductance of alpha‐amino‐3‐hydroxy‐5‐methyl‐4‐isoxazolepropionate type glutamate receptors. Proceedings of the National Academy of Sciences of the United States of America, 96(6), 3269–3274. 10077673 10.1073/pnas.96.6.3269 PMC15931

[hipo23139-bib-0134] Derkach, V. A. , Oh, M. C. , Guire, E. S. , & Soderling, T. R. (2007). Regulatory mechanisms of AMPA receptors in synaptic plasticity. Nature Reviews. Neuroscience, 8(2), 101–113. 17237803 10.1038/nrn2055

[hipo23139-bib-0135] Desai, N. S. , Cudmore, R. H. , Nelson, S. B. , & Turrigiano, G. G. (2002). Critical periods for experience‐dependent synaptic scaling in visual cortex. Nature Neuroscience, 5(8), 783–789. 12080341 10.1038/nn878

[hipo23139-bib-0136] Desai, N. S. , Rutherford, L. C. , & Turrigiano, G. G. (1999). Plasticity in the intrinsic excitability of cortical pyramidal neurons. Nature Neuroscience, 2(6), 515–520. 10448215 10.1038/9165

[hipo23139-bib-0137] Dhawale, A. K. , Smith, M. A. , & Olveczky, B. P. (2017). The Role of Variability in Motor Learning. Annual Review of Neuroscience, 40, 479–498. 10.1146/annurev-neuro-072116-031548 PMC6091866 28489490

[hipo23139-bib-0138] Dhupia, N. , Rathour, R. K. , & Narayanan, R. (2015). Dendritic atrophy constricts functional maps in resonance and impedance properties of hippocampal model neurons. Frontiers in Cellular Neuroscience, 8, 456. 25628537 10.3389/fncel.2014.00456 PMC4289900

[hipo23139-bib-0139] Diesmann, M. , Gewaltig, M. O. , & Aertsen, A. (1999). Stable propagation of synchronous spiking in cortical neural networks. Nature, 402(6761), 529–533. 10591212 10.1038/990101

[hipo23139-bib-0140] Dingledine, R. , Borges, K. , Bowie, D. , & Traynelis, S. F. (1999). The glutamate receptor ion channels. Pharmacological Reviews, 51(1), 7–61. 10049997

[hipo23139-bib-0141] Dittman, J. S. , Kreitzer, A. C. , & Regehr, W. G. (2000). Interplay between facilitation, depression, and residual calcium at three presynaptic terminals. The Journal of Neuroscience, 20(4), 1374–1385. 10662828 10.1523/JNEUROSCI.20-04-01374.2000 PMC6772383

[hipo23139-bib-0142] Dittmer, P. J. , Wild, A. R. , Dell'Acqua, M. L. , & Sather, W. A. (2017). STIM1 Ca(2+) Sensor Control of L‐type Ca(2+)‐Channel‐Dependent Dendritic Spine Structural Plasticity and Nuclear Signaling. Cell Reports, 19(2), 321–334. 28402855 10.1016/j.celrep.2017.03.056 PMC5451256

[hipo23139-bib-0143] Dobzhansky, T. (1973). Nothing in Biology Makes Sense except in the Light of Evolution. The American Biology Teacher, 35(3), 125–129.

[hipo23139-bib-0144] Dolmetsch, R. (2003). Excitation‐transcription coupling: signaling by ion channels to the nucleus. Science's STKE, 2003(166), PE4. 10.1126/stke.2003.166.pe4 12538881

[hipo23139-bib-0145] Dougherty, K. A. , Islam, T. , & Johnston, D. (2012). Intrinsic excitability of CA1 pyramidal neurones from the rat dorsal and ventral hippocampus. The Journal of Physiology, 590(Pt 22), 5707–5722. 22988138 10.1113/jphysiol.2012.242693 PMC3528986

[hipo23139-bib-0146] Dougherty, K. A. , Nicholson, D. A. , Diaz, L. , Buss, E. W. , Neuman, K. M. , Chetkovich, D. M. , & Johnston, D. (2013). Differential expression of HCN subunits alters voltage‐dependent gating of h‐channels in CA1 pyramidal neurons from dorsal and ventral hippocampus. Journal of Neurophysiology, 109(7), 1940–1953. 23324324 10.1152/jn.00010.2013 PMC3628004

[hipo23139-bib-0147] Drion, G. , O'Leary, T. , & Marder, E. (2015). Ion channel degeneracy enables robust and tunable neuronal firing rates. Proceedings of the National Academy of Sciences of the United States of America, 112(38), E5361–E5370. 26354124 10.1073/pnas.1516400112 PMC4586887

[hipo23139-bib-0148] Dudek, S. M. , & Bear, M. F. (1992). Homosynaptic long‐term depression in area CA1 of hippocampus and effects of N‐methyl‐D‐aspartate receptor blockade. Proceedings of the National Academy of Sciences of the United States of America, 89(10), 4363–4367. 1350090 10.1073/pnas.89.10.4363 PMC49082

[hipo23139-bib-0149] Dudek, S. M. , & Bear, M. F. (1993). Bidirectional long‐term modification of synaptic effectiveness in the adult and immature hippocampus. The Journal of Neuroscience, 13(7), 2910–2918. 8331379 10.1523/JNEUROSCI.13-07-02910.1993 PMC6576673

[hipo23139-bib-0150] Dudman, J. T. , Tsay, D. , & Siegelbaum, S. A. (2007). A role for synaptic inputs at distal dendrites: instructive signals for hippocampal long‐term plasticity. Neuron, 56(5), 866–879. 18054862 10.1016/j.neuron.2007.10.020 PMC2179894

[hipo23139-bib-0151] Edelman, G. M. , & Gally, J. A. (2001). Degeneracy and complexity in biological systems. Proceedings of the National Academy of Sciences of the United States of America, 98(24), 13763–13768. 11698650 10.1073/pnas.231499798 PMC61115

[hipo23139-bib-0152] Eichenbaum, H. (2012). The Cognitive Neuroscience of Memory: An Introduction. New York, NY: Oxford University Press.

[hipo23139-bib-0153] Einevoll, G. T. , Kayser, C. , Logothetis, N. K. , & Panzeri, S. (2013). Modelling and analysis of local field potentials for studying the function of cortical circuits. Nature Reviews. Neuroscience, 14(11), 770–785. 24135696 10.1038/nrn3599

[hipo23139-bib-0154] Emoto, K. (2011). Dendrite remodeling in development and disease. Development, Growth & Differentiation, 53(3), 277–286. 10.1111/j.1440-169X.2010.01242.x 21492146

[hipo23139-bib-0155] Emptage, N. J. , Reid, C. A. , & Fine, A. (2001). Calcium stores in hippocampal synaptic boutons mediate short‐term plasticity, store‐operated Ca2+ entry, and spontaneous transmitter release. Neuron, 29(1), 197–208. 11182091 10.1016/s0896-6273(01)00190-8

[hipo23139-bib-0156] Engel, A. K. , Fries, P. , & Singer, W. (2001). Dynamic predictions: oscillations and synchrony in top‐down processing. Nature Reviews. Neuroscience, 2(10), 704–716. 11584308 10.1038/35094565

[hipo23139-bib-0157] Engel, A. K. , & Singer, W. (2001). Temporal binding and the neural correlates of sensory awareness. Trends in Cognitive Sciences, 5(1), 16–25. 11164732 10.1016/s1364-6613(00)01568-0

[hipo23139-bib-0158] Engert, F. , & Bonhoeffer, T. (1999). Dendritic spine changes associated with hippocampal long‐term synaptic plasticity. Nature, 399(6731), 66–70. 10331391 10.1038/19978

[hipo23139-bib-0159] English, D. F. , Peyrache, A. , Stark, E. , Roux, L. , Vallentin, D. , Long, M. A. , & Buzsaki, G. (2014). Excitation and inhibition compete to control spiking during hippocampal ripples: intracellular study in behaving mice. The Journal of Neuroscience, 34(49), 16509–16517. 25471587 10.1523/JNEUROSCI.2600-14.2014 PMC4252557

[hipo23139-bib-0160] English, J. D. , & Sweatt, J. D. (1997). A requirement for the mitogen‐activated protein kinase cascade in hippocampal long term potentiation. The Journal of Biological Chemistry, 272(31), 19103–19106. 9235897 10.1074/jbc.272.31.19103

[hipo23139-bib-0161] Fan, Y. , Fricker, D. , Brager, D. H. , Chen, X. , Lu, H. C. , Chitwood, R. A. , & Johnston, D. (2005). Activity‐dependent decrease of excitability in rat hippocampal neurons through increases in I(h). Nature Neuroscience, 8(11), 1542–1551. 16234810 10.1038/nn1568

[hipo23139-bib-0162] Fields, R. D. (2010). Neuroscience. Change in the brain's white matter. Science, 330(6005), 768–769. 21051624 10.1126/science.1199139 PMC3201847

[hipo23139-bib-0163] Fields, R. D. , & Stevens‐Graham, B. (2002). New insights into neuron‐glia communication. Science, 298(5593), 556–562. 12386325 10.1126/science.298.5593.556 PMC1226318

[hipo23139-bib-0164] Finch, E. A. , Turner, T. J. , & Goldin, S. M. (1991). Calcium as a coagonist of inositol 1,4,5‐trisphosphate‐induced calcium release. Science, 252(5004), 443–446. 2017683 10.1126/science.2017683

[hipo23139-bib-0165] Fioravante, D. , & Regehr, W. G. (2011). Short‐term forms of presynaptic plasticity. Current Opinion in Neurobiology, 21(2), 269–274. 21353526 10.1016/j.conb.2011.02.003 PMC3599780

[hipo23139-bib-0166] Fortune, E. S. , & Rose, G. J. (2001). Short‐term synaptic plasticity as a temporal filter. Trends in Neurosciences, 24(7), 381–385. 11410267 10.1016/s0166-2236(00)01835-x

[hipo23139-bib-0167] Foster, W. R. , Ungar, L. H. , & Schwaber, J. S. (1993). Significance of conductances in Hodgkin‐Huxley models. Journal of Neurophysiology, 70(6), 2502–2518. 7509859 10.1152/jn.1993.70.6.2502

[hipo23139-bib-0168] Freund, T. F. , & Buzsaki, G. (1996). Interneurons of the hippocampus. Hippocampus, 6(4), 347–470. 8915675 10.1002/(SICI)1098-1063(1996)6:4<347::AID-HIPO1>3.0.CO;2-I

[hipo23139-bib-0169] Frey, U. , Huang, Y. Y. , & Kandel, E. R. (1993). Effects of cAMP simulate a late stage of LTP in hippocampal CA1 neurons. Science, 260(5114), 1661–1664. 8389057 10.1126/science.8389057

[hipo23139-bib-0170] Frick, A. , & Johnston, D. (2005). Plasticity of dendritic excitability. Journal of Neurobiology, 64(1), 100–115. 15884001 10.1002/neu.20148

[hipo23139-bib-0171] Frick, A. , Magee, J. , & Johnston, D. (2004). LTP is accompanied by an enhanced local excitability of pyramidal neuron dendrites. Nature Neuroscience, 7(2), 126–135. 14730307 10.1038/nn1178

[hipo23139-bib-0172] Frick, A. , Magee, J. , Koester, H. J. , Migliore, M. , & Johnston, D. (2003). Normalization of Ca2+ signals by small oblique dendrites of CA1 pyramidal neurons. The Journal of Neuroscience, 23(8), 3243–3250. 12716931 10.1523/JNEUROSCI.23-08-03243.2003 PMC6742323

[hipo23139-bib-0173] Fries, P. , Nikolic, D. , & Singer, W. (2007). The gamma cycle. Trends in Neurosciences, 30(7), 309–316. 17555828 10.1016/j.tins.2007.05.005

[hipo23139-bib-0174] Gallistel, C. R. (2017). The Coding Question. Trends in Cognitive Sciences, 21(7), 498–508. 28522379 10.1016/j.tics.2017.04.012

[hipo23139-bib-0175] Garcia‐Alvarez, G. , Shetty, M. S. , Lu, B. , Yap, K. A. , Oh‐Hora, M. , Sajikumar, S. , … Fivaz, M. (2015). Impaired spatial memory and enhanced long‐term potentiation in mice with forebrain‐specific ablation of the Stim genes. Frontiers in Behavioral Neuroscience, 9, 180. 26236206 10.3389/fnbeh.2015.00180 PMC4500926

[hipo23139-bib-0176] Gasparini, S. , & DiFrancesco, D. (1997). Action of the hyperpolarization‐activated current (Ih) blocker ZD 7288 in hippocampal CA1 neurons. Pflügers Archiv, 435(1), 99–106. 9359908 10.1007/s004240050488

[hipo23139-bib-0177] Gasparini, S. , & Magee, J. (2002). Phosphorylation‐dependent differences in the activation properties of distal and proximal dendritic Na+ channels in rat CA1 hippocampal neurons. The Journal of Physiology, 541(Pt 3), 665–672. 12068031 10.1113/jphysiol.2002.020503 PMC2290356

[hipo23139-bib-0178] Geisler, C. , Diba, K. , Pastalkova, E. , Mizuseki, K. , Royer, S. , & Buzsaki, G. (2010). Temporal delays among place cells determine the frequency of population theta oscillations in the hippocampus. Proceedings of the National Academy of Sciences of the United States of America, 107(17), 7957–7962. 20375279 10.1073/pnas.0912478107 PMC2867922

[hipo23139-bib-0179] Geisler, C. , Robbe, D. , Zugaro, M. , Sirota, A. , & Buzsaki, G. (2007). Hippocampal place cell assemblies are speed‐controlled oscillators. Proceedings of the National Academy of Sciences of the United States of America, 104(19), 8149–8154. 17470808 10.1073/pnas.0610121104 PMC1876586

[hipo23139-bib-0180] Ghiretti, A. E. , & Paradis, S. (2014). Molecular mechanisms of activity‐dependent changes in dendritic morphology: role of RGK proteins. Trends in Neurosciences, 37(7), 399–407. 24910262 10.1016/j.tins.2014.05.003 PMC4113564

[hipo23139-bib-0181] Gjorgjieva, J. , Drion, G. , & Marder, E. (2016). Computational implications of biophysical diversity and multiple timescales in neurons and synapses for circuit performance. Current Opinion in Neurobiology, 37, 44–52. 26774694 10.1016/j.conb.2015.12.008 PMC4860045

[hipo23139-bib-0182] Golding, N. L. , Mickus, T. J. , Katz, Y. , Kath, W. L. , & Spruston, N. (2005). Factors mediating powerful voltage attenuation along CA1 pyramidal neuron dendrites. The Journal of Physiology, 568(Pt 1), 69–82. 16002454 10.1113/jphysiol.2005.086793 PMC1474764

[hipo23139-bib-0183] Golding, N. L. , & Oertel, D. (2012). Synaptic integration in dendrites: exceptional need for speed. The Journal of Physiology, 590(Pt 22), 5563–5569. 22930273 10.1113/jphysiol.2012.229328 PMC3528977

[hipo23139-bib-0184] Golding, N. L. , Staff, N. P. , & Spruston, N. (2002). Dendritic spikes as a mechanism for cooperative long‐term potentiation. Nature, 418(6895), 326–331. 12124625 10.1038/nature00854

[hipo23139-bib-0185] Goldman, M. S. , Golowasch, J. , Marder, E. , & Abbott, L. F. (2001). Global structure, robustness, and modulation of neuronal models. The Journal of Neuroscience, 21(14), 5229–5238. 11438598 10.1523/JNEUROSCI.21-14-05229.2001 PMC6762863

[hipo23139-bib-0186] Goutagny, R. , Jackson, J. , & Williams, S. (2009). Self‐generated theta oscillations in the hippocampus. Nature Neuroscience, 12(12), 1491–1493. 19881503 10.1038/nn.2440

[hipo23139-bib-0187] Grant, S. G. (2012). Synaptopathies: diseases of the synaptome. Current Opinion in Neurobiology, 22(3), 522–529. 22409856 10.1016/j.conb.2012.02.002

[hipo23139-bib-0188] Grashow, R. , Brookings, T. , & Marder, E. (2010). Compensation for variable intrinsic neuronal excitability by circuit‐synaptic interactions. The Journal of Neuroscience, 30(27), 9145–9156. 20610748 10.1523/JNEUROSCI.0980-10.2010 PMC2913134

[hipo23139-bib-0189] Grienberger, C. , Milstein, A. D. , Bittner, K. C. , Romani, S. , & Magee, J. C. (2017). Inhibitory suppression of heterogeneously tuned excitation enhances spatial coding in CA1 place cells. Nature Neuroscience, 20(3), 417–426. 28114296 10.1038/nn.4486

[hipo23139-bib-0190] Grosmark, A. D. , Mizuseki, K. , Pastalkova, E. , Diba, K. , & Buzsaki, G. (2012). REM sleep reorganizes hippocampal excitability. Neuron, 75(6), 1001–1007. 22998869 10.1016/j.neuron.2012.08.015 PMC3608095

[hipo23139-bib-0191] Grubb, M. S. , & Burrone, J. (2010a). Activity‐dependent relocation of the axon initial segment fine‐tunes neuronal excitability. Nature, 465(7301), 1070–1074. 20543823 10.1038/nature09160 PMC3196626

[hipo23139-bib-0192] Grubb, M. S. , & Burrone, J. (2010b). Building and maintaining the axon initial segment. Current Opinion in Neurobiology, 20(4), 481–488. 20537529 10.1016/j.conb.2010.04.012 PMC3191460

[hipo23139-bib-0193] Grubb, M. S. , Shu, Y. , Kuba, H. , Rasband, M. N. , Wimmer, V. C. , & Bender, K. J. (2011). Short‐ and long‐term plasticity at the axon initial segment. The Journal of Neuroscience, 31(45), 16049–16055. 22072655 10.1523/JNEUROSCI.4064-11.2011 PMC3232445

[hipo23139-bib-0194] Gu, N. , Vervaeke, K. , Hu, H. , & Storm, J. F. (2005). Kv7/KCNQ/M and HCN/h, but not KCa2/SK channels, contribute to the somatic medium after‐hyperpolarization and excitability control in CA1 hippocampal pyramidal cells. The Journal of Physiology, 566(Pt 3), 689–715. 15890705 10.1113/jphysiol.2005.086835 PMC1464792

[hipo23139-bib-0195] Guckenheimer, J. , & Holmes, P. J. (1983). Nonlinear Oscillations, Dynamical Systems, and Bifurcations of Vector Fields. New York: Springer.

[hipo23139-bib-0196] Gurnett, C. A. , & Campbell, K. P. (1996). Transmembrane auxiliary subunits of voltage‐dependent ion channels. The Journal of Biological Chemistry, 271(45), 27975–27978. 8910401 10.1074/jbc.271.45.27975

[hipo23139-bib-0197] Halassa, M. M. , Fellin, T. , & Haydon, P. G. (2007). The tripartite synapse: roles for gliotransmission in health and disease. Trends in Molecular Medicine, 13(2), 54–63. 17207662 10.1016/j.molmed.2006.12.005

[hipo23139-bib-0198] Halassa, M. M. , & Haydon, P. G. (2010). Integrated brain circuits: astrocytic networks modulate neuronal activity and behavior. Annual Review of Physiology, 72, 335–355. 10.1146/annurev-physiol-021909-135843 PMC3117429 20148679

[hipo23139-bib-0199] Hammond, R. S. , Lin, L. , Sidorov, M. S. , Wikenheiser, A. M. , & Hoffman, D. A. (2008). Protein kinase a mediates activity‐dependent Kv4.2 channel trafficking. The Journal of Neuroscience, 28(30), 7513–7519. 18650329 10.1523/JNEUROSCI.1951-08.2008 PMC2665045

[hipo23139-bib-0200] Hanse, E. , & Gustafsson, B. (1994). TEA elicits two distinct potentiations of synaptic transmission in the CA1 region of the hippocampal slice. The Journal of Neuroscience, 14(8), 5028–5034. 7913959 10.1523/JNEUROSCI.14-08-05028.1994 PMC6577179

[hipo23139-bib-0201] Hanus, C. , & Schuman, E. M. (2013). Proteostasis in complex dendrites. Nature Reviews. Neuroscience, 14(9), 638–648. 23900412 10.1038/nrn3546

[hipo23139-bib-0202] Hartley, T. , Lever, C. , Burgess, N. , & O'Keefe, J. (2014). Space in the brain: how the hippocampal formation supports spatial cognition. Philosophical Transactions of the Royal Society of London. Series B, Biological Sciences, 369(1635), 20120510. 24366125 10.1098/rstb.2012.0510 PMC3866435

[hipo23139-bib-0203] Harvey, C. D. , Collman, F. , Dombeck, D. A. , & Tank, D. W. (2009). Intracellular dynamics of hippocampal place cells during virtual navigation. Nature, 461(7266), 941–946. 19829374 10.1038/nature08499 PMC2771429

[hipo23139-bib-0204] Haydon, P. G. , & Carmignoto, G. (2006). Astrocyte control of synaptic transmission and neurovascular coupling. Physiological Reviews, 86(3), 1009–1031. 16816144 10.1152/physrev.00049.2005

[hipo23139-bib-0205] He, C. , Chen, F. , Li, B. , & Hu, Z. (2014). Neurophysiology of HCN channels: from cellular functions to multiple regulations. Progress in Neurobiology, 112, 1–23. 24184323 10.1016/j.pneurobio.2013.10.001

[hipo23139-bib-0206] Heine, M. , Heck, J. , Ciuraszkiewicz, A. , & Bikbaev, A. (2019). Dynamic compartmentalization of calcium channel signalling in neurons. Neuropharmacology. 10.1016/j.neuropharm.2019.02.038 30851307

[hipo23139-bib-0207] Henderson, J. A. , & Gong, P. (2018). Functional mechanisms underlie the emergence of a diverse range of plasticity phenomena. PLoS Computational Biology, 14(11), e1006590. 30419014 10.1371/journal.pcbi.1006590 PMC6258383

[hipo23139-bib-0208] Hengen, K. B. , Torrado Pacheco, A. , McGregor, J. N. , Van Hooser, S. D. , & Turrigiano, G. G. (2016). Neuronal Firing Rate Homeostasis Is Inhibited by Sleep and Promoted by Wake. Cell, 165(1), 180–191. 26997481 10.1016/j.cell.2016.01.046 PMC4809041

[hipo23139-bib-0209] Henneberger, C. , Papouin, T. , Oliet, S. H. , & Rusakov, D. A. (2010). Long‐term potentiation depends on release of D‐serine from astrocytes. Nature, 463(7278), 232–236. 20075918 10.1038/nature08673 PMC2807667

[hipo23139-bib-0210] Higley, M. J. , & Sabatini, B. L. (2012). Calcium signaling in dendritic spines. Cold Spring Harbor Perspectives in Biology, 4(4), a005686. 22338091 10.1101/cshperspect.a005686 PMC3312680

[hipo23139-bib-0211] Hille, B. (2001). Ion Channels of Excitable Membranes. Sunderland, Massachusetts: Sinauer Associates, Inc.

[hipo23139-bib-0212] Hines, M. L. , Morse, T. , Migliore, M. , Carnevale, N. T. , & Shepherd, G. M. (2004). ModelDB: A Database to Support Computational Neuroscience. Journal of Computational Neuroscience, 17(1), 7–11. 15218350 10.1023/B:JCNS.0000023869.22017.2e PMC3732827

[hipo23139-bib-0213] Hobbs, K. H. , & Hooper, S. L. (2008). Using complicated, wide dynamic range driving to develop models of single neurons in single recording sessions. Journal of Neurophysiology, 99(4), 1871–1883. 18256169 10.1152/jn.00032.2008

[hipo23139-bib-0214] Hodgkin, A. L. , & Huxley, A. F. (1952). A quantitative description of membrane current and its application to conduction and excitation in nerve. The Journal of Physiology, 117(4), 500–544. 12991237 10.1113/jphysiol.1952.sp004764 PMC1392413

[hipo23139-bib-0215] Hoffman, D. A. , & Johnston, D. (1999). Neuromodulation of dendritic action potentials. Journal of Neurophysiology, 81(1), 408–411. 9914302 10.1152/jn.1999.81.1.408

[hipo23139-bib-0216] Hoffman, D. A. , Magee, J. C. , Colbert, C. M. , & Johnston, D. (1997). K+ channel regulation of signal propagation in dendrites of hippocampal pyramidal neurons. Nature, 387(6636), 869–875. 9202119 10.1038/43119

[hipo23139-bib-0217] Hoffman, D. A. , Sprengel, R. , & Sakmann, B. (2002). Molecular dissection of hippocampal theta‐burst pairing potentiation. Proceedings of the National Academy of Sciences of the United States of America, 99(11), 7740–7745. 12032353 10.1073/pnas.092157999 PMC124338

[hipo23139-bib-0218] Holzer, P. (2009). Acid‐sensitive ion channels and receptors. In B. Canning & D. Spina (Eds.), Sensory Nerves Handbook of Experimental Pharmacology, Berlin, Heidelberg: Springer, 194, 283–332. 10.1007/978-3-540-79090-7_9 PMC4359897 19655111

[hipo23139-bib-0219] Hong, S. , Ratte, S. , Prescott, S. A. , & De Schutter, E. (2012). Single neuron firing properties impact correlation‐based population coding. The Journal of Neuroscience, 32(4), 1413–1428. 22279226 10.1523/JNEUROSCI.3735-11.2012 PMC3571732

[hipo23139-bib-0220] Honnuraiah, S. , & Narayanan, R. (2013). A calcium‐dependent plasticity rule for HCN channels maintains activity homeostasis and stable synaptic learning. PLoS One, 8(2), e55590. 23390543 10.1371/journal.pone.0055590 PMC3563588

[hipo23139-bib-0221] Hu, H. , Vervaeke, K. , Graham, L. J. , & Storm, J. F. (2009). Complementary theta resonance filtering by two spatially segregated mechanisms in CA1 hippocampal pyramidal neurons. The Journal of Neuroscience, 29(46), 14472–14483. 19923281 10.1523/JNEUROSCI.0187-09.2009 PMC6665813

[hipo23139-bib-0222] Hu, H. , Vervaeke, K. , & Storm, J. F. (2002). Two forms of electrical resonance at theta frequencies, generated by M‐current, h‐current and persistent Na+ current in rat hippocampal pyramidal cells. The Journal of Physiology, 545(Pt 3), 783–805. 12482886 10.1113/jphysiol.2002.029249 PMC2290731

[hipo23139-bib-0223] Hu, H. , Vervaeke, K. , & Storm, J. F. (2007). M‐channels (Kv7/KCNQ channels) that regulate synaptic integration, excitability, and spike pattern of CA1 pyramidal cells are located in the perisomatic region. The Journal of Neuroscience, 27(8), 1853–1867. 17314282 10.1523/JNEUROSCI.4463-06.2007 PMC6673553

[hipo23139-bib-0224] Huang, C. S. , Shi, S. H. , Ule, J. , Ruggiu, M. , Barker, L. A. , Darnell, R. B. , … Jan, L. Y. (2005). Common molecular pathways mediate long‐term potentiation of synaptic excitation and slow synaptic inhibition. Cell, 123(1), 105–118. 16213216 10.1016/j.cell.2005.07.033

[hipo23139-bib-0225] Huang, Y. Y. , & Malenka, R. C. (1993). Examination of TEA‐induced synaptic enhancement in area CA1 of the hippocampus: the role of voltage‐dependent Ca2+ channels in the induction of LTP. The Journal of Neuroscience, 13(2), 568–576. 8381168 10.1523/JNEUROSCI.13-02-00568.1993 PMC6576643

[hipo23139-bib-0226] Huber, K. M. , Kayser, M. S. , & Bear, M. F. (2000). Role for rapid dendritic protein synthesis in hippocampal mGluR‐dependent long‐term depression. Science, 288(5469), 1254–1257. 10818003 10.1126/science.288.5469.1254

[hipo23139-bib-0227] Huber, K. M. , Mauk, M. D. , & Kelly, P. T. (1995). Distinct LTP induction mechanisms: contribution of NMDA receptors and voltage‐dependent calcium channels. Journal of Neurophysiology, 73(1), 270–279. 7714571 10.1152/jn.1995.73.1.270

[hipo23139-bib-0228] Hulme, S. R. , Jones, O. D. , & Abraham, W. C. (2013). Emerging roles of metaplasticity in behaviour and disease. Trends in Neurosciences, 36(6), 353–362. 23602195 10.1016/j.tins.2013.03.007

[hipo23139-bib-0229] Hutcheon, B. , & Yarom, Y. (2000). Resonance, oscillation and the intrinsic frequency preferences of neurons. Trends in Neurosciences, 23(5), 216–222. 10782127 10.1016/s0166-2236(00)01547-2

[hipo23139-bib-0230] Huxter, J. , Burgess, N. , & O'Keefe, J. (2003). Independent rate and temporal coding in hippocampal pyramidal cells. Nature, 425(6960), 828–832. 14574410 10.1038/nature02058 PMC2677642

[hipo23139-bib-0231] Ibata, K. , Sun, Q. , & Turrigiano, G. G. (2008). Rapid synaptic scaling induced by changes in postsynaptic firing. Neuron, 57(6), 819–826. 18367083 10.1016/j.neuron.2008.02.031

[hipo23139-bib-0232] Ikegaya, Y. , Kim, J. A. , Baba, M. , Iwatsubo, T. , Nishiyama, N. , & Matsuki, N. (2001). Rapid and reversible changes in dendrite morphology and synaptic efficacy following NMDA receptor activation: implication for a cellular defense against excitotoxicity. Journal of Cell Science, 114(Pt 22), 4083–4093. 11739640 10.1242/jcs.114.22.4083

[hipo23139-bib-0233] Isom, L. L. , De Jongh, K. S. , & Catterall, W. A. (1994). Auxiliary subunits of voltage‐gated ion channels. Neuron, 12(6), 1183–1194. 7516685 10.1016/0896-6273(94)90436-7

[hipo23139-bib-0234] Jaffe, D. B. , Johnston, D. , Lasser‐Ross, N. , Lisman, J. E. , Miyakawa, H. , & Ross, W. N. (1992). The spread of Na+ spikes determines the pattern of dendritic Ca2+ entry into hippocampal neurons. Nature, 357(6375), 244–246. 1350327 10.1038/357244a0

[hipo23139-bib-0235] James, W. (1890). The principles of psychology. NY: Henry Holt & Co.

[hipo23139-bib-0236] Jaramillo, J. , & Kempter, R. (2017). Phase precession: a neural code underlying episodic memory? Current Opinion in Neurobiology, 43, 130–138. 28390862 10.1016/j.conb.2017.02.006

[hipo23139-bib-0237] Jazayeri, M. , & Afraz, A. (2017). Navigating the Neural Space in Search of the Neural Code. Neuron, 93(5), 1003–1014. 28279349 10.1016/j.neuron.2017.02.019

[hipo23139-bib-0238] Jedlicka, P. , Benuskova, L. , & Abraham, W. C. (2015). A Voltage‐Based STDP Rule Combined with Fast BCM‐Like Metaplasticity Accounts for LTP and Concurrent "Heterosynaptic" LTD in the Dentate Gyrus In Vivo. PLoS Computational Biology, 11(11), e1004588. 26544038 10.1371/journal.pcbi.1004588 PMC4636250

[hipo23139-bib-0239] Jedlicka, P. , & Deller, T. (2017). Understanding the role of synaptopodin and the spine apparatus in Hebbian synaptic plasticity ‐ New perspectives and the need for computational modeling. Neurobiology of Learning and Memory, 138, 21–30. 27470091 10.1016/j.nlm.2016.07.023

[hipo23139-bib-0240] Jensen, C. S. , Rasmussen, H. B. , & Misonou, H. (2011). Neuronal trafficking of voltage‐gated potassium channels. Molecular and Cellular Neurosciences, 48(4), 288–297. 21627990 10.1016/j.mcn.2011.05.007

[hipo23139-bib-0241] Jerng, H. H. , Pfaffinger, P. J. , & Covarrubias, M. (2004). Molecular physiology and modulation of somatodendritic A‐type potassium channels. Molecular and Cellular Neurosciences, 27(4), 343–369. 15555915 10.1016/j.mcn.2004.06.011

[hipo23139-bib-0242] Johnston, D. , Christie, B. R. , Frick, A. , Gray, R. , Hoffman, D. A. , Schexnayder, L. K. , … Yuan, L. L. (2003). Active dendrites, potassium channels and synaptic plasticity. Philosophical Transactions of the Royal Society of London. Series B, Biological Sciences, 358(1432), 667–674. 12740112 10.1098/rstb.2002.1248 PMC1693145

[hipo23139-bib-0243] Johnston, D. , Frick, A. , & Poolos, N. (2016). Dendrites and disease In Stuart G., Spruston N., & Hausser M. (Eds.), Dendrites (3rd ed.). New York, NY: Oxford University Press.

[hipo23139-bib-0244] Johnston, D. , Magee, J. C. , Colbert, C. M. , & Cristie, B. R. (1996). Active properties of neuronal dendrites. Annual Review of Neuroscience, 19, 165–186. 10.1146/annurev.ne.19.030196.001121 8833440

[hipo23139-bib-0245] Johnston, D. , & Narayanan, R. (2008). Active dendrites: colorful wings of the mysterious butterflies. Trends in Neurosciences, 31(6), 309–316. 18471907 10.1016/j.tins.2008.03.004

[hipo23139-bib-0246] Johnston, D. , Williams, S. , Jaffe, D. , & Gray, R. (1992). NMDA‐receptor‐independent long‐term potentiation. Annual Review of Physiology, 54, 489–505. 10.1146/annurev.ph.54.030192.002421 1314043

[hipo23139-bib-0247] Jonas, E. , & Kording, K. P. (2017). Could a Neuroscientist Understand a Microprocessor? PLoS Computational Biology, 13(1), e1005268. 28081141 10.1371/journal.pcbi.1005268 PMC5230747

[hipo23139-bib-0248] Jorntell, H. , & Hansel, C. (2006). Synaptic memories upside down: bidirectional plasticity at cerebellar parallel fiber‐Purkinje cell synapses. Neuron, 52(2), 227–238. 17046686 10.1016/j.neuron.2006.09.032

[hipo23139-bib-0249] Joshi, N. J. , Tononi, G. , & Koch, C. (2013). The minimal complexity of adapting agents increases with fitness. PLoS Computational Biology, 9(7), e1003111. 23874168 10.1371/journal.pcbi.1003111 PMC3708884

[hipo23139-bib-0250] Jung, S. C. , Kim, J. , & Hoffman, D. A. (2008). Rapid, bidirectional remodeling of synaptic NMDA receptor subunit composition by A‐type K+ channel activity in hippocampal CA1 pyramidal neurons. Neuron, 60(4), 657–671. 19038222 10.1016/j.neuron.2008.08.029 PMC2637039

[hipo23139-bib-0251] Jungenitz, T. , Beining, M. , Radic, T. , Deller, T. , Cuntz, H. , Jedlicka, P. , & Schwarzacher, S. W. (2018). Structural homo‐ and heterosynaptic plasticity in mature and adult newborn rat hippocampal granule cells. Proceedings of the National Academy of Sciences of the United States of America, 115(20), E4670–E4679. 29712871 10.1073/pnas.1801889115 PMC5960324

[hipo23139-bib-0252] Kajikawa, Y. , & Schroeder, C. E. (2011). How local is the local field potential? Neuron, 72(5), 847–858. 22153379 10.1016/j.neuron.2011.09.029 PMC3240862

[hipo23139-bib-0253] Kalantzis, G. , & Shouval, H. Z. (2009). Structural plasticity can produce metaplasticity. PLoS One, 4(11), e8062. 19956610 10.1371/journal.pone.0008062 PMC2779489

[hipo23139-bib-0254] Kamondi, A. , Acsady, L. , Wang, X. J. , & Buzsaki, G. (1998). Theta oscillations in somata and dendrites of hippocampal pyramidal cells in vivo: activity‐dependent phase‐precession of action potentials. Hippocampus, 8(3), 244–261. 9662139 10.1002/(SICI)1098-1063(1998)8:3<244::AID-HIPO7>3.0.CO;2-J

[hipo23139-bib-0255] Kandel, E. R. (2001). The molecular biology of memory storage: a dialogue between genes and synapses. Science, 294(5544), 1030–1038. 11691980 10.1126/science.1067020

[hipo23139-bib-0256] Kandel, E. R. , Dudai, Y. , & Mayford, M. R. (2014). The molecular and systems biology of memory. Cell, 157(1), 163–186. 24679534 10.1016/j.cell.2014.03.001

[hipo23139-bib-0257] Katz, P. S. (2016). Evolution of central pattern generators and rhythmic behaviours. Philosophical Transactions of the Royal Society of London. Series B, Biological Sciences, 371(1685), 20150057. 26598733 10.1098/rstb.2015.0057 PMC4685589

[hipo23139-bib-0258] Katzner, S. , Nauhaus, I. , Benucci, A. , Bonin, V. , Ringach, D. L. , & Carandini, M. (2009). Local origin of field potentials in visual cortex. Neuron, 61(1), 35–41. 19146811 10.1016/j.neuron.2008.11.016 PMC2730490

[hipo23139-bib-0259] Kennedy, M. B. (2000). Signal‐processing machines at the postsynaptic density. Science, 290(5492), 750–754. 11052931 10.1126/science.290.5492.750

[hipo23139-bib-0260] Kennedy, M. B. , Beale, H. C. , Carlisle, H. J. , & Washburn, L. R. (2005). Integration of biochemical signalling in spines. Nature Reviews. Neuroscience, 6(6), 423–434. 15928715 10.1038/nrn1685

[hipo23139-bib-0261] Khakh, B. S. , & Sofroniew, M. V. (2015). Diversity of astrocyte functions and phenotypes in neural circuits. Nature Neuroscience, 18(7), 942–952. 26108722 10.1038/nn.4043 PMC5258184

[hipo23139-bib-0262] Kholodenko, B. N. (2006). Cell‐signalling dynamics in time and space. Nature Reviews. Molecular Cell Biology, 7(3), 165–176. 16482094 10.1038/nrm1838 PMC1679905

[hipo23139-bib-0263] Kim, C. S. , Brager, D. H. , & Johnston, D. (2018). Perisomatic changes in h‐channels regulate depressive behaviors following chronic unpredictablestress. Molecular Psychiatry, 23, 892–903. 28416809 10.1038/mp.2017.28 PMC5647208

[hipo23139-bib-0264] Kim, C. S. , & Johnston, D. (2015). A1 adenosine receptor‐mediated GIRK channels contribute to the resting conductance of CA1 neurons in the dorsal hippocampus. Journal of Neurophysiology, 113(7), 2511–2523. 25652929 10.1152/jn.00951.2014 PMC4416607

[hipo23139-bib-0265] Kim, J. , Jung, S. C. , Clemens, A. M. , Petralia, R. S. , & Hoffman, D. A. (2007). Regulation of dendritic excitability by activity‐dependent trafficking of the A‐type K+ channel subunit Kv4.2 in hippocampal neurons. Neuron, 54(6), 933–947. 17582333 10.1016/j.neuron.2007.05.026 PMC1950443

[hipo23139-bib-0266] Kim, J. , Wei, D. S. , & Hoffman, D. A. (2005). Kv4 potassium channel subunits control action potential repolarization and frequency‐dependent broadening in rat hippocampal CA1 pyramidal neurones. The Journal of Physiology, 569(Pt 1), 41–57. 16141270 10.1113/jphysiol.2005.095042 PMC1464206

[hipo23139-bib-0267] Kim, S. J. , & Linden, D. J. (2007). Ubiquitous plasticity and memory storage. Neuron, 56(4), 582–592. 18031678 10.1016/j.neuron.2007.10.030

[hipo23139-bib-0268] Kim, Y. , Hsu, C. L. , Cembrowski, M. S. , Mensh, B. D. , & Spruston, N. (2015). Dendritic sodium spikes are required for long‐term potentiation at distal synapses on hippocampal pyramidal neurons. eLife, 4, e06414. 10.7554/eLife.06414 PMC4576155 26247712

[hipo23139-bib-0269] Kitano, H. (2007). Towards a theory of biological robustness. Molecular Systems Biology, 3, 137. 17882156 10.1038/msb4100179 PMC2013924

[hipo23139-bib-0270] Klausberger, T. , & Somogyi, P. (2008). Neuronal diversity and temporal dynamics: the unity of hippocampal circuit operations. Science, 321(5885), 53–57. 18599766 10.1126/science.1149381 PMC4487503

[hipo23139-bib-0271] Koester, H. J. , & Johnston, D. (2005). Target cell‐dependent normalization of transmitter release at neocortical synapses. Science, 308(5723), 863–866. 15774725 10.1126/science.1100815

[hipo23139-bib-0272] Korte, M. , & Schmitz, D. (2016). Cellular and System Biology of Memory: Timing, Molecules, and Beyond. Physiological Reviews, 96(2), 647–693. 26960344 10.1152/physrev.00010.2015

[hipo23139-bib-0273] Kotaleski, J. H. , & Blackwell, K. T. (2010). Modelling the molecular mechanisms of synaptic plasticity using systems biology approaches. Nature Reviews. Neuroscience, 11(4), 239–251. 20300102 10.1038/nrn2807 PMC4831053

[hipo23139-bib-0274] Krakauer, J. W. , Ghazanfar, A. A. , Gomez‐Marin, A. , MacIver, M. A. , & Poeppel, D. (2017). Neuroscience Needs Behavior: Correcting a Reductionist Bias. Neuron, 93(3), 480–490. 28182904 10.1016/j.neuron.2016.12.041

[hipo23139-bib-0275] Krichmar, J. L. , Nasuto, S. J. , Scorcioni, R. , Washington, S. D. , & Ascoli, G. A. (2002). Effects of dendritic morphology on CA3 pyramidal cell electrophysiology: a simulation study. Brain Research, 941(1‐2), 11–28. 12031543 10.1016/s0006-8993(02)02488-5

[hipo23139-bib-0276] Kullmann, D. M. (2002). The neuronal channelopathies. Brain, 125(Pt 6), 1177–1195. 12023309 10.1093/brain/awf130

[hipo23139-bib-0277] Lai, H. C. , & Jan, L. Y. (2006). The distribution and targeting of neuronal voltage‐gated ion channels. Nature Reviews. Neuroscience, 7(7), 548–562. 16791144 10.1038/nrn1938

[hipo23139-bib-0278] Lamprecht, R. , & LeDoux, J. (2004). Structural plasticity and memory. Nature Reviews. Neuroscience, 5(1), 45–54. 14708003 10.1038/nrn1301

[hipo23139-bib-0279] Larkman, A. U. , & Jack, J. J. (1995). Synaptic plasticity: hippocampal LTP. Current Opinion in Neurobiology, 5(3), 324–334. 7580155 10.1016/0959-4388(95)80045-x

[hipo23139-bib-0280] Larsen, R. S. , & Sjostrom, P. J. (2015). Synapse‐type‐specific plasticity in local circuits. Current Opinion in Neurobiology, 35, 127–135. 26310110 10.1016/j.conb.2015.08.001 PMC5280068

[hipo23139-bib-0281] Larson, J. , Wong, D. , & Lynch, G. (1986). Patterned stimulation at the theta frequency is optimal for the induction of hippocampal long‐term potentiation. Brain Research, 368(2), 347–350. 3697730 10.1016/0006-8993(86)90579-2

[hipo23139-bib-0282] Lau, C. G. , & Zukin, R. S. (2007). NMDA receptor trafficking in synaptic plasticity and neuropsychiatric disorders. Nature Reviews. Neuroscience, 8(6), 413–426. 17514195 10.1038/nrn2153

[hipo23139-bib-0283] Lazebnik, Y. (2002). Can a biologist fix a radio?‐‐Or, what I learned while studying apoptosis. Cancer Cell, 2(3), 179–182. 12242150 10.1016/s1535-6108(02)00133-2

[hipo23139-bib-0284] Lee, D. , Lin, B. J. , & Lee, A. K. (2012). Hippocampal place fields emerge upon single‐cell manipulation of excitability during behavior. Science, 337(6096), 849–853. 22904011 10.1126/science.1221489

[hipo23139-bib-0285] Lee, H. Y. , & Jan, L. Y. (2012). Fragile X syndrome: mechanistic insights and therapeutic avenues regarding the role of potassium channels. Current Opinion in Neurobiology, 22(5), 887–894. 22483378 10.1016/j.conb.2012.03.010 PMC3393774

[hipo23139-bib-0286] Lee, S. H. , & Dan, Y. (2012). Neuromodulation of brain states. Neuron, 76(1), 209–222. 23040816 10.1016/j.neuron.2012.09.012 PMC3579548

[hipo23139-bib-0287] Lehmann‐Horn F , Jurkat‐Rott K . 1999 Voltage‐Gated Ion Channels and Hereditary Disease. Physiol Rev(4):1317‐1372. 10.1152/physrev.1999.79.4.1317 10508236

[hipo23139-bib-0288] Lein, E. S. , Hawrylycz, M. J. , Ao, N. , Ayres, M. , Bensinger, A. , Bernard, A. , et al. (2007). Genome‐wide atlas of gene expression in the adult mouse brain. Nature, 445(7124), 168–176. 17151600 10.1038/nature05453

[hipo23139-bib-0289] LeMasson, G. , Marder, E. , & Abbott, L. F. (1993). Activity‐dependent regulation of conductances in model neurons. Science, 259(5103), 1915–1917. 8456317 10.1126/science.8456317

[hipo23139-bib-0290] Leonardo, A. (2005). Degenerate coding in neural systems. Journal of Comparative Physiology. A, Neuroethology, Sensory, Neural, and Behavioral Physiology, 191(11), 995–1010. 10.1007/s00359-005-0026-0 16252121

[hipo23139-bib-0291] Lerche, H. , Shah, M. , Beck, H. , Noebels, J. , Johnston, D. , & Vincent, A. (2013). Ion channels in genetic and acquired forms of epilepsy. The Journal of Physiology, 591(Pt 4), 753–764. 23090947 10.1113/jphysiol.2012.240606 PMC3591694

[hipo23139-bib-0292] Levitan, I. , & Barrantes, F. (2012). Cholesterol Regulation of Ion Channels and Receptors. Hoboken, New Jersey: John Wiley & Sons.

[hipo23139-bib-0293] Levitan, I. B. (1994). Modulation of ion channels by protein phosphorylation and dephosphorylation. Annual Review of Physiology, 56, 193–212. 10.1146/annurev.ph.56.030194.001205 7516643

[hipo23139-bib-0294] Lewis, A. S. , Vaidya, S. P. , Blaiss, C. A. , Liu, Z. , Stoub, T. R. , Brager, D. H. , et al. (2011). Deletion of the hyperpolarization‐activated cyclic nucleotide‐gated channel auxiliary subunit TRIP8b impairs hippocampal Ih localization and function and promotes antidepressant behavior in mice. The Journal of Neuroscience, 31(20), 7424–7440. 21593326 10.1523/JNEUROSCI.0936-11.2011 PMC3169171

[hipo23139-bib-0295] Lin, L. , Sun, W. , Kung, F. , Dell'Acqua, M. L. , & Hoffman, D. A. (2011). AKAP79/150 impacts intrinsic excitability of hippocampal neurons through phospho‐regulation of A‐type K+ channel trafficking. The Journal of Neuroscience, 31(4), 1323–1332. 21273417 10.1523/JNEUROSCI.5383-10.2011 PMC3035425

[hipo23139-bib-0296] Lin, L. , Sun, W. , Wikenheiser, A. M. , Kung, F. , & Hoffman, D. A. (2010). KChIP4a regulates Kv4.2 channel trafficking through PKA phosphorylation. Molecular and Cellular Neurosciences, 43(3), 315–325. 20045463 10.1016/j.mcn.2009.12.005 PMC2823810

[hipo23139-bib-0297] Lin, M. T. , Lujan, R. , Watanabe, M. , Adelman, J. P. , & Maylie, J. (2008). SK2 channel plasticity contributes to LTP at Schaffer collateral‐CA1 synapses. Nature Neuroscience, 11(2), 170–177. 18204442 10.1038/nn2041 PMC2613806

[hipo23139-bib-0298] Linden, H. , Tetzlaff, T. , Potjans, T. C. , Pettersen, K. H. , Grun, S. , Diesmann, M. , & Einevoll, G. T. (2011). Modeling the spatial reach of the LFP. Neuron, 72(5), 859–872. 22153380 10.1016/j.neuron.2011.11.006

[hipo23139-bib-0299] Lisman, J. (1989). A mechanism for the Hebb and the anti‐Hebb processes underlying learning and memory. Proceedings of the National Academy of Sciences of the United States of America, 86(23), 9574–9578. 2556718 10.1073/pnas.86.23.9574 PMC298540

[hipo23139-bib-0300] Lisman, J. (2005). The theta/gamma discrete phase code occuring during the hippocampal phase precession may be a more general brain coding scheme. Hippocampus, 15(7), 913–922. 16161035 10.1002/hipo.20121

[hipo23139-bib-0301] Lisman, J. , Schulman, H. , & Cline, H. (2002). The molecular basis of CaMKII function in synaptic and behavioural memory. Nature Reviews. Neuroscience, 3(3), 175–190. 11994750 10.1038/nrn753

[hipo23139-bib-0302] Lisman, J. , Yasuda, R. , & Raghavachari, S. (2012). Mechanisms of CaMKII action in long‐term potentiation. Nature Reviews. Neuroscience, 13(3), 169–182. 22334212 10.1038/nrn3192 PMC4050655

[hipo23139-bib-0303] Lisman, J. E. (2001). Three Ca2+ levels affect plasticity differently: the LTP zone, the LTD zone and no man's land. The Journal of Physiology, 532(Pt 2), 285. 11306649 10.1111/j.1469-7793.2001.0285f.x PMC2278561

[hipo23139-bib-0304] Lisman, J. E. , & Jensen, O. (2013). The theta‐gamma neural code. Neuron, 77(6), 1002–1016. 23522038 10.1016/j.neuron.2013.03.007 PMC3648857

[hipo23139-bib-0305] Llinas, R. R. (1988). The intrinsic electrophysiological properties of mammalian neurons: insights into central nervous system function. Science, 242(4886), 1654–1664. 3059497 10.1126/science.3059497

[hipo23139-bib-0306] London, M. , Roth, A. , Beeren, L. , Hausser, M. , & Latham, P. E. (2010). Sensitivity to perturbations in vivo implies high noise and suggests rate coding in cortex. Nature, 466(7302), 123–127. 20596024 10.1038/nature09086 PMC2898896

[hipo23139-bib-0307] Losick, R. , & Desplan, C. (2008). Stochasticity and cell fate. Science, 320(5872), 65–68. 18388284 10.1126/science.1147888 PMC2605794

[hipo23139-bib-0308] Losonczy, A. , Makara, J. K. , & Magee, J. C. (2008). Compartmentalized dendritic plasticity and input feature storage in neurons. Nature, 452(7186), 436–441. 18368112 10.1038/nature06725

[hipo23139-bib-0309] Losonczy, A. , Zemelman, B. V. , Vaziri, A. , & Magee, J. C. (2010). Network mechanisms of theta related neuronal activity in hippocampal CA1 pyramidal neurons. Nature Neuroscience, 13(8), 967–972. 20639875 10.1038/nn.2597 PMC2921679

[hipo23139-bib-0310] Lujan, R. , Maylie, J. , & Adelman, J. P. (2009). New sites of action for GIRK and SK channels. Nature Reviews. Neuroscience, 10(7), 475–480. 19543219 10.1038/nrn2668

[hipo23139-bib-0311] Luo, L. , & Flanagan, J. G. (2007). Development of continuous and discrete neural maps. Neuron, 56(2), 284–300. 17964246 10.1016/j.neuron.2007.10.014

[hipo23139-bib-0312] Luo, L. , & O'Leary, D. D. (2005). Axon retraction and degeneration in development and disease. Annual Review of Neuroscience, 28, 127–156. 10.1146/annurev.neuro.28.061604.135632 16022592

[hipo23139-bib-0313] Lynch, G. , Larson, J. , Kelso, S. , Barrionuevo, G. , & Schottler, F. (1983). Intracellular injections of EGTA block induction of hippocampal long‐term potentiation. Nature, 305(5936), 719–721. 6415483 10.1038/305719a0

[hipo23139-bib-0314] Lynch, G. S. , Dunwiddie, T. , & Gribkoff, V. (1977). Heterosynaptic depression: a postsynaptic correlate of long‐term potentiation. Nature, 266(5604), 737–739. 195211 10.1038/266737a0

[hipo23139-bib-0315] Lynch, M. A. (2004). Long‐term potentiation and memory. Physiological Reviews, 84(1), 87–136. 14715912 10.1152/physrev.00014.2003

[hipo23139-bib-0316] Ma'ayan, A. , Jenkins, S. L. , Neves, S. , Hasseldine, A. , Grace, E. , Dubin‐Thaler, B. , et al. (2005). Formation of regulatory patterns during signal propagation in a Mammalian cellular network. Science, 309(5737), 1078–1083. 16099987 10.1126/science.1108876 PMC3032439

[hipo23139-bib-0317] Magee, J. C. (2000). Dendritic integration of excitatory synaptic input. Nature Reviews. Neuroscience, 1(3), 181–190. 11257906 10.1038/35044552

[hipo23139-bib-0318] Magee, J. C. (2001). Dendritic mechanisms of phase precession in hippocampal CA1 pyramidal neurons. Journal of Neurophysiology, 86(1), 528–532. 11431530 10.1152/jn.2001.86.1.528

[hipo23139-bib-0319] Magee, J. C. , & Cook, E. P. (2000). Somatic EPSP amplitude is independent of synapse location in hippocampal pyramidal neurons. Nature Neuroscience, 3(9), 895–903. 10966620 10.1038/78800

[hipo23139-bib-0320] Magee, J. C. , & Johnston, D. (1997). A synaptically controlled, associative signal for Hebbian plasticity in hippocampal neurons. Science, 275(5297), 209–213. 8985013 10.1126/science.275.5297.209

[hipo23139-bib-0321] Mainen, Z. F. , & Sejnowski, T. J. (1996). Influence of dendritic structure on firing pattern in model neocortical neurons. Nature, 382(6589), 363–366. 8684467 10.1038/382363a0

[hipo23139-bib-0322] Majewski, L. , & Kuznicki, J. (2015). SOCE in neurons: Signaling or just refilling? Biochimica et Biophysica Acta, 1853(9), 1940–1952. 25646572 10.1016/j.bbamcr.2015.01.019

[hipo23139-bib-0323] Majewski, L. , Maciag, F. , Boguszewski, P. M. , Wasilewska, I. , Wiera, G. , Wojtowicz, T. , … Kuznicki, J. (2017). Overexpression of STIM1 in neurons in mouse brain improves contextual learning and impairs long‐term depression. Biochim Biophys Acta Mol Cell Res, 1864(6), 1071–1087. 10.1016/j.bbamcr.2016.11.025 27913207

[hipo23139-bib-0324] Malenka, R. C. , Lancaster, B. , & Zucker, R. S. (1992). Temporal limits on the rise in postsynaptic calcium required for the induction of long‐term potentiation. Neuron, 9(1), 121–128. 1632966 10.1016/0896-6273(92)90227-5

[hipo23139-bib-0325] Malik, R. , Dougherty, K. A. , Parikh, K. , Byrne, C. , & Johnston, D. (2016). Mapping the electrophysiological and morphological properties of CA1 pyramidal neurons along the longitudinal hippocampal axis. Hippocampus, 26(3), 341–361. 26333017 10.1002/hipo.22526 PMC4760884

[hipo23139-bib-0326] Malik, R. , & Johnston, D. (2017). Dendritic GIRK Channels Gate the Integration Window, Plateau Potentials, and Induction of Synaptic Plasticity in Dorsal But Not Ventral CA1 Neurons. The Journal of Neuroscience, 37(14), 3940–3955. 28280255 10.1523/JNEUROSCI.2784-16.2017 PMC5394901

[hipo23139-bib-0327] Malinow, R. , Schulman, H. , & Tsien, R. W. (1989). Inhibition of postsynaptic PKC or CaMKII blocks induction but not expression of LTP. Science, 245(4920), 862–866. 2549638 10.1126/science.2549638

[hipo23139-bib-0328] Manninen, T. , Hituri, K. , Kotaleski, J. H. , Blackwell, K. T. , & Linne, M. L. (2010). Postsynaptic signal transduction models for long‐term potentiation and depression. Frontiers in Computational Neuroscience, 4, 152. 21188161 10.3389/fncom.2010.00152 PMC3006457

[hipo23139-bib-0329] Marder, E. (1998). From biophysics to models of network function. Annual Review of Neuroscience, 21, 25–45. 10.1146/annurev.neuro.21.1.25 9530490

[hipo23139-bib-0330] Marder, E. (2011). Variability, compensation, and modulation in neurons and circuits. Proceedings of the National Academy of Sciences of the United States of America, 108(Suppl 3), 15542–15548. 21383190 10.1073/pnas.1010674108 PMC3176600

[hipo23139-bib-0331] Marder, E. (2012). Neuromodulation of neuronal circuits: back to the future. Neuron, 76(1), 1–11. 23040802 10.1016/j.neuron.2012.09.010 PMC3482119

[hipo23139-bib-0332] Marder, E. , Abbott, L. F. , Turrigiano, G. G. , Liu, Z. , & Golowasch, J. (1996). Memory from the dynamics of intrinsic membrane currents. Proceedings of the National Academy of Sciences of the United States of America, 93(24), 13481–13486. 8942960 10.1073/pnas.93.24.13481 PMC33634

[hipo23139-bib-0333] Marder, E. , & Goaillard, J. M. (2006). Variability, compensation and homeostasis in neuron and network function. Nature Reviews. Neuroscience, 7(7), 563–574. 16791145 10.1038/nrn1949

[hipo23139-bib-0334] Marder, E. , Goeritz, M. L. , & Otopalik, A. G. (2015). Robust circuit rhythms in small circuits arise from variable circuit components and mechanisms. Current Opinion in Neurobiology, 31, 156–163. 25460072 10.1016/j.conb.2014.10.012 PMC4375070

[hipo23139-bib-0335] Marder, E. , O'Leary, T. , & Shruti, S. (2014). Neuromodulation of circuits with variable parameters: single neurons and small circuits reveal principles of state‐dependent and robust neuromodulation. Annual Review of Neuroscience, 37, 329–346. 10.1146/annurev-neuro-071013-013958 25032499

[hipo23139-bib-0336] Marder, E. , & Taylor, A. L. (2011). Multiple models to capture the variability in biological neurons and networks. Nature Neuroscience, 14(2), 133–138. 21270780 10.1038/nn.2735 PMC3686573

[hipo23139-bib-0337] Marder, E. , & Thirumalai, V. (2002). Cellular, synaptic and network effects of neuromodulation. Neural Networks, 15(4‐6), 479–493. 12371506 10.1016/s0893-6080(02)00043-6

[hipo23139-bib-0338] Markram, H. , Lubke, J. , Frotscher, M. , & Sakmann, B. (1997). Regulation of synaptic efficacy by coincidence of postsynaptic APs and EPSPs. Science, 275(5297), 213–215. 8985014 10.1126/science.275.5297.213

[hipo23139-bib-0339] Marr, D. (1971). Simple memory: a theory for archicortex. Philosophical Transactions of the Royal Society of London. Series B, Biological Sciences, 262(841), 23–81. 4399412 10.1098/rstb.1971.0078

[hipo23139-bib-0340] Martin, S. J. , Grimwood, P. D. , & Morris, R. G. (2000). Synaptic plasticity and memory: an evaluation of the hypothesis. Annual Review of Neuroscience, 23, 649–711. 10.1146/annurev.neuro.23.1.649 10845078

[hipo23139-bib-0341] Martinez, J. L., Jr. , & Derrick, B. E. (1996). Long‐term potentiation and learning. Annual Review of Psychology, 47, 173–203. 10.1146/annurev.psych.47.1.173 8624136

[hipo23139-bib-0342] Matsuzaki, M. , Honkura, N. , Ellis‐Davies, G. , & Kasai, H. (2004). Structural basis of long‐term potentiation in single dendritic spines. Nature, 429(6993), 761–766. 15190253 10.1038/nature02617 PMC4158816

[hipo23139-bib-0343] Mauro, A. (1961). Anomalous impedance, a phenomenological property of time‐variant resistance. An analytic review. Biophysical Journal, 1, 353–372. 13768450 10.1016/s0006-3495(61)86894-x PMC1366353

[hipo23139-bib-0344] Mauro, A. , Conti, F. , Dodge, F. , & Schor, R. (1970). Subthreshold behavior and phenomenological impedance of the squid giant axon. The Journal of General Physiology, 55(4), 497–523. 5435782 10.1085/jgp.55.4.497 PMC2203007

[hipo23139-bib-0345] Mayford, M. , Siegelbaum, S. A. , & Kandel, E. R. (2012). Synapses and memory storage. Cold Spring Harbor Perspectives in Biology, 4(6), a005751. 10.1101/cshperspect.a005751 PMC3367555 22496389

[hipo23139-bib-0346] Mehta, M. R. , Lee, A. K. , & Wilson, M. A. (2002). Role of experience and oscillations in transforming a rate code into a temporal code. Nature, 417(6890), 741–746. 12066185 10.1038/nature00807

[hipo23139-bib-0347] Migliore, M. , De Simone, G. , & Migliore, R. (2015). Effect of the initial synaptic state on the probability to induce long‐term potentiation and depression. Biophysical Journal, 108(5), 1038–1046. 25762316 10.1016/j.bpj.2014.12.048 PMC4375721

[hipo23139-bib-0348] Migliore, M. , & Migliore, R. (2012). Know your current I(h): interaction with a shunting current explains the puzzling effects of its pharmacological or pathological modulations. PLoS One, 7(5), e36867. 22606301 10.1371/journal.pone.0036867 PMC3350476

[hipo23139-bib-0349] Migliore, M. , & Shepherd, G. M. (2002). Emerging rules for the distributions of active dendritic conductances. Nature Reviews. Neuroscience, 3(5), 362–370. 11988775 10.1038/nrn810

[hipo23139-bib-0350] Migliore, M. , & Shepherd, G. M. (2005). Opinion: an integrated approach to classifying neuronal phenotypes. Nature Reviews. Neuroscience, 6(10), 810–818. 16276357 10.1038/nrn1769

[hipo23139-bib-0351] Migliore, R. , Lupascu, C. A. , Bologna, L. L. , Romani, A. , Courcol, J. D. , Antonel, S. , et al. (2018). The physiological variability of channel density in hippocampal CA1 pyramidal cells and interneurons explored using a unified data‐driven modeling workflow. PLoS Computational Biology, 14(9), e1006423. 30222740 10.1371/journal.pcbi.1006423 PMC6160220

[hipo23139-bib-0352] Miller, K. D. , & MacKay, D. J. C. (1994). The role of constraints in Hebbian learning. Neural Computation, 6(1), 100–126.

[hipo23139-bib-0353] Mishra, P. , & Narayanan, R. (2015). High‐conductance states and A‐type K+ channels are potential regulators of the conductance‐current balance triggered by HCN channels. Journal of Neurophysiology, 113(1), 23–43. 25231614 10.1152/jn.00601.2013

[hipo23139-bib-0354] Mishra, P. , & Narayanan, R. (2019). Disparate forms of heterogeneities and interactions among them drive channel decorrelation in the dentate gyrus: Degeneracy and dominance. Hippocampus, 29(4), 378–403. 30260063 10.1002/hipo.23035 PMC6420062

[hipo23139-bib-0355] Misonou, H. , Mohapatra, D. P. , Park, E. W. , Leung, V. , Zhen, D. , Misonou, K. , … Trimmer, J. S. (2004). Regulation of ion channel localization and phosphorylation by neuronal activity. Nature Neuroscience, 7(7), 711–718. 15195093 10.1038/nn1260

[hipo23139-bib-0356] Mittal, D. , & Narayanan, R. (2018). Degeneracy in the robust expression of spectral selectivity, subthreshold oscillations and intrinsic excitability of entorhinal stellate cells. Journal of Neurophysiology, 120(2), 576–600. 29718802 10.1152/jn.00136.2018 PMC6101195

[hipo23139-bib-0357] Miyakawa, H. , Ross, W. N. , Jaffe, D. , Callaway, J. C. , Lasser‐Ross, N. , Lisman, J. E. , & Johnston, D. (1992). Synaptically activated increases in Ca2+ concentration in hippocampal CA1 pyramidal cells are primarily due to voltage‐gated Ca2+ channels. Neuron, 9(6), 1163–1173. 1361128 10.1016/0896-6273(92)90074-n

[hipo23139-bib-0358] Mizuseki, K. , & Buzsaki, G. (2014). Theta oscillations decrease spike synchrony in the hippocampus and entorhinal cortex. Philosophical Transactions of the Royal Society of London. Series B, Biological Sciences, 369(1635), 20120530. 24366139 10.1098/rstb.2012.0530 PMC3866449

[hipo23139-bib-0359] Montgomery, S. M. , Sirota, A. , & Buzsaki, G. (2008). Theta and gamma coordination of hippocampal networks during waking and rapid eye movement sleep. The Journal of Neuroscience, 28(26), 6731–6741. 18579747 10.1523/JNEUROSCI.1227-08.2008 PMC2596978

[hipo23139-bib-0360] Moosmang, S. , Haider, N. , Klugbauer, N. , Adelsberger, H. , Langwieser, N. , Muller, J. , et al. (2005). Role of hippocampal Cav1.2 Ca2+ channels in NMDA receptor‐independent synaptic plasticity and spatial memory. The Journal of Neuroscience, 25(43), 9883–9892. 16251435 10.1523/JNEUROSCI.1531-05.2005 PMC6725564

[hipo23139-bib-0361] Morris, R. G. (1989). Synaptic plasticity and learning: selective impairment of learning rats and blockade of long‐term potentiation in vivo by the N‐methyl‐D‐aspartate receptor antagonist AP5. The Journal of Neuroscience, 9(9), 3040–3057. 2552039 10.1523/JNEUROSCI.09-09-03040.1989 PMC6569656

[hipo23139-bib-0362] Morris, R. G. , Anderson, E. , Lynch, G. S. , & Baudry, M. (1986). Selective impairment of learning and blockade of long‐term potentiation by an N‐methyl‐D‐aspartate receptor antagonist, AP5. Nature, 319(6056), 774–776. 2869411 10.1038/319774a0

[hipo23139-bib-0363] Morris, R. G. , Garrud, P. , Rawlins, J. N. , & O'Keefe, J. (1982). Place navigation impaired in rats with hippocampal lesions. Nature, 297(5868), 681–683. 7088155 10.1038/297681a0

[hipo23139-bib-0364] Moser, E. I. , Kropff, E. , & Moser, M. B. (2008). Place Cells, Grid Cells, and the Brain's Spatial Representation System. Annual Review of Neuroscience, 31, 69–89. 10.1146/annurev.neuro.31.061307.090723 18284371

[hipo23139-bib-0365] Moser, M. B. , Rowland, D. C. , & Moser, E. I. (2015). Place cells, grid cells, and memory. Cold Spring Harbor Perspectives in Biology, 7(2), a021808. 25646382 10.1101/cshperspect.a021808 PMC4315928

[hipo23139-bib-0366] Mozzachiodi, R. , & Byrne, J. H. (2010). More than synaptic plasticity: role of nonsynaptic plasticity in learning and memory. Trends in Neurosciences, 33(1), 17–26. 19889466 10.1016/j.tins.2009.10.001 PMC2815214

[hipo23139-bib-0367] Much, B. , Wahl‐Schott, C. , Zong, X. , Schneider, A. , Baumann, L. , Moosmang, S. , … Biel, M. (2003). Role of subunit heteromerization and N‐linked glycosylation in the formation of functional hyperpolarization‐activated cyclic nucleotide‐gated channels. The Journal of Biological Chemistry, 278(44), 43781–43786. 12928435 10.1074/jbc.M306958200

[hipo23139-bib-0368] Mukunda, C. L. , & Narayanan, R. (2017). Degeneracy in the regulation of short‐term plasticity and synaptic filtering by presynaptic mechanisms. The Journal of Physiology, 595(8), 2611–2637. 28026868 10.1113/JP273482 PMC5390884

[hipo23139-bib-0369] Mulkey, R. M. , & Malenka, R. C. (1992). Mechanisms underlying induction of homosynaptic long‐term depression in area CA1 of the hippocampus. Neuron, 9(5), 967–975. 1419003 10.1016/0896-6273(92)90248-c

[hipo23139-bib-0370] Nagerl, U. V. , Eberhorn, N. , Cambridge, S. B. , & Bonhoeffer, T. (2004). Bidirectional activity‐dependent morphological plasticity in hippocampal neurons. Neuron, 44(5), 759–767. 15572108 10.1016/j.neuron.2004.11.016

[hipo23139-bib-0371] Naisbitt, S. , Kim, E. , Tu, J. C. , Xiao, B. , Sala, C. , Valtschanoff, J. , … Sheng, M. (1999). Shank, a novel family of postsynaptic density proteins that binds to the NMDA receptor/PSD‐95/GKAP complex and cortactin. Neuron, 23(3), 569–582. 10433268 10.1016/s0896-6273(00)80809-0

[hipo23139-bib-0372] Nakashiba, T. , Young, J. Z. , McHugh, T. J. , Buhl, D. L. , & Tonegawa, S. (2008). Transgenic inhibition of synaptic transmission reveals role of CA3 output in hippocampal learning. Science, 319(5867), 1260–1264. 18218862 10.1126/science.1151120

[hipo23139-bib-0373] Nakazawa, K. , McHugh, T. J. , Wilson, M. A. , & Tonegawa, S. (2004). NMDA receptors, place cells and hippocampal spatial memory. Nature Reviews. Neuroscience, 5(5), 361–372. 15100719 10.1038/nrn1385

[hipo23139-bib-0374] Narayanan, R. , & Chattarji, S. (2010). Computational analysis of the impact of chronic stress on intrinsic and synaptic excitability in the hippocampus. Journal of Neurophysiology, 103(6), 3070–3083. 20457854 10.1152/jn.00913.2009 PMC2888238

[hipo23139-bib-0375] Narayanan, R. , Dougherty, K. J. , & Johnston, D. (2010). Calcium Store Depletion Induces Persistent Perisomatic Increases in the Functional Density of h Channels in Hippocampal Pyramidal Neurons. Neuron, 68(5), 921–935. 21145005 10.1016/j.neuron.2010.11.033 PMC3024579

[hipo23139-bib-0376] Narayanan, R. , & Johnston, D. (2007). Long‐term potentiation in rat hippocampal neurons is accompanied by spatially widespread changes in intrinsic oscillatory dynamics and excitability. Neuron, 56(6), 1061–1075. 18093527 10.1016/j.neuron.2007.10.033 PMC2430016

[hipo23139-bib-0377] Narayanan, R. , & Johnston, D. (2008). The h channel mediates location dependence and plasticity of intrinsic phase response in rat hippocampal neurons. The Journal of Neuroscience, 28(22), 5846–5860. 18509046 10.1523/JNEUROSCI.0835-08.2008 PMC2612942

[hipo23139-bib-0378] Narayanan, R. , & Johnston, D. (2010). The h current is a candidate mechanism for regulating the sliding modification threshold in a BCM‐like synaptic learning rule. Journal of Neurophysiology, 104(2), 1020–1033. 20554832 10.1152/jn.01129.2009 PMC2934916

[hipo23139-bib-0379] Narayanan, R. , & Johnston, D. (2012). Functional maps within a single neuron. Journal of Neurophysiology, 108(9), 2343–2351. 22933729 10.1152/jn.00530.2012 PMC3545169

[hipo23139-bib-0380] Nayfeh, A. H. , & Balachandran, B. (1995). Applied nonlinear dynamics: analytical, computational and experimental methods. Weinheim, Germany: Wiley‐VCH Verlag GmbH.

[hipo23139-bib-0381] Nelson, S. B. , & Turrigiano, G. G. (2008). Strength through diversity. Neuron, 60(3), 477–482. 18995822 10.1016/j.neuron.2008.10.020 PMC4919814

[hipo23139-bib-0382] Ness, T. V. , Remme, M. W. H. , & Einevoll, G. T. (2016). Active subthreshold dendritic conductances shape the local field potential. Journal of Physiology (London), 594(13), 3809–3825. 27079755 10.1113/JP272022 PMC4897029

[hipo23139-bib-0383] Neves, G. , Cooke, S. F. , & Bliss, T. V. (2008). Synaptic plasticity, memory and the hippocampus: a neural network approach to causality. Nature Reviews. Neuroscience, 9(1), 65–75. 18094707 10.1038/nrn2303

[hipo23139-bib-0384] Neves, S. R. , & Iyengar, R. (2009). Models of spatially restricted biochemical reaction systems. The Journal of Biological Chemistry, 284(9), 5445–5449. 18940805 10.1074/jbc.R800058200 PMC2645809

[hipo23139-bib-0385] Neves, S. R. , Tsokas, P. , Sarkar, A. , Grace, E. A. , Rangamani, P. , Taubenfeld, S. M. , et al. (2008). Cell shape and negative links in regulatory motifs together control spatial information flow in signaling networks. Cell, 133(4), 666–680. 18485874 10.1016/j.cell.2008.04.025 PMC2728678

[hipo23139-bib-0386] Nevian, T. , & Sakmann, B. (2006). Spine Ca2+ signaling in spike‐timing‐dependent plasticity. The Journal of Neuroscience, 26(43), 11001–11013. 17065442 10.1523/JNEUROSCI.1749-06.2006 PMC6674669

[hipo23139-bib-0387] Nicholson, E. , & Kullmann, D. M. (2017). T‐type calcium channels contribute to NMDA receptor independent synaptic plasticity in hippocampal regular‐spiking oriens‐alveus interneurons. The Journal of Physiology, 595(11), 3449–3458. 10.1113/JP273695 PMC5451714 28134447

[hipo23139-bib-0388] Nishiyama, M. , Hong, K. , Mikoshiba, K. , Poo, M. M. , & Kato, K. (2000). Calcium stores regulate the polarity and input specificity of synaptic modification. Nature, 408(6812), 584–588. 11117745 10.1038/35046067

[hipo23139-bib-0389] Nolan, M. F. , Malleret, G. , Dudman, J. T. , Buhl, D. L. , Santoro, B. , Gibbs, E. , et al. (2004). A behavioral role for dendritic integration: HCN1 channels constrain spatial memory and plasticity at inputs to distal dendrites of CA1 pyramidal neurons. Cell, 119(5), 719–732. 15550252 10.1016/j.cell.2004.11.020

[hipo23139-bib-0390] Nusser, Z. (2009). Variability in the subcellular distribution of ion channels increases neuronal diversity. Trends in Neurosciences, 32(5), 267–274. 19299025 10.1016/j.tins.2009.01.003

[hipo23139-bib-0391] Nusser, Z. (2012). Differential subcellular distribution of ion channels and the diversity of neuronal function. Current Opinion in Neurobiology, 22(3), 366–371. 22033281 10.1016/j.conb.2011.10.006

[hipo23139-bib-0392] O'Keefe, J. (1976). Place units in the hippocampus of the freely moving rat. Experimental Neurology, 51(1), 78–109. 1261644 10.1016/0014-4886(76)90055-8

[hipo23139-bib-0393] O'Keefe, J. (1979). A review of the hippocampal place cells. Progress in Neurobiology, 13(4), 419–439. 396576 10.1016/0301-0082(79)90005-4

[hipo23139-bib-0394] O'Keefe, J. , & Burgess, N. (1999). Theta activity, virtual navigation and the human hippocampus. Trends in Cognitive Sciences, 3(11), 403–406. 10529792 10.1016/s1364-6613(99)01396-0

[hipo23139-bib-0395] O'Keefe, J. , & Burgess, N. (2005). Dual phase and rate coding in hippocampal place cells: theoretical significance and relationship to entorhinal grid cells. Hippocampus, 15(7), 853–866. 16145693 10.1002/hipo.20115 PMC2677681

[hipo23139-bib-0396] O'Keefe, J. , Burgess, N. , Donnett, J. G. , Jeffery, K. J. , & Maguire, E. A. (1998). Place cells, navigational accuracy, and the human hippocampus. Philosophical Transactions of the Royal Society of London. Series B, Biological Sciences, 353(1373), 1333–1340. 9770226 10.1098/rstb.1998.0287 PMC1692339

[hipo23139-bib-0397] O'Keefe, J. , & Conway, D. H. (1978). Hippocampal place units in the freely moving rat: why they fire where they fire. Experimental Brain Research, 31(4), 573–590. 658182 10.1007/BF00239813

[hipo23139-bib-0398] O'Keefe, J. , & Recce, M. L. (1993). Phase relationship between hippocampal place units and the EEG theta rhythm. Hippocampus, 3(3), 317–330. 8353611 10.1002/hipo.450030307

[hipo23139-bib-0399] O'Leary, T. , & Marder, E. (2014). Mapping neural activation onto behavior in an entire animal. Science, 344(6182), 372–373. 24763581 10.1126/science.1253853

[hipo23139-bib-0400] O'Leary, T. , Williams, A. H. , Caplan, J. S. , & Marder, E. (2013). Correlations in ion channel expression emerge from homeostatic tuning rules. Proceedings of the National Academy of Sciences of the United States of America, 110(28), E2645–E2654. 23798391 10.1073/pnas.1309966110 PMC3710808

[hipo23139-bib-0401] O'Leary, T. , Williams, A. H. , Franci, A. , & Marder, E. (2014). Cell types, network homeostasis, and pathological compensation from a biologically plausible ion channel expression model. Neuron, 82(4), 809–821. 24853940 10.1016/j.neuron.2014.04.002 PMC4109293

[hipo23139-bib-0402] Otchy, T. M. , Wolff, S. B. , Rhee, J. Y. , Pehlevan, C. , Kawai, R. , Kempf, A. , … Olveczky, B. P. (2015). Acute off‐target effects of neural circuit manipulations. Nature, 528(7582), 358–363. 26649821 10.1038/nature16442

[hipo23139-bib-0403] Otmakhov, N. , Tao‐Cheng, J. H. , Carpenter, S. , Asrican, B. , Dosemeci, A. , Reese, T. S. , & Lisman, J. (2004). Persistent accumulation of calcium/calmodulin‐dependent protein kinase II in dendritic spines after induction of NMDA receptor‐dependent chemical long‐term potentiation. The Journal of Neuroscience, 24(42), 9324–9331. 15496668 10.1523/JNEUROSCI.2350-04.2004 PMC6730088

[hipo23139-bib-0404] Otmakhova, N. A. , Otmakhov, N. , Mortenson, L. H. , & Lisman, J. E. (2000). Inhibition of the cAMP pathway decreases early long‐term potentiation at CA1 hippocampal synapses. The Journal of Neuroscience, 20(12), 4446–4451. 10844013 10.1523/JNEUROSCI.20-12-04446.2000 PMC6772463

[hipo23139-bib-0405] Ouyang, Y. , Kantor, D. , Harris, K. M. , Schuman, E. M. , & Kennedy, M. B. (1997). Visualization of the distribution of autophosphorylated calcium/calmodulin‐dependent protein kinase II after tetanic stimulation in the CA1 area of the hippocampus. The Journal of Neuroscience, 17(14), 5416–5427. 9204925 10.1523/JNEUROSCI.17-14-05416.1997 PMC6793833

[hipo23139-bib-0406] Ouyang, Y. , Rosenstein, A. , Kreiman, G. , Schuman, E. M. , & Kennedy, M. B. (1999). Tetanic stimulation leads to increased accumulation of Ca(2+)/calmodulin‐dependent protein kinase II via dendritic protein synthesis in hippocampal neurons. The Journal of Neuroscience, 19(18), 7823–7833. 10479685 10.1523/JNEUROSCI.19-18-07823.1999 PMC6782482

[hipo23139-bib-0407] Padamsey, Z. , Foster, W. J. , & Emptage, N. J. (2019). Intracellular Ca(2+) Release and Synaptic Plasticity: A Tale of Many Stores. The Neuroscientist, 25(3), 208–226. 30014771 10.1177/1073858418785334

[hipo23139-bib-0408] Pannasch, U. , & Rouach, N. (2013). Emerging role for astroglial networks in information processing: from synapse to behavior. Trends in Neurosciences, 36(7), 405–417. 23659852 10.1016/j.tins.2013.04.004

[hipo23139-bib-0409] Panzeri, S. , Harvey, C. D. , Piasini, E. , Latham, P. E. , & Fellin, T. (2017). Cracking the Neural Code for Sensory Perception by Combining Statistics, Intervention, and Behavior. Neuron, 93(3), 491–507. 28182905 10.1016/j.neuron.2016.12.036 PMC5308795

[hipo23139-bib-0410] Paoletti, P. , Bellone, C. , & Zhou, Q. (2013). NMDA receptor subunit diversity: impact on receptor properties, synaptic plasticity and disease. Nature Reviews. Neuroscience, 14(6), 383–400. 23686171 10.1038/nrn3504

[hipo23139-bib-0411] Parekh, A. B. (2008). Ca2+ microdomains near plasma membrane Ca2+ channels: impact on cell function. The Journal of Physiology, 586(13), 3043–3054. 18467365 10.1113/jphysiol.2008.153460 PMC2538792

[hipo23139-bib-0412] Park, C. Y. , Shcheglovitov, A. , & Dolmetsch, R. (2010). The CRAC channel activator STIM1 binds and inhibits L‐type voltage‐gated calcium channels. Science, 330(6000), 101–105. 20929812 10.1126/science.1191027

[hipo23139-bib-0413] Pascual, O. , Casper, K. B. , Kubera, C. , Zhang, J. , Revilla‐Sanchez, R. , Sul, J. Y. , … Haydon, P. G. (2005). Astrocytic purinergic signaling coordinates synaptic networks. Science, 310(5745), 113–116. 16210541 10.1126/science.1116916

[hipo23139-bib-0414] Pastalkova, E. , Itskov, V. , Amarasingham, A. , & Buzsaki, G. (2008). Internally generated cell assembly sequences in the rat hippocampus. Science, 321(5894), 1322–1327. 18772431 10.1126/science.1159775 PMC2570043

[hipo23139-bib-0415] Perea, G. , & Araque, A. (2005). Properties of synaptically evoked astrocyte calcium signal reveal synaptic information processing by astrocytes. The Journal of Neuroscience, 25(9), 2192–2203. 15745945 10.1523/JNEUROSCI.3965-04.2005 PMC6726085

[hipo23139-bib-0416] Perea, G. , & Araque, A. (2007). Astrocytes potentiate transmitter release at single hippocampal synapses. Science, 317(5841), 1083–1086. 17717185 10.1126/science.1144640

[hipo23139-bib-0417] Perea, G. , Gomez, R. , Mederos, S. , Covelo, A. , Ballesteros, J. J. , Schlosser, L. , et al. (2016). Activity‐dependent switch of GABAergic inhibition into glutamatergic excitation in astrocyte‐neuron networks. eLife, 5, e20362. 10.7554/eLife.20362 PMC5231406 28012274

[hipo23139-bib-0418] Perea, G. , Navarrete, M. , & Araque, A. (2009). Tripartite synapses: astrocytes process and control synaptic information. Trends in Neurosciences, 32(8), 421–431. 19615761 10.1016/j.tins.2009.05.001

[hipo23139-bib-0419] Phillips, K. G. , Hardingham, N. R. , & Fox, K. (2008). Postsynaptic action potentials are required for nitric‐oxide‐dependent long‐term potentiation in CA1 neurons of adult GluR1 knock‐out and wild‐type mice. The Journal of Neuroscience, 28(52), 14031–14041. 19109486 10.1523/JNEUROSCI.3984-08.2008 PMC3272298

[hipo23139-bib-0420] Philpot, B. D. , Espinosa, J. S. , & Bear, M. F. (2003). Evidence for altered NMDA receptor function as a basis for metaplasticity in visual cortex. The Journal of Neuroscience, 23(13), 5583–5588. 12843259 10.1523/JNEUROSCI.23-13-05583.2003 PMC6741231

[hipo23139-bib-0421] Philpot, B. D. , Sekhar, A. K. , Shouval, H. Z. , & Bear, M. F. (2001). Visual experience and deprivation bidirectionally modify the composition and function of NMDA receptors in visual cortex. Neuron, 29(1), 157–169. 11182088 10.1016/s0896-6273(01)00187-8

[hipo23139-bib-0422] Pike, F. G. , Goddard, R. S. , Suckling, J. M. , Ganter, P. , Kasthuri, N. , & Paulsen, O. (2000). Distinct frequency preferences of different types of rat hippocampal neurones in response to oscillatory input currents. The Journal of Physiology, 529(Pt 1), 205–213. 11080262 10.1111/j.1469-7793.2000.00205.x PMC2270176

[hipo23139-bib-0423] Podlaski, W. F. , Seeholzer, A. , Groschner, L. N. , Miesenbock, G. , Ranjan, R. , & Vogels, T. P. (2017). Mapping the function of neuronal ion channels in model and experiment. eLife, 6, e22152. 10.7554/eLife.22152 PMC5340531 28267430

[hipo23139-bib-0424] Poolos, N. P. , & Johnston, D. (2012). Dendritic ion channelopathy in acquired epilepsy. Epilepsia, 53(Suppl 9), 32–40. 23216577 10.1111/epi.12033 PMC3531827

[hipo23139-bib-0425] Prakriya, M. , & Lewis, R. S. (2015). Store‐Operated Calcium Channels. Physiological Reviews, 95(4), 1383–1436. 26400989 10.1152/physrev.00020.2014 PMC4600950

[hipo23139-bib-0426] Prinz, A. A. , Billimoria, C. P. , & Marder, E. (2003). Alternative to hand‐tuning conductance‐based models: construction and analysis of databases of model neurons. Journal of Neurophysiology, 90(6), 3998–4015. 12944532 10.1152/jn.00641.2003

[hipo23139-bib-0427] Prinz, A. A. , Bucher, D. , & Marder, E. (2004). Similar network activity from disparate circuit parameters. Nature Neuroscience, 7(12), 1345–1352. 15558066 10.1038/nn1352

[hipo23139-bib-0428] Rajasethupathy, P. , Sankaran, S. , Marshel, J. H. , Kim, C. K. , Ferenczi, E. , Lee, S. Y. , et al. (2015). Projections from neocortex mediate top‐down control of memory retrieval. Nature, 526(7575), 653–659. 26436451 10.1038/nature15389 PMC4825678

[hipo23139-bib-0429] Ranjan, R. , Khazen, G. , Gambazzi, L. , Ramaswamy, S. , Hill, S. L. , Schurmann, F. , & Markram, H. (2011). Channelpedia: an integrative and interactive database for ion channels. Frontiers in Neuroinformatics, 5, 36. 22232598 10.3389/fninf.2011.00036 PMC3248699

[hipo23139-bib-0430] Rathour, R. K. , Malik, R. , & Narayanan, R. (2016). Transient potassium channels augment degeneracy in hippocampal active dendritic spectral tuning. Scientific Reports, 6, 24678. 27094086 10.1038/srep24678 PMC4837398

[hipo23139-bib-0431] Rathour, R. K. , & Narayanan, R. (2012a). Inactivating ion channels augment robustness of subthreshold intrinsic response dynamics to parametric variability in hippocampal model neurons. The Journal of Physiology, 590(Pt 22), 5629–5652. 22930270 10.1113/jphysiol.2012.239418 PMC3528982

[hipo23139-bib-0432] Rathour, R. K. , & Narayanan, R. (2012b). Influence fields: a quantitative framework for representation and analysis of active dendrites. Journal of Neurophysiology, 107(9), 2313–2334. 22262825 10.1152/jn.00846.2011

[hipo23139-bib-0433] Rathour, R. K. , & Narayanan, R. (2014). Homeostasis of functional maps in active dendrites emerges in the absence of individual channelostasis. Proceedings of the National Academy of Sciences of the United States of America, 111(17), E1787–E1796. 24711394 10.1073/pnas.1316599111 PMC4035944

[hipo23139-bib-0434] Ratte, S. , Hong, S. , De Schutter, E. , & Prescott, S. A. (2013). Impact of neuronal properties on network coding: roles of spike initiation dynamics and robust synchrony transfer. Neuron, 78(5), 758–772. 23764282 10.1016/j.neuron.2013.05.030 PMC3753823

[hipo23139-bib-0435] Raymond, C. R. (2007). LTP forms 1, 2 and 3: different mechanisms for the "long" in long‐term potentiation. Trends in Neurosciences, 30(4), 167–175. 17292975 10.1016/j.tins.2007.01.007

[hipo23139-bib-0436] Regehr, W. G. (2012). Short‐term presynaptic plasticity. Cold Spring Harbor Perspectives in Biology, 4(7), a005702. 22751149 10.1101/cshperspect.a005702 PMC3385958

[hipo23139-bib-0437] Regehr, W. G. , Carey, M. R. , & Best, A. R. (2009). Activity‐dependent regulation of synapses by retrograde messengers. Neuron, 63(2), 154–170. 19640475 10.1016/j.neuron.2009.06.021 PMC3251517

[hipo23139-bib-0438] Reimann, M. W. , Anastassiou, C. A. , Perin, R. , Hill, S. L. , Markram, H. , & Koch, C. (2013). A biophysically detailed model of neocortical local field potentials predicts the critical role of active membrane currents. Neuron, 79(2), 375–390. 23889937 10.1016/j.neuron.2013.05.023 PMC3732581

[hipo23139-bib-0439] Reisel, D. , Bannerman, D. M. , Schmitt, W. B. , Deacon, R. M. , Flint, J. , Borchardt, T. , … Rawlins, J. N. (2002). Spatial memory dissociations in mice lacking GluR1. Nature Neuroscience, 5(9), 868–873. 12195431 10.1038/nn910

[hipo23139-bib-0440] Remy, S. , Beck, H. , & Yaari, Y. (2010). Plasticity of voltage‐gated ion channels in pyramidal cell dendrites. Current Opinion in Neurobiology, 20(4), 503–509. 10.1016/j.conb.2010.06.006 20691582

[hipo23139-bib-0441] Reyes, A. D. (2003). Synchrony‐dependent propagation of firing rate in iteratively constructed networks in vitro. Nature Neuroscience, 6(6), 593–599. 12730700 10.1038/nn1056

[hipo23139-bib-0442] Rizzuto, R. , & Pozzan, T. (2006). Microdomains of intracellular Ca2+: molecular determinants and functional consequences. Physiological Reviews, 86(1), 369–408. 16371601 10.1152/physrev.00004.2005

[hipo23139-bib-0443] Robinson, R. B. , & Siegelbaum, S. A. (2003). Hyperpolarization‐activated cation currents: from molecules to physiological function. Annual Review of Physiology, 65, 453–480. 10.1146/annurev.physiol.65.092101.142734 12471170

[hipo23139-bib-0444] Rosenkranz, J. A. , Frick, A. , & Johnston, D. (2009). Kinase‐dependent modification of dendritic excitability after long‐term potentiation. The Journal of Physiology, 587(Pt 1), 115–125. 19001050 10.1113/jphysiol.2008.158816 PMC2670027

[hipo23139-bib-0445] Ross, W. N. (2012). Understanding calcium waves and sparks in central neurons. Nature Reviews. Neuroscience, 13(3), 157–168. 22314443 10.1038/nrn3168 PMC4501263

[hipo23139-bib-0446] Roth‐Alpermann, C. , Morris, R. G. , Korte, M. , & Bonhoeffer, T. (2006). Homeostatic shutdown of long‐term potentiation in the adult hippocampus. Proceedings of the National Academy of Sciences of the United States of America, 103(29), 11039–11044. 16829578 10.1073/pnas.0600894103 PMC1544170

[hipo23139-bib-0447] Royer, S. , Zemelman, B. V. , Losonczy, A. , Kim, J. , Chance, F. , Magee, J. C. , & Buzsaki, G. (2012). Control of timing, rate and bursts of hippocampal place cells by dendritic and somatic inhibition. Nature Neuroscience, 15(5), 769–775. 22446878 10.1038/nn.3077 PMC4919905

[hipo23139-bib-0448] Sabatini, B. L. , Oertner, T. G. , & Svoboda, K. (2002). The life cycle of Ca(2+) ions in dendritic spines. Neuron, 33(3), 439–452. 11832230 10.1016/s0896-6273(02)00573-1

[hipo23139-bib-0449] Sakmann, B. (2017). From single cells and single columns to cortical networks: dendritic excitability, coincidence detection and synaptic transmission in brain slices and brains. Experimental Physiology, 102(5), 489–521. 28139019 10.1113/EP085776 PMC5435930

[hipo23139-bib-0450] Sanes, J. R. , & Lichtman, J. W. (1999). Can molecules explain long‐term potentiation? Nature Neuroscience, 2(7), 597–604. 10404178 10.1038/10154

[hipo23139-bib-0451] Santoro, B. , Chen, S. , Luthi, A. , Pavlidis, P. , Shumyatsky, G. P. , Tibbs, G. R. , & Siegelbaum, S. A. (2000). Molecular and functional heterogeneity of hyperpolarization‐activated pacemaker channels in the mouse CNS. The Journal of Neuroscience, 20(14), 5264–5275. 10884310 10.1523/JNEUROSCI.20-14-05264.2000 PMC6772310

[hipo23139-bib-0452] Santoro, B. , Piskorowski, R. A. , Pian, P. , Hu, L. , Liu, H. , & Siegelbaum, S. A. (2009). TRIP8b splice variants form a family of auxiliary subunits that regulate gating and trafficking of HCN channels in the brain. Neuron, 62(6), 802–813. 19555649 10.1016/j.neuron.2009.05.009 PMC2720631

[hipo23139-bib-0453] Santoro, B. , Wainger, B. J. , & Siegelbaum, S. A. (2004). Regulation of HCN channel surface expression by a novel C‐terminal protein‐protein interaction. The Journal of Neuroscience, 24(47), 10750–10762. 15564593 10.1523/JNEUROSCI.3300-04.2004 PMC6730122

[hipo23139-bib-0454] Sarasso, S. , Boly, M. , Napolitani, M. , Gosseries, O. , Charland‐Verville, V. , Casarotto, S. , et al. (2015). Consciousness and Complexity during Unresponsiveness Induced by Propofol, Xenon, and Ketamine. Current Biology, 25(23), 3099–3105. 26752078 10.1016/j.cub.2015.10.014

[hipo23139-bib-0455] Schomburg, E. W. , Anastassiou, C. A. , Buzsaki, G. , & Koch, C. (2012). The spiking component of oscillatory extracellular potentials in the rat hippocampus. The Journal of Neuroscience, 32(34), 11798–11811. 22915121 10.1523/JNEUROSCI.0656-12.2012 PMC3459239

[hipo23139-bib-0456] Schreiner, C. E. , & Winer, J. A. (2007). Auditory cortex mapmaking: principles, projections, and plasticity. Neuron, 56(2), 356–365. 17964251 10.1016/j.neuron.2007.10.013 PMC2412907

[hipo23139-bib-0457] Schulz, D. J. , Goaillard, J. M. , & Marder, E. (2006). Variable channel expression in identified single and electrically coupled neurons in different animals. Nature Neuroscience, 9(3), 356–362. 16444270 10.1038/nn1639

[hipo23139-bib-0458] Schulz, D. J. , Goaillard, J. M. , & Marder, E. E. (2007). Quantitative expression profiling of identified neurons reveals cell‐specific constraints on highly variable levels of gene expression. Proceedings of the National Academy of Sciences of the United States of America, 104(32), 13187–13191. 17652510 10.1073/pnas.0705827104 PMC1933263

[hipo23139-bib-0459] Scoville, W. B. , & Milner, B. (1957). Loss of recent memory after bilateral hippocampal lesions. Journal of Neurology, Neurosurgery, and Psychiatry, 20(1), 11–21. 10.1136/jnnp.20.1.11 PMC497229 13406589

[hipo23139-bib-0460] Sehgal, M. , Song, C. , Ehlers, V. L. , & Moyer, J. R., Jr. (2013). Learning to learn ‐ intrinsic plasticity as a metaplasticity mechanism for memory formation. Neurobiology of Learning and Memory, 105, 186–199. 23871744 10.1016/j.nlm.2013.07.008 PMC3855019

[hipo23139-bib-0461] Shadlen, M. N. , & Newsome, W. T. (1994). Noise, neural codes and cortical organization. Current Opinion in Neurobiology, 4(4), 569–579. 7812147 10.1016/0959-4388(94)90059-0

[hipo23139-bib-0462] Shadlen, M. N. , & Newsome, W. T. (1995). Is there a signal in the noise? Current Opinion in Neurobiology, 5(2), 248–250. 7620314 10.1016/0959-4388(95)80033-6

[hipo23139-bib-0463] Shadlen, M. N. , & Newsome, W. T. (1998). The variable discharge of cortical neurons: implications for connectivity, computation, and information coding. The Journal of Neuroscience, 18(10), 3870–3896. 9570816 10.1523/JNEUROSCI.18-10-03870.1998 PMC6793166

[hipo23139-bib-0464] Shah, M. M. , Hammond, R. S. , & Hoffman, D. A. (2010). Dendritic ion channel trafficking and plasticity. Trends in Neurosciences, 33(7), 307–316. 20363038 10.1016/j.tins.2010.03.002 PMC2902701

[hipo23139-bib-0465] Sheffield, M. E. , & Dombeck, D. A. (2015). Calcium transient prevalence across the dendritic arbour predicts place field properties. Nature, 517(7533), 200–204. 25363782 10.1038/nature13871 PMC4289090

[hipo23139-bib-0466] Shouval, H. Z. , Bear, M. F. , & Cooper, L. N. (2002). A unified model of NMDA receptor‐dependent bidirectional synaptic plasticity. Proceedings of the National Academy of Sciences of the United States of America, 99(16), 10831–10836. 12136127 10.1073/pnas.152343099 PMC125058

[hipo23139-bib-0467] Siegel, M. , Marder, E. , & Abbott, L. F. (1994). Activity‐dependent current distributions in model neurons. Proceedings of the National Academy of Sciences of the United States of America, 91(24), 11308–11312. 7526395 10.1073/pnas.91.24.11308 PMC45220

[hipo23139-bib-0468] Siegelbaum, S. A. (2000). Presynaptic facilitation by hyperpolarization‐activated pacemaker channels. Nature Neuroscience, 3(2), 101–102. 10649561 10.1038/72038

[hipo23139-bib-0469] Sieghart, W. , & Sperk, G. (2002). Subunit composition, distribution and function of GABA(A) receptor subtypes. Current Topics in Medicinal Chemistry, 2(8), 795–816. 12171572 10.2174/1568026023393507

[hipo23139-bib-0470] Sieling, F. , Bedecarrats, A. , Simmers, J. , Prinz, A. A. , & Nargeot, R. (2014). Differential roles of nonsynaptic and synaptic plasticity in operant reward learning‐induced compulsive behavior. Current Biology, 24(9), 941–950. 24704077 10.1016/j.cub.2014.03.004

[hipo23139-bib-0471] Sierra, A. , Tremblay, M. E. , & Wake, H. (2014). Never‐resting microglia: physiological roles in the healthy brain and pathological implications. Frontiers in Cellular Neuroscience, 8, 240. 25177273 10.3389/fncel.2014.00240 PMC4133768

[hipo23139-bib-0472] Singer, W. , Engel, A. K. , Kreiter, A. K. , Munk, M. H. , Neuenschwander, S. , & Roelfsema, P. R. (1997). Neuronal assemblies: necessity, signature and detectability. Trends in Cognitive Sciences, 1(7), 252–261. 21223920 10.1016/S1364-6613(97)01079-6

[hipo23139-bib-0473] Sinha, M. , & Narayanan, R. (2015). HCN channels enhance spike phase coherence and regulate the phase of spikes and LFPs in the theta‐frequency range. Proceedings of the National Academy of Sciences of the United States of America, 112(17), E2207–E2216. 25870302 10.1073/pnas.1419017112 PMC4418872

[hipo23139-bib-0474] Sjostrom, P. J. , & Nelson, S. B. (2002). Spike timing, calcium signals and synaptic plasticity. Current Opinion in Neurobiology, 12(3), 305–314. 12049938 10.1016/s0959-4388(02)00325-2

[hipo23139-bib-0475] Sjostrom, P. J. , Rancz, E. A. , Roth, A. , & Hausser, M. (2008). Dendritic excitability and synaptic plasticity. Physiological Reviews, 88(2), 769–840. 18391179 10.1152/physrev.00016.2007

[hipo23139-bib-0476] Skaggs, W. E. , McNaughton, B. L. , Wilson, M. A. , & Barnes, C. A. (1996). Theta phase precession in hippocampal neuronal populations and the compression of temporal sequences. Hippocampus, 6(2), 149–172. 8797016 10.1002/(SICI)1098-1063(1996)6:2<149::AID-HIPO6>3.0.CO;2-K

[hipo23139-bib-0477] Sloviter, R. S. (1991). Permanently altered hippocampal structure, excitability, and inhibition after experimental status epilepticus in the rat: the "dormant basket cell" hypothesis and its possible relevance to temporal lobe epilepsy. Hippocampus, 1(1), 41–66. 1688284 10.1002/hipo.450010106

[hipo23139-bib-0478] Smith, M. A. , Ellis‐Davies, G. C. , & Magee, J. C. (2003). Mechanism of the distance‐dependent scaling of Schaffer collateral synapses in rat CA1 pyramidal neurons. The Journal of Physiology, 548(Pt 1), 245–258. 12598591 10.1113/jphysiol.2002.036376 PMC2342790

[hipo23139-bib-0479] Soderling, T. R. , & Derkach, V. A. (2000). Postsynaptic protein phosphorylation and LTP. Trends in Neurosciences, 23(2), 75–80. 10652548 10.1016/s0166-2236(99)01490-3

[hipo23139-bib-0480] Softky, W. (1994). Sub‐millisecond coincidence detection in active dendritic trees. Neuroscience, 58(1), 13–41. 8159289 10.1016/0306-4522(94)90154-6

[hipo23139-bib-0481] Softky, W. R. (1995). Simple codes versus efficient codes. Current Opinion in Neurobiology, 5(2), 239–247. 7620313 10.1016/0959-4388(95)80032-8

[hipo23139-bib-0482] Sporns, O. , Tononi, G. , & Edelman, G. M. (2000). Connectivity and complexity: the relationship between neuroanatomy and brain dynamics. Neural Networks, 13(8‐9), 909–922. 11156201 10.1016/s0893-6080(00)00053-8

[hipo23139-bib-0483] Spruston, N. (2008). Pyramidal neurons: dendritic structure and synaptic integration. Nature Reviews. Neuroscience, 9(3), 206–221. 18270515 10.1038/nrn2286

[hipo23139-bib-0484] Spruston, N. , Jaffe, D. B. , & Johnston, D. (1994). Dendritic attenuation of synaptic potentials and currents: the role of passive membrane properties. Trends in Neurosciences, 17(4), 161–166. 7517596 10.1016/0166-2236(94)90094-9

[hipo23139-bib-0485] Spruston, N. , Jaffe, D. B. , Williams, S. H. , & Johnston, D. (1993). Voltage‐ and space‐clamp errors associated with the measurement of electrotonically remote synaptic events. Journal of Neurophysiology, 70(2), 781–802. 8410172 10.1152/jn.1993.70.2.781

[hipo23139-bib-0486] Squire, L. R. , Stark, C. E. , & Clark, R. E. (2004). The medial temporal lobe. Annual Review of Neuroscience, 27, 279–306. 10.1146/annurev.neuro.27.070203.144130 15217334

[hipo23139-bib-0487] Srikanth, S. , & Narayanan, R. (2015). Variability in State‐Dependent Plasticity of Intrinsic Properties during Cell‐Autonomous Self‐Regulation of Calcium Homeostasis in Hippocampal Model Neurons. eNeuro, 2(4), e0053–15.2015. 10.1523/ENEURO.0053-15.2015 PMC4596012 26464994

[hipo23139-bib-0488] Srivastava, K. H. , Holmes, C. M. , Vellema, M. , Pack, A. R. , Elemans, C. P. , Nemenman, I. , & Sober, S. J. (2017). Motor control by precisely timed spike patterns. Proceedings of the National Academy of Sciences of the United States of America, 114(5), 1171–1176. 28100491 10.1073/pnas.1611734114 PMC5293088

[hipo23139-bib-0489] Staubli, U. , & Lynch, G. (1990). Stable depression of potentiated synaptic responses in the hippocampus with 1‐5 Hz stimulation. Brain Research, 513(1), 113–118. 2350674 10.1016/0006-8993(90)91096-y

[hipo23139-bib-0490] Stelling, J. , Sauer, U. , Szallasi, Z. , Doyle, F. J., 3rd , & Doyle, J. (2004). Robustness of cellular functions. Cell, 118(6), 675–685. 15369668 10.1016/j.cell.2004.09.008

[hipo23139-bib-0491] Stevens, C. F. (1998). A million dollar question: does LTP = memory? Neuron, 20(1), 1–2. 9459434 10.1016/s0896-6273(00)80426-2

[hipo23139-bib-0492] Strogatz, S. H. (2014). Nonlinear Dynamics and Chaos: With Applications to Physics, Biology, Chemisty and Engineering. Boulder, CO: Westview Press.

[hipo23139-bib-0493] Sun, W. , Maffie, J. K. , Lin, L. , Petralia, R. S. , Rudy, B. , & Hoffman, D. A. (2011). DPP6 establishes the A‐type K(+) current gradient critical for the regulation of dendritic excitability in CA1 hippocampal neurons. Neuron, 71(6), 1102–1115. 21943606 10.1016/j.neuron.2011.08.008 PMC3184237

[hipo23139-bib-0494] Sunkin, S. M. , Ng, L. , Lau, C. , Dolbeare, T. , Gilbert, T. L. , Thompson, C. L. , … Dang, C. (2013). Allen Brain Atlas: an integrated spatio‐temporal portal for exploring the central nervous system. Nucleic Acids Research, 41(Database issue), D996–D1008. 23193282 10.1093/nar/gks1042 PMC3531093

[hipo23139-bib-0495] Takahashi, H. , & Magee, J. C. (2009). Pathway interactions and synaptic plasticity in the dendritic tuft regions of CA1 pyramidal neurons. Neuron, 62(1), 102–111. 19376070 10.1016/j.neuron.2009.03.007

[hipo23139-bib-0496] Taxidis, J. , Anastassiou, C. A. , Diba, K. , & Koch, C. (2015). Local Field Potentials Encode Place Cell Ensemble Activation during Hippocampal Sharp Wave Ripples. Neuron, 87(3), 590–604. 26247865 10.1016/j.neuron.2015.07.014 PMC6469679

[hipo23139-bib-0497] Taylor, A. L. , Goaillard, J. M. , & Marder, E. (2009). How multiple conductances determine electrophysiological properties in a multicompartment model. The Journal of Neuroscience, 29(17), 5573–5586. 19403824 10.1523/JNEUROSCI.4438-08.2009 PMC2821064

[hipo23139-bib-0498] Thattai, M. , & van Oudenaarden, A. (2001). Intrinsic noise in gene regulatory networks. Proceedings of the National Academy of Sciences of the United States of America, 98(15), 8614–8619. 11438714 10.1073/pnas.151588598 PMC37484

[hipo23139-bib-0499] Titley, H. K. , Brunel, N. , & Hansel, C. (2017). Toward a Neurocentric View of Learning. Neuron, 95(1), 19–32. 28683265 10.1016/j.neuron.2017.05.021 PMC5519140

[hipo23139-bib-0500] Tobin, A. E. , Van Hooser, S. D. , & Calabrese, R. L. (2006). Creation and reduction of a morphologically detailed model of a leech heart interneuron. Journal of Neurophysiology, 96(4), 2107–2120. 16760352 10.1152/jn.00026.2006 PMC2897741

[hipo23139-bib-0501] Tonnesen, J. , Katona, G. , Rozsa, B. , & Nagerl, U. V. (2014). Spine neck plasticity regulates compartmentalization of synapses. Nature Neuroscience, 17(5), 678–685. 24657968 10.1038/nn.3682

[hipo23139-bib-0502] Tononi, G. , & Cirelli, C. (2006). Sleep function and synaptic homeostasis. Sleep Medicine Reviews, 10(1), 49–62. 16376591 10.1016/j.smrv.2005.05.002

[hipo23139-bib-0503] Tononi, G. , & Edelman, G. M. (1998). Consciousness and complexity. Science, 282(5395), 1846–1851. 9836628 10.1126/science.282.5395.1846

[hipo23139-bib-0504] Tononi, G. , Edelman, G. M. , & Sporns, O. (1998). Complexity and coherency: integrating information in the brain. Trends in Cognitive Sciences, 2(12), 474–484. 21227298 10.1016/s1364-6613(98)01259-5

[hipo23139-bib-0505] Tononi, G. , Sporns, O. , & Edelman, G. M. (1994). A measure for brain complexity: relating functional segregation and integration in the nervous system. Proceedings of the National Academy of Sciences of the United States of America, 91(11), 5033–5037. 8197179 10.1073/pnas.91.11.5033 PMC43925

[hipo23139-bib-0506] Tononi, G. , Sporns, O. , & Edelman, G. M. (1996). A complexity measure for selective matching of signals by the brain. Proceedings of the National Academy of Sciences of the United States of America, 93(8), 3422–3427. 8622951 10.1073/pnas.93.8.3422 PMC39624

[hipo23139-bib-0507] Tononi, G. , Sporns, O. , & Edelman, G. M. (1999). Measures of degeneracy and redundancy in biological networks. Proceedings of the National Academy of Sciences of the United States of America, 96(6), 3257–3262. 10077671 10.1073/pnas.96.6.3257 PMC15929

[hipo23139-bib-0508] Traub, R. D. , Miles, R. , & Wong, R. K. (1989). Model of the origin of rhythmic population oscillations in the hippocampal slice. Science, 243(4896), 1319–1325. 2646715 10.1126/science.2646715

[hipo23139-bib-0509] Triesch, J. (2007). Synergies between intrinsic and synaptic plasticity mechanisms. Neural Computation, 19(4), 885–909. 17348766 10.1162/neco.2007.19.4.885

[hipo23139-bib-0510] Trimmer, J. S. , & Rhodes, K. J. (2004). Localization of voltage‐gated ion channels in mammalian brain. Annual Review of Physiology, 66, 477–519. 10.1146/annurev.physiol.66.032102.113328 14977411

[hipo23139-bib-0511] Tripathy, S. J. , Savitskaya, J. , Burton, S. D. , Urban, N. N. , & Gerkin, R. C. (2014). NeuroElectro: a window to the world's neuron electrophysiology data. Frontiers in Neuroinformatics, 8, 40. 24808858 10.3389/fninf.2014.00040 PMC4010726

[hipo23139-bib-0512] Tsien, J. Z. , Huerta, P. T. , & Tonegawa, S. (1996). The essential role of hippocampal CA1 NMDA receptor‐dependent synaptic plasticity in spatial memory. Cell, 87(7), 1327–1338. 8980238 10.1016/s0092-8674(00)81827-9

[hipo23139-bib-0513] Tsokas, P. , Hsieh, C. , Yao, Y. , Lesburgueres, E. , Wallace, E. J. , Tcherepanov, A. , et al. (2016). Compensation for PKMzeta in long‐term potentiation and spatial long‐term memory in mutant mice. eLife, 5, e14846. 10.7554/eLife.14846 PMC4869915 27187150

[hipo23139-bib-0514] Tu, J. C. , Xiao, B. , Naisbitt, S. , Yuan, J. P. , Petralia, R. S. , Brakeman, P. , et al. (1999). Coupling of mGluR/Homer and PSD‐95 complexes by the Shank family of postsynaptic density proteins. Neuron, 23(3), 583–592. 10433269 10.1016/s0896-6273(00)80810-7

[hipo23139-bib-0515] Tu, J. C. , Xiao, B. , Yuan, J. P. , Lanahan, A. A. , Leoffert, K. , Li, M. , … Worley, P. F. (1998). Homer binds a novel proline‐rich motif and links group 1 metabotropic glutamate receptors with IP3 receptors. Neuron, 21(4), 717–726. 9808459 10.1016/s0896-6273(00)80589-9

[hipo23139-bib-0516] Turrigiano, G. (2007). Homeostatic signaling: the positive side of negative feedback. Current Opinion in Neurobiology, 17(3), 318–324. 17451937 10.1016/j.conb.2007.04.004

[hipo23139-bib-0517] Turrigiano, G. (2011). Too many cooks? Intrinsic and synaptic homeostatic mechanisms in cortical circuit refinement. Annual Review of Neuroscience, 34, 89–103. 10.1146/annurev-neuro-060909-153238 21438687

[hipo23139-bib-0518] Turrigiano, G. , Abbott, L. F. , & Marder, E. (1994). Activity‐dependent changes in the intrinsic properties of cultured neurons. Science, 264(5161), 974–977. 8178157 10.1126/science.8178157

[hipo23139-bib-0519] Turrigiano, G. G. (1999). Homeostatic plasticity in neuronal networks: the more things change, the more they stay the same. Trends in Neurosciences, 22(5), 221–227. 10322495 10.1016/s0166-2236(98)01341-1

[hipo23139-bib-0520] Turrigiano, G. G. (2008). The self‐tuning neuron: synaptic scaling of excitatory synapses. Cell, 135(3), 422–435. 18984155 10.1016/j.cell.2008.10.008 PMC2834419

[hipo23139-bib-0521] Turrigiano, G. G. (2017). The dialectic of Hebb and homeostasis. Philosophical Transactions of the Royal Society of London. Series B, Biological Sciences, 372(1715), 20160258. 10.1098/rstb.2016.0258 PMC5247594 28093556

[hipo23139-bib-0522] Turrigiano, G. G. , & Nelson, S. B. (2000). Hebb and homeostasis in neuronal plasticity. Current Opinion in Neurobiology, 10(3), 358–364. 10851171 10.1016/s0959-4388(00)00091-x

[hipo23139-bib-0523] Turrigiano, G. G. , & Nelson, S. B. (2004). Homeostatic plasticity in the developing nervous system. Nature Reviews. Neuroscience, 5(2), 97–107. 14735113 10.1038/nrn1327

[hipo23139-bib-0524] Tytell, E. D. , Holmes, P. , & Cohen, A. H. (2011). Spikes alone do not behavior make: why neuroscience needs biomechanics. Current Opinion in Neurobiology, 21(5), 816–822. 21683575 10.1016/j.conb.2011.05.017 PMC3183174

[hipo23139-bib-0525] Ulens, C. , & Siegelbaum, S. A. (2003). Regulation of hyperpolarization‐activated HCN channels by cAMP through a gating switch in binding domain symmetry. Neuron, 40(5), 959–970. 14659094 10.1016/s0896-6273(03)00753-0

[hipo23139-bib-0526] Ulens, C. , & Tytgat, J. (2001). Functional heteromerization of HCN1 and HCN2 pacemaker channels. The Journal of Biological Chemistry, 276(9), 6069–6072. 11133998 10.1074/jbc.C000738200

[hipo23139-bib-0527] Vacher, H. , Mohapatra, D. P. , & Trimmer, J. S. (2008). Localization and targeting of voltage‐dependent ion channels in mammalian central neurons. Physiological Reviews, 88(4), 1407–1447. 18923186 10.1152/physrev.00002.2008 PMC2587220

[hipo23139-bib-0528] Vacher, H. , & Trimmer, J. S. (2011). Diverse roles for auxiliary subunits in phosphorylation‐dependent regulation of mammalian brain voltage‐gated potassium channels. Pflügers Archiv, 462(5), 631–643. 21822597 10.1007/s00424-011-1004-8 PMC3371647

[hipo23139-bib-0529] van Rossum, M. C. , Bi, G. Q. , & Turrigiano, G. G. (2000). Stable Hebbian learning from spike timing‐dependent plasticity. The Journal of Neuroscience, 20(23), 8812–8821. 11102489 10.1523/JNEUROSCI.20-23-08812.2000 PMC6773092

[hipo23139-bib-0530] Varzi A. 2016 Mereology In: Zalta EN, editor. The Stanford Encyclopedia of Philosophy. Winter 2016 ed. Pala Alto CA: Metaphysics Research Lab, Stanford University.

[hipo23139-bib-0531] Verkhratsky, A. (2002). The endoplasmic reticulum and neuronal calcium signalling. Cell Calcium, 32(5‐6), 393–404. 12543098 10.1016/s0143416002001896

[hipo23139-bib-0532] Verkhratsky, A. , & Steinhauser, C. (2000). Ion channels in glial cells. Brain Research. Brain Research Reviews, 32(2‐3), 380–412. 10760549 10.1016/s0165-0173(99)00093-4

[hipo23139-bib-0533] Vetere, G. , Kenney, J. W. , Tran, L. M. , Xia, F. , Steadman, P. E. , Parkinson, J. , … Frankland, P. W. (2017). Chemogenetic Interrogation of a Brain‐wide Fear Memory Network in Mice. Neuron, 94(2), 363–374 e4. 28426969 10.1016/j.neuron.2017.03.037

[hipo23139-bib-0534] Vogelstein, J. T. , Park, Y. , Ohyama, T. , Kerr, R. A. , Truman, J. W. , Priebe, C. E. , & Zlatic, M. (2014). Discovery of brainwide neural‐behavioral maps via multiscale unsupervised structure learning. Science, 344(6182), 386–392. 24674869 10.1126/science.1250298

[hipo23139-bib-0535] Volterra, A. , Liaudet, N. , & Savtchouk, I. (2014). Astrocyte Ca(2)(+) signalling: an unexpected complexity. Nature Reviews. Neuroscience, 15(5), 327–335. 24739787 10.1038/nrn3725

[hipo23139-bib-0536] Wagner, A. (2005). Distributed robustness versus redundancy as causes of mutational robustness. BioEssays, 27(2), 176–188. 15666345 10.1002/bies.20170

[hipo23139-bib-0537] Wagner, A. (2008). Robustness and evolvability: a paradox resolved. Proceedings of the Biological Sciences, 275(1630), 91–100. 10.1098/rspb.2007.1137 PMC2562401 17971325

[hipo23139-bib-0538] Wang, H. , & Wagner, J. J. (1999). Priming‐induced shift in synaptic plasticity in the rat hippocampus. Journal of Neurophysiology, 82(4), 2024–2028. 10515995 10.1152/jn.1999.82.4.2024

[hipo23139-bib-0539] Wang, X. J. (2010). Neurophysiological and computational principles of cortical rhythms in cognition. Physiological Reviews, 90(3), 1195–1268. 20664082 10.1152/physrev.00035.2008 PMC2923921

[hipo23139-bib-0540] Wang, X. J. , & Buzsaki, G. (1996). Gamma oscillation by synaptic inhibition in a hippocampal interneuronal network model. The Journal of Neuroscience, 16(20), 6402–6413. 8815919 10.1523/JNEUROSCI.16-20-06402.1996 PMC6578902

[hipo23139-bib-0541] Wang, Y. , Deng, X. , Mancarella, S. , Hendron, E. , Eguchi, S. , Soboloff, J. , … Gill, D. L. (2010). The calcium store sensor, STIM1, reciprocally controls Orai and CaV1.2 channels. Science, 330(6000), 105–109. 20929813 10.1126/science.1191086 PMC3601900

[hipo23139-bib-0542] Wang, Z. , Xu, N. , Wu, C. , Duan, S. , & Poo, M. (2003). Bidirectional changes in spatial dendritic integration accompanying long‐term synaptic modifications. Neuron, 37(3), 463–472. 12575953 10.1016/s0896-6273(02)01189-3

[hipo23139-bib-0543] Watanabe, S. , Hoffman, D. , Migliore, M. , & Johnston, D. (2002). Dendritic K+ channels contribute to spike‐timing dependent long‐term potentiation in hippocampal pyramidal neurons. Proceedings of the National Academy of Sciences of the United States of America, 99(12), 8366–8371. 12048251 10.1073/pnas.122210599 PMC123073

[hipo23139-bib-0544] Weaver, C. M. , & Wearne, S. L. (2008). Neuronal firing sensitivity to morphologic and active membrane parameters. PLoS Computational Biology, 4(1), e11. 18208320 10.1371/journal.pcbi.0040011 PMC2211531

[hipo23139-bib-0545] Weng, G. , Bhalla, U. S. , & Iyengar, R. (1999). Complexity in biological signaling systems. Science, 284(5411), 92–96. 10102825 10.1126/science.284.5411.92 PMC3773983

[hipo23139-bib-0546] Wenthold, R. J. , Prybylowski, K. , Standley, S. , Sans, N. , & Petralia, R. S. (2003). Trafficking of NMDA receptors. Annual Review of Pharmacology and Toxicology, 43, 335–358. 10.1146/annurev.pharmtox.43.100901.135803 12540744

[hipo23139-bib-0547] Whitacre, J. , & Bender, A. (2010). Degeneracy: a design principle for achieving robustness and evolvability. Journal of Theoretical Biology, 263(1), 143–153. 19925810 10.1016/j.jtbi.2009.11.008

[hipo23139-bib-0548] Whitacre, J. M. (2010). Degeneracy: a link between evolvability, robustness and complexity in biological systems. Theoretical Biology & Medical Modelling, 7, 6. 20167097 10.1186/1742-4682-7-6 PMC2830971

[hipo23139-bib-0549] White, L. E. , & Fitzpatrick, D. (2007). Vision and cortical map development. Neuron, 56(2), 327–338. 17964249 10.1016/j.neuron.2007.10.011

[hipo23139-bib-0550] Whitlock, J. R. , Heynen, A. J. , Shuler, M. G. , & Bear, M. F. (2006). Learning induces long‐term potentiation in the hippocampus. Science, 313(5790), 1093–1097. 16931756 10.1126/science.1128134

[hipo23139-bib-0551] Wills, T. J. , Lever, C. , Cacucci, F. , Burgess, N. , & O'Keefe, J. (2005). Attractor dynamics in the hippocampal representation of the local environment. Science, 308(5723), 873–876. 15879220 10.1126/science.1108905 PMC2680068

[hipo23139-bib-0552] Wilson, M. A. , & McNaughton, B. L. (1994). Reactivation of hippocampal ensemble memories during sleep. Science, 265(5172), 676–679. 8036517 10.1126/science.8036517

[hipo23139-bib-0553] Woo, N. H. , Duffy, S. N. , Abel, T. , & Nguyen, P. V. (2003). Temporal spacing of synaptic stimulation critically modulates the dependence of LTP on cyclic AMP‐dependent protein kinase. Hippocampus, 13(2), 293–300. 12699336 10.1002/hipo.10086

[hipo23139-bib-0554] Xiao, B. , Tu, J. C. , & Worley, P. F. (2000). Homer: a link between neural activity and glutamate receptor function. Current Opinion in Neurobiology, 10(3), 370–374. 10851183 10.1016/s0959-4388(00)00087-8

[hipo23139-bib-0555] Yasuda, R. , Nimchinsky, E. A. , Scheuss, V. , Pologruto, T. A. , Oertner, T. G. , Sabatini, B. L. , & Svoboda, K. (2004). Imaging calcium concentration dynamics in small neuronal compartments. Science's STKE, 2004(219), l5. 10.1126/stke.2192004pl5 14872098

[hipo23139-bib-0556] Yeung, L. C. , Shouval, H. Z. , Blais, B. S. , & Cooper, L. N. (2004). Synaptic homeostasis and input selectivity follow from a calcium‐dependent plasticity model. Proceedings of the National Academy of Sciences of the United States of America, 101(41), 14943–14948. 15466713 10.1073/pnas.0405555101 PMC522010

[hipo23139-bib-0557] Yizhar, O. , Fenno, L. E. , Prigge, M. , Schneider, F. , Davidson, T. J. , O'Shea, D. J. , et al. (2011). Neocortical excitation/inhibition balance in information processing and social dysfunction. Nature, 477(7363), 171–178. 21796121 10.1038/nature10360 PMC4155501

[hipo23139-bib-0558] Ylinen, A. , Bragin, A. , Nadasdy, Z. , Jando, G. , Szabo, I. , Sik, A. , & Buzsaki, G. (1995). Sharp wave‐associated high‐frequency oscillation (200 Hz) in the intact hippocampus: network and intracellular mechanisms. The Journal of Neuroscience, 15(1 Pt 1), 30–46. 7823136 10.1523/JNEUROSCI.15-01-00030.1995 PMC6578299

[hipo23139-bib-0559] Ylinen, A. , Soltesz, I. , Bragin, A. , Penttonen, M. , Sik, A. , & Buzsaki, G. (1995). Intracellular correlates of hippocampal theta rhythm in identified pyramidal cells, granule cells, and basket cells. Hippocampus, 5(1), 78–90. 7787949 10.1002/hipo.450050110

[hipo23139-bib-0560] Yu, R. C. , Pesce, C. G. , Colman‐Lerner, A. , Lok, L. , Pincus, D. , Serra, E. , … Brent, R. (2008). Negative feedback that improves information transmission in yeast signalling. Nature, 456(7223), 755–761. 19079053 10.1038/nature07513 PMC2716709

[hipo23139-bib-0561] Yuan, L.‐L. , Adams, J. P. , Swank, M. , Sweatt, J. D. , & Johnston, D. (2002). Protein kinase modulation of dendritic K+ channels in hippocampus involves a mitogen‐activated protein kinase pathway. The Journal of Neuroscience, 22(12), 4860–4868. 12077183 10.1523/JNEUROSCI.22-12-04860.2002 PMC6757742

[hipo23139-bib-0562] Yuste, R. , & Bonhoeffer, T. (2001). Morphological changes in dendritic spines associated with long‐term synaptic plasticity. Annual Review of Neuroscience, 24, 1071–1089. 10.1146/annurev.neuro.24.1.1071 11520928

[hipo23139-bib-0563] Zamanillo, D. , Sprengel, R. , Hvalby, O. , Jensen, V. , Burnashev, N. , Rozov, A. , et al. (1999). Importance of AMPA receptors for hippocampal synaptic plasticity but not for spatial learning. Science, 284(5421), 1805–1811. 10364547 10.1126/science.284.5421.1805

[hipo23139-bib-0564] Zemankovics, R. , Kali, S. , Paulsen, O. , Freund, T. F. , & Hajos, N. (2010). Differences in subthreshold resonance of hippocampal pyramidal cells and interneurons: the role of h‐current and passive membrane characteristics. The Journal of Physiology, 588(Pt 12), 2109–2132. 20421280 10.1113/jphysiol.2009.185975 PMC2905616

[hipo23139-bib-0565] Zenke, F. , Gerstner, W. , & Ganguli, S. (2017). The temporal paradox of Hebbian learning and homeostatic plasticity. Current Opinion in Neurobiology, 43, 166–176. 28431369 10.1016/j.conb.2017.03.015

[hipo23139-bib-0566] Zhang, W. , & Linden, D. J. (2003). The other side of the engram: experience‐driven changes in neuronal intrinsic excitability. Nature Reviews. Neuroscience, 4(11), 885–900. 14595400 10.1038/nrn1248

[hipo23139-bib-0567] Zolles, G. , Wenzel, D. , Bildl, W. , Schulte, U. , Hofmann, A. , Muller, C. S. , et al. (2009). Association with the auxiliary subunit PEX5R/Trip8b controls responsiveness of HCN channels to cAMP and adrenergic stimulation. Neuron, 62(6), 814–825. 19555650 10.1016/j.neuron.2009.05.008

[hipo23139-bib-0568] Zorec, R. , Araque, A. , Carmignoto, G. , Haydon, P. G. , Verkhratsky, A. , & Parpura, V. (2012). Astroglial excitability and gliotransmission: an appraisal of Ca2+ as a signalling route. ASN Neuro, 4(2), e00080. 10.1042/AN20110061 PMC3310306 22313347

[hipo23139-bib-0569] Zucker, R. S. (1989). Short‐term synaptic plasticity. Annual Review of Neuroscience, 12, 13–31. 10.1146/annurev.ne.12.030189.000305 2648947

[hipo23139-bib-0570] Zucker, R. S. (1999). Calcium‐ and activity‐dependent synaptic plasticity. Current Opinion in Neurobiology, 9(3), 305–313. 10395573 10.1016/s0959-4388(99)80045-2

[hipo23139-bib-0571] Zucker, R. S. , & Regehr, W. G. (2002). Short‐term synaptic plasticity. Annual Review of Physiology, 64, 355–405. 10.1146/annurev.physiol.64.092501.114547 11826273

